# FOXM1 expression is significantly associated with chemotherapy resistance and adverse prognosis in non-serous epithelial ovarian cancer patients

**DOI:** 10.1186/s13046-017-0536-y

**Published:** 2017-05-08

**Authors:** Renata A. Tassi, Paola Todeschini, Eric R. Siegel, Stefano Calza, Paolo Cappella, Laura Ardighieri, Moris Cadei, Mattia Bugatti, Chiara Romani, Elisabetta Bandiera, Laura Zanotti, Laura Tassone, Donatella Guarino, Concetta Santonocito, Ettore D. Capoluongo, Luca Beltrame, Eugenio Erba, Sergio Marchini, Maurizio D’Incalci, Carla Donzelli, Alessandro D. Santin, Sergio Pecorelli, Enrico Sartori, Eliana Bignotti, Franco Odicino, Antonella Ravaggi

**Affiliations:** 1 Department of Obstetrics and Gynecology, “Angelo Nocivelli” Institute of Molecular Medicine, University of Brescia, Brescia, Italy; 2 Department of Biostatistics, University of Arkansas for Medical Sciences, Little Rock, AR USA; 3 Department of Molecular and Translational Medicine, Unit of Biostatistics, University of Brescia, Brescia, Italy; 4 Nerviano Medical Sciences Srl, Nerviano, Milan, Italy; 5 Department of Molecular and Translational Medicine, Section of Pathology, University-ASST Spedali Civili of Brescia, Brescia, Italy; 6 Laboratory of Clinical Molecular and Personalized Diagnostics, Institute of Biochemistry and Clinical Biochemistry, Catholic University and Foundation Gemelli Hospital, Rome, Italy; 7 Department of Oncology, IRCCS - “Mario Negri” Institute for Pharmacological Research, Milan, Italy; 8 Department of Obstetrics, Gynecology and Reproductive Sciences, Yale University School of Medicine, New Haven, CT USA; 9 Department of Obstetrics and Gynecology, University of Brescia, Brescia, Italy; 10 Division of Obstetrics and Gynecology, ASST Spedali Civili di Brescia, Brescia, Italy

**Keywords:** Epithelial ovarian cancer, Subtype, Prognosis, Chemoresistance, FOXM1, Cell line, Anticancer drug, Immunohistochemistry, Microarray

## Abstract

**Background:**

Epithelial ovarian cancer (EOC) is a spectrum of different diseases, which makes their treatment a challenge. Forkhead box M1 (FOXM1) is an oncogene aberrantly expressed in many solid cancers including serous EOC, but its role in non-serous EOCs remains undefined. We examined FOXM1 expression and its correlation to prognosis across the three major EOC subtypes, and its role in tumorigenesis and chemo-resistance in vitro.

**Methods:**

Gene signatures were generated by microarray for 14 clear-cell and 26 endometrioid EOCs, and 15 normal endometrium snap-frozen biopsies. Validation of FOXM1 expression was performed by RT–qPCR and immunohistochemistry in the same samples and additionally in 50 high-grade serous EOCs and in their most adequate normal controls (10 luminal fallopian tube and 20 ovarian surface epithelial brushings). Correlations of FOXM1 expression to clinic-pathological parameters and patients’ prognosis were evaluated by Kaplan-Meier and Cox proportional-hazards analyses. OVCAR-3 and two novel deeply characterized EOC cell lines (EOC-CC1 and OSPC2, with clear-cell and serous subtype, respectively) were employed for in vitro studies. Effects of FOXM1 inhibition by transient siRNA transfection were evaluated on cell-proliferation, cell-cycle, colony formation, invasion, and response to conventional first- and second-line anticancer agents, and to the PARP-inhibitor olaparib. Gene signatures of FOXM1-silenced cell lines were generated by microarray and confirmed by RT-qPCR.

**Results:**

A significant FOXM1 mRNA up-regulation was found in EOCs compared to normal controls. FOXM1 protein overexpression significantly correlated to serous histology (*p* = 0.001) and advanced FIGO stage (*p* = 0.004). Multivariate analyses confirmed FOXM1 protein overexpression as an independent indicator of worse disease specific survival in non-serous EOCs, and of shorter time to progression in platinum-resistant cases. FOXM1 downregulation in EOC cell lines inhibited cell growth and clonogenicity, and promoted the cytotoxic effects of platinum compounds, doxorubicin hydrochloride and olaparib. Upon FOXM1 knock-down in EOC-CC1 and OSPC2 cells, microarray and RT-qPCR analyses revealed the deregulation of several common and other unique subtype-specific FOXM1 putative targets involved in cell cycle, metastasis, DNA repair and drug response.

**Conclusions:**

FOXM1 is up-regulated in all three major EOCs subtypes, and is a prognostic biomarker and a potential combinatorial therapeutic target in platinum resistant disease, irrespective of tumor histology.

**Electronic supplementary material:**

The online version of this article (doi:10.1186/s13046-017-0536-y) contains supplementary material, which is available to authorized users.

## Background

Epithelial ovarian cancer (EOC) represents 90% of ovarian cancers, being also associated to very heterogeneous clinical presentation, histological features and therapeutic response [1]. The high mortality rate related to this malignancy reflects its asymptomatic nature, the lack of adequate screening tests, the frequent diagnosis at late stages and the high incidence of chemoresistant recurrences [2]. EOC is classified in five main histological types: high-grade serous (HGS) (70%), endometrioid (10%), clear-cell (10%), mucinous (3%), and low-grade serous carcinomas (<5%). These subtypes are very different entities carrying distinct genetic risk factors, molecular signatures, prognoses and responses to treatment [3, 4]. Moreover, they are supposed to arise from distinct anatomical structures, such as the fimbria or mesothelium for high-grade serous cancers (HGSC) [3, 5], while the origin of both endometrioid and clear-cell EOCs is supposed to be endometrial tissue, passing through the fallopian tube and resulting in endometriosis [3]. Although different histological variants should require specific therapeutic approaches [6], the same treatment plan for all of them, consisting of primary debulking surgery followed by platinum-based chemotherapy, is currently recommended. Advanced EOC patients frequently respond to initial platinum-based chemotherapy, but the majority of them develop platinum-resistant recurrence within 18 months from the end of the upfront therapy and are therefore candidates for second-line treatment [2]. One of the therapeutic opportunities in relapsed EOC is doxorubicin hydrochloride, an anthracycline antitumor antibiotic that inhibits DNA topoisomerase II by inducing double-stranded DNA breaks [7]. Its newer formulation, pegylated liposomal doxorubicin (PLD), represents one of the most effective second-line therapeutic option for recurrent or progressive EOC [7]. Other promising therapeutic approaches aim at inhibiting cell-specific signal transduction pathways and DNA repair mechanisms. Olaparib is the first poly-ADP-ribose polymerase (PARP) inhibitor approved by the United States Food and Drug Administration for use in advanced EOC patients harboring germline or somatic *BRCA1* and *BRCA2* mutations, who have received three or more prior lines of chemotherapy [8]. Moreover, its efficacy also has been evaluated in a subset of recurrent platinum-sensitive non-serous EOC that display defects in the homologous-recombination (HR) pathway of DNA repair [9].

Nonetheless, further advancements in the management of recurrent or persistent disease are still required, especially for drug-resistant EOCs that generally show poor responsiveness to additional cytotoxic therapy. Transcriptional profiling represents a useful tool to identify tissue-specific therapeutic targets that impact on clinical outcome.

Several studies show that the transcription factor Forkhead box M1 (FOXM1) is widely expressed in solid tumors [10], acting as a principal promoter of cell-cycle progression, response to DNA damage and drug resistance [11], where its overexpression confers proliferative advantages to cancer cells [12]. Accordingly, FOXM1 drives the transcription of many downstream cell-cycle checkpoint genes [13], DNA-damage signal transducers and effectors [11]. Since the discovery of FOXM1-pathway activation in HGSC by the Cancer Genome Atlas (TCGA) study [14], its pivotal roles in HGSC initiation and progression [15], stemness and epithelial mesenchymal transition, cisplatin [16] and paclitaxel resistance [17], DNA repair [18], prognosis [19] and therapy [20] have been well documented. However, the expression profile and functional contribution of FOXM1 to non-serous EOC tumorigenesis and drug resistance remain elusive.

In the present investigation, we generated the mRNA signatures of tumor specimens belonging to two EOC patients’ cohorts representing the less common cancer subtypes (endometrioid and clear-cell), and of their normal counterpart (endometrium samples). Next, we explored the trend in FOXM1 expression at mRNA and protein level across clear-cell, endometrioid and HGS EOCs compared to their supposed normal tissue of origin (endometrium, fallopian tube and ovarian surface epithelium, respectively). We investigated the association between FOXM1 protein and patients’ survival in relation to their tumor histology and sensitivity to platinum-based therapy. We determined the functional activity of FOXM1 in EOC cells in terms of cell proliferation, cell-cycle regulation, colony formation, and invasion ability by transient FOXM1 knockdown, using two in-house derived EOC cell lines expressing FOXM1, as in vitro models that accurately represent a metastatic HGSC and a primary clear-cell EOC. HGSC is the most frequent, lethal and extensively studied EOC subtype [14], whereas clear-cell EOC is a rare malignancy that shows both different genetic landscape and clinical behavior compared to other subtypes being strongly characterized by resistance to conventional platinum and/or taxane-based chemotherapy [21].

Since FOXM1 is an emerging master regulator of response to DNA damage [11], in this study we speculated that its inhibition could alter the expression of genes involved in DNA-repair pathways and thus sensitize EOC cells to DNA-damaging agents in vitro. To this end, we tested the efficacy of cisplatin, carboplatin, doxorubicin and olaparib in combination with FOXM1 inhibition in our novel EOC cell lines in vitro. Finally, to elucidate the FOXM1-related gene signature in EOC cell lines, we identified the most relevant gene-expression changes in response to FOXM1 silencing, by microarray experiments.

## Methods

### Patients and clinical information

This study was performed on 90 cases of EOCs diagnosed and treated at the Division of Gynecologic Oncology, Department of Obstetrics and Gynecology, University of Brescia, Italy, between November 2001 and September 2013, following the Declaration of Helsinki set of principles, and with approval by the Research Review Board- the Ethics Committee- of the ASST Spedali Civili, Brescia, Italy (study reference number: NP1284). Normal control tissue samples were obtained from 45 patients undergoing surgery for benign pathologies. Written informed consent was obtained from all patients enrolled.

Patients’ details are reported in Table 1. No patient received preoperative chemotherapy or radiotherapy. Patients were followed from the date of surgery until death or the latest record retrieved, February 2016 (median follow-up, 65 months; range, 6–155 months). Optimal cytoreduction was defined as no macroscopic residual tumor (RT) after primary surgery (RT = 0).

**Table 1:** Clinico pathological features of 90 EOCs and 45 normal control patients

Parameters	CC-EOC	END-EOC	HGSC	Normal controls		
				NE	OSE	TEC
Total number of cases	14	26	50	15	20	10
Age (years)						
Median (range)	57 (34–74)	55 (39–72)	60 (24–84)	57 (50–71)	53 (47–65)	50 (42–55)
Stage						
I-II	11	15	7			
III-IV	3	11	43			
Grade						
High	14	14	50			
Low	0	12	0			
Residual tumor (TR)						
TR = 0 cm	11	19	15			
TR > 0 cm	3	7	35			
Presence of ascites						
Yes	3	15	35			
No	11	11	14			
Na	0	0	1			
Neoplastic citology						
Negative	8	11	8			
Positive	6	11	39			
Not diagnostic	0	4	3			
Lymph nodes						
Negative	11	17	21			
Positive	1	5	17			
Na	2	4	12			
Platinum-based chemotherapy						
Yes	14	23	50			
No	0	3	0			
Response to first-line chemotherapy						
Complete	12	25	37			
Partial	0	0	4			
None	2	1	8			
Na	0	0	1			
Recurrence						
Yes	5	12	30			
No	9	13	7			
Na	0	1	2			
Progression	0	0	11			
Follow-up status						
Ned	8	13	10			
Awd	0	3	3			
Dod	6	8	35			
Did	0	1	2			
Lost	0	1	0			
Platinum status						
Sensitive	10	19	27			
Partially sensitive	0	2	7			
Resistant	4	2	16			
Na	0	3	0			

*OSE* ovarian surface epithelial cells, *TEC* Luminal fallopian tube epithelial cells, *NE* normal endometrial tissue, *Ned* no evidence of disease, *awd* alive with disease, *dod* dead of disease, *did* dead of intercurrent disease, *lost* lost to follow-up

Disease Specific survival (DSS) was defined as the time from surgery to death from disease or the last follow-up. Progression Free Survival (PFS) was calculated from the time of surgery until the first clinical recurrence/progression. Cancer progression was defined according to RECIST 1.1 [22]. The PFI (platinum-free interval) was defined from the last date of platinum dose until progressive disease was documented [23]. EOC patients were clinically defined as “resistant”, “partially sensitive”, and “sensitive” to platinum-based chemotherapy on the basis of their PFIs (<6, 6–12, and >12 months, respectively) [23]. Patients known to be still alive at the time of analysis and patients who died from another disease were censored at the time of their last follow-up.

### Tissue samples collection

Ninety EOC specimens were collected at the time of primary debulking and immediately frozen as previously described [24]. Normal endometrial samples (NE) were obtained from 15 patients undergoing hysterectomy for benign indications.

Ten normal luminal fallopian tube epithelial cells (TEC) and 6 pools of ovarian surface epithelial-cell (OSE) brushings obtained from 20 patients, were separately collected by scraping in 1 ml of physiological saline solution immediately after surgery, as previously described [25].

### Establishment and characterization of the EOC cell lines

Two new EOC cell lines (named OSPC2 and EOC-CC1) were derived in our laboratory from fresh clinical samples. Source-patient characteristics and full method of cell line characterization (immunohistochemical staining, short tandem repeat (STR) DNA profiling, BRCA1/2 sequencing and growth rate analysis) are described in Additional file 1.

### Gene silencing using siRNA

The following Silencer® select pre-designed siRNAs (Thermo Fisher Scientific, Inc. Waltham, MA, USA) were employed for in vitro transient gene knockdown: FOXM1 (ID # s5248), negative control no.1 (AM4636) and KIF11 (Eg5) (ID # AM4639). Based on cell lines’ growth-rate analysis (see Additional file 1), the highest number of cells showing continuing exponential growth after 3 days was selected for silencing assays. The cells were seeded onto 6-well plates and grown to 20% confluency in the absence of antibiotic for 48 h before transfection. The cells were washed once with PBS, and then transfected with either FOXM1-specific siRNA, KIF11 siRNA or scramble siRNA with Lipofectamine RNAiMAX in Opti-MEM medium (ThermoFisher) according to the manufacturer's instructions. After 24 h siRNA transfection, cells were placed in fresh culture medium.

### Total RNA extraction

Total RNA was extracted and purified from 90 EOC snap-frozen biopsies containing at least 70% of tumor epithelial cells, from normal samples (10 TEC and 6 pools of OSE brushings, and 15 fresh-frozen NEs) previously verified to be free of any neoplastic pathology, and from EOC cell lines after siRNA transfection. For both tissues and cells, TRIzolⓇ Reagent (ThermoFisher) was used according to manufacturer’s instructions. Total RNA extraction and quality control were performed as previously reported [24].

### Genechip hybridization

EOC samples of endometrioid (*N* = 26) and clear-cell histology (*N* = 14), as well as NE samples (*N* = 15), were labelled and hybridized to GeneChip® Human Genome U133 Plus 2.0 oligonucleotide microarray chips (Affymetrix, Inc., Santa Clara, CA, USA) following the manufacturer’s protocols. Microarray experiments on scramble- (siControl) and FOXM1-siRNA transfected (siFOXM1) OSPC2 and EOC-CC1 cell lines were performed using the commercially available G4851B human whole GE Microarray kit (SurePrint G3 Human Gene Expression 8 × 60 K v2 Microarray Kit Agilent Technologies) according to manufacturer’s instructions.

### FOXM1 immunohistochemical (IHC) study of clinical samples

To evaluate FOXM1 protein expression level, IHC was performed on matched 90 formalin fixed-paraffin embedded (FFPE) EOC tissues (50 HGSCs, 26 endometrioid and 14 clear-cell EOCs), stored in the Department of Pathology at the University of Brescia, Italy. As controls, specimens obtained from normal ovaries, fallopian tubes and endometria were used. FOXM1 immunostaining was performed using a primary antibody (sc-502, Santa Cruz Biotechnology, Santa Cruz, CA) diluted at 1:160 after pretreatment with microwave in citrate buffer at pH 6.0 (3 cycles of 5 min at 750 Watt). Immunoreactivity for FOXM1 was considered positive in tumor cells showing a nuclear staining, with or without cytoplasmic staining. FOXM1-immunostained slides were digitalized using an Aperio ScanScope CS Slide Scanner (Aperio Technologies, Vista, CA, USA) at 40× magnification, and analyzed using Tissue Studio™ 2.0 workstation (Definiens AG, Munich, Germany). Subsequently, a quantitative scoring algorithm was customized for FOXM1 using commercially available templates from Aperio Technologies and Definiens, and applied in order to quantify the percentage of FOXM1-positive tumor cells and to assess FOXM1 nuclear-staining intensity according to mean brown chromogen intensity. The overall FOXM1 expression “positive index” was determined by multiplying the percentage of the positive cells and the intensity score, then a weighted sum was calculated for each tumor samples. A final immunoreactive score (IRS) from 2 to 90 was calculated.

### Reverse Transcription and Real-Time quantitative PCR (RT-qPCR)

One μg of extracted RNA was reverse-transcribed using random hexamers according to the SuperScript TM II protocol (Invitrogen). The qPCR reactions were performed on CFX96 Touch™ Real-Time PCR Detection System (BIO-RAD Laboratories, Hercules, CA, USA) using the TaqMan Universal PCR master mix and the following Taqman gene expression assays: Hs01073586_m1 (FOXM1), HS99999905_m1 (GAPDH), Hs00188166_m1 (SDHA), Hs00259126_m1 (CCNB1), HS00244740_m1 (CDC25B), Hs00411505_m1 (ASPM), Hs01118845_m1 (CENPF), Hs00921424_m1 (FOXO3), Hs00959834_m1 (XRCC1), Hs00254718_m1 (XRCC4), Hs00998500_g1 (CYR61), Hs00172214_m1 (TOP2A), Hs01037414_m1 (BRCA2), Hs00947967_m1 (RAD51), Hs00766186_m1 (MACC1), Hs01070181_m1 (CEP55), Hs01026371_m1 (CDK6), Hs01548894_m1 (CDK2), Hs00976734_m1 (CXCR4), Hs01548727_m1 (MMP2). Reaction and thermal cycling conditions were performed as previously reported [24]. The comparative threshold cycle (Ct) method was used for the calculation of amplification fold, and the delta-delta Ct method was used to obtain relative gene expression values [26] normalized using three reference genes: glyceraldehyde-3-phosphate dehydrogenase (GAPDH), succinate dehydrogenase complex flavoprotein subunit A (SDHA), and peptidylprolyl isomerase A (PPIA).

FOXM1 isoform-specific RT-qPCR was performed using iTaq Universal SYBR Green Supermix (BIO-RAD) and the following primer pairs: FOXM1c F caattgcccgagcacttggaatca, FOXM1c R tccttcagctagcagcaccttg, FOXM1b F ccaggtgtttaagcagcaga, FOXM1b R tccttcagctagcagcaccttg [27]. An annealing temperature of 60 °C and a total of 45 cycles for all primer pairs were used, and all reactions were run in triplicate. A melting curve was constructed for each primer pair to confirm amplification product specificity. An inter-run calibration sample was used in all plates to correct for the technical variance between the different runs and to compare results from different plates.

### Western blot assay

Whole protein extracts from cells at 24 h following siRNA transfection or untransfection were lysed in NaCl, 1% Nonidet‑40, 50 mM Tris‑HCl (pH 7.5) and Halt Protease Inhibitor Cocktail (Thermo Fisher Scientific), and protein concentrations were determined using a Bio-Rad protein assay system (Bio-Rad). Equivalent amounts of proteins were separated by SDS-PAGE, and then transferred to polyvinylidene difluoride membranes (Bio-Rad). After being blocked in Tris-buffered saline (TBS) containing 5% non-fat milk, the blots were incubated with the following primary antibodies: anti-FOXM1 (clone sc-502, 1:200 dilution, Santa Cruz Biotechnology, Santa Cruz, CA), and anti-β-actin ((20–33), 1:200 dilution) (Sigma-Aldrich, Saint Louis, Missouri, USA), at 4 °C for 12 h, followed by incubation with horseradish peroxidase-conjugated secondary (anti-mouse or anti-rabbit) IgGs at room temperature for 1 h. Signals were detected on BioSpectrum® Imaging System (UVP, LLC, Upland, CA, USA) with the LiteAblot® EXTEND (EuroClone). Images were processed with VisionWorks® LS Image Acquisition and Analysis software (version 7.0.1, UVP, LLC).

### Cell cycle analysis by flow cytometry

Fixed cells were stained with propidium iodide (PI) (50 μg/ml, Sigma) at times 0, 24, 48, and 72 h after transfection with scramble siRNA or specific siRNA targeted for FOXM1 and KIF11. Tests were performed in triplicate, and cell-cycle percentages were measured [28].

For accurate cell-cycle analysis, FOXM1- or scramble-treated cells up to 72 h were exposed to 30 μM bromodeoxyuridine (BrdU) and fixed in 70% ethanol into Matrix™ small tubes. Nuclei were isolated using pepsin/0.1 M HCl mixture and subsequently treated with 2 N HCl for 20 min; BrdU detection was performed after DNA denaturation using FITC Anti-BrdU mAb (BD Biosciences) for 1 h and DNA-content staining using PI (2 μg/ml) for 30 min at room temperature [29]. BrdU analysis and cell-cycle determination were performed using BD CellQuest Pro™ on BD FACSCalibur flow cytometer (BD Biosciences, New Jersey, USA) by acquiring at least 5000 single nuclei, with S-Phase estimation based on DNA-active (BrdU+ S cells) and -inactive (BrdU- S cells) synthesis. G2/M estimation included G2/M (4n) and DNA endoreduplicated cells (8n) [30].

### Clonogenic assay

Following 24 h siRNA transfection, cells were seeded in 6-well plates in 2 ml medium and grown for 7–9 days. Colonies were fixed and stained with 20% ethanol, 1% crystal violet solution and automated counted using the Entry Level image analysis system (Immagini & Computer, Bareggio, Milan, Italy). A background correction was performed and the control-cell colony size (≥50 cells) was established as the minimum for setting the cut-off point [31].

### Cell invasion assay

After 24 h siRNA transfection, cells were seeded on the upper chamber of the BioCoat Matrigel Invasion Chamber (BD Biosciences) at a density of 2 × 10^4^ (OVCAR-3), 1 × 10^5^ (OSPC2), 1.4 × 10^5^ (EOC-CC1) cells/well in serum-free medium. RPMI 10% FBS was applied to the lower chamber as chemoattractant. Following 72 h incubation, the invading cells on the lower surface of the membrane were fixed with methanol, stained with 0.5% crystal violet, photographed and counted in 3 randomly selected fields per well, and the mean number of cells was recorded. The experiments were done in triplicate and repeated four times.

### Drugs

Carboplatin, Cisplatin and Doxorubicin hydrochloride (Sigma) were diluted in culture medium immediately before addition to cell lines. Olaparib (AZD2281, Selleck Chemicals (Boston, MA, USA)) was dissolved in DMSO (Sigma).

### Chemosensitivity and proliferation assays of transfected cell lines

After 24 h siRNA transfection, OSPC2 and EOC-CC1 cells were seeded at 4000 cells per well, in 96-well plates in RPMI 10%FBS and allowed to attach overnight. For the chemosensitivity assays, wells were treated in quintuplicate with 5 serial dilutions of each drug and DMSO as control in a final volume of 200 μL. Drug-free controls were included in each assay. After 72 h, cell viability was determined by CellTiter 96® AQueous One Solution Cell Proliferation Assay (MTS) (Promega Corporation, Madison, WI), according to manufacturer’s instructions. Absorbance was measured by a 96-well plate reader (SpectraMax 340PC, Molecular Devices, Sunnyvale, CA, USA) at a reference wavelength of 490 nm. Each experiment was repeated a minimum of three times. The effect of drugs on cell growth inhibition was assessed as percent cell viability, where vehicle-treated cells were taken as 100% viable.

### Statistical analysis

#### Survival analysis

The prognostic value of FOXM1 was evaluated on serous and non-serous EOC patients’ subgroups. The survival analyses were performed using Cox’s proportional-hazards models [32] with FOXM1 modelled as a bivariate (Low/High) variable. Dichotomization was performed based on time-dependent ROC analysis and the closest-to-(0.1)-corner approach [33]. Kaplan-Meier curves were used to graphically show the survival trends.

#### Analysis of proliferation data

Proliferation data were modelled as normalized counts to a reference and transformed to a logarithm scale. The model was fit by a linear mixed model to account for the variation of technical replicates between independent experiments. Time was expressed on a 24 h scale.

#### Microarray processing

All gene chip analyses are explained in detail in Additional file 2.

From differentially expressed genes, network reconstruction was carried out in siFOXM1 EOC-CC1 and OSPC2 cell lines, respectively, with the Ingenuity Pathway Analysis software (IPA; QIAGEN, USA).

The differentially expressed genes in each cell line were assessed for “enrichment” or over-representation in a set of 111 invasion-related genes (Additional file 2: Table S3) using contingency-table analysis with Fisher’s exact test.

The correlation between microarray and RT-qPCR data for FOXM1 gene expression was evaluated by Spearman rank correlation. The statistical significance of differences between two groups was evaluated by Mann–Whitney *U*-test or by Student’s *t*-test. Colony and invasion assays results were analysed by one-way ANOVA. All the analyses were performed using either the software R (version 3.3.2) or SAS v9.4 software (The SAS Institute, Cary, NC, USA), and employed significance levels of alpha = 0.05 except where previously indicated.

## Results

### FOXM1 was consistently overexpressed in clear-cell, endometrioid and high grade serous EOC subtypes

Comprehensive gene expression profiles of 40 EOC specimens of two different histological patterns (26 endometrioid and 14 clear-cell EOCs), and of their relative normal counterpart (15 normal endometrium) were generated using high-density oligonucleotide microarrays, with the aim to identify subtype-specific biomarkers.

A total of 1462 genes (468 up-regulated and 994 down-regulated) were found significantly expressed in 26 endometrioid EOCs when compared to 15 NEs (see Additional file 3: Table S4). When comparing 14 clear-cell EOCs to the same 15 NEs, 1479 differentially expressed genes (596 up-regulated and 883 down-regulated) were identified (see Additional file 4: Table S5).

FOXM1 was the second-most up-regulated gene in endometrioid EOCs compared to NEs (FC = 32.08, p_adj_ = 2.5E-5), and the 12^th^-most up-regulated gene in clear-cell EOCs compared to NEs (FC = 30.28, p_adj_ = 5.2E-05).

FOXM1 expression measured by genechip platform was highly correlated with FOXM1 expression measured on the same samples via confirmatory RT-qPCR: clear-cell EOCs vs NE (r_s_ = −0.743; p < 0.0001), endometrioid EOCs vs NE (r_s_ = −0.763; *p* < 0.0001). The negative values of the correlation coefficients come from the fact that higher Ct means lower expression.

**Fig. 1:**
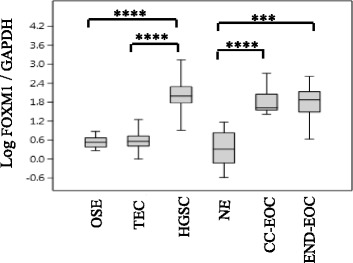
Differential expression of FOXM1 between EOC tissues and normal controls. FOXM1 expression was measured by RT-qPCR (log10) in all three histological variants and compared to OSE brushings, TEC brushings and NE. FOXM1 expression was normalized to GADPH. Student’s *t*-test, *, *P* ≤ 0.05, **, *p* ≤ 0.01, ***, *p* ≤ 0.001, ****, *p* ≤ 0.0001

FOXM1 overexpression was validated in clear-cell and endometrioid EOCs compared to NE by RT-qPCR (Fig. 1), then, it was evaluated also in 50 HGSCs, 10 TEC and 20 OSE brushings. Both type of comparison showed a significant up-regulation of FOXM1 in HGS subtype (Fig. 1).

Overall RT-qPCR analyses demonstrated that FOXM1 was overexpressed in different EOC histological subtypes compared to their appropriate normal controls (Fig. 1).

### FOXM1 protein was an independent prognostic marker for shorter disease specific survival in non-serous EOC patients and for increased risk of cancer progression in all platinum-resistant EOC patients

**Fig. 2:**
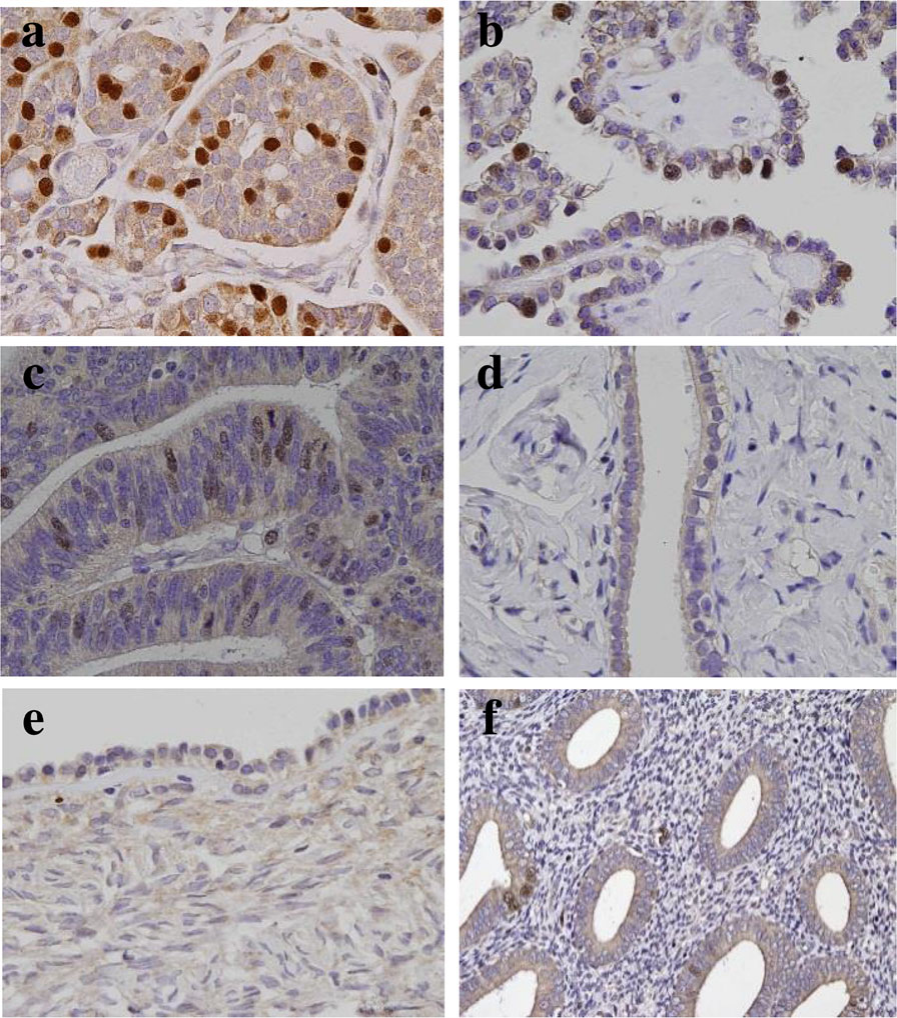
Validation of FOXM1 expression in EOC tissues of different subtype and normal controls by immunohistochemistry. Representative tumor sections of an HGSC (**a**), a clear-cell EOC (**b**) and an endometrioid EOC (**c**) stained for FOXM1 showing strong nuclear positive signal and weak cytoplasmic stain. Low FOXM1 immunoreactivity was detected in normal fallopian tube (**d**) and ovarian (**e**) epithelia, as well as endometrium (**f**), (40x magnifications)

We analyzed FOXM1 protein expression by IHC in 90 EOC tissue specimens and 10 in healthy tissues. FOXM1 was mainly expressed in the nucleus of primary EOCs regardless of their histological type, and marginally expressed in the cytoplasm, as depicted in Fig. 2a, b, c. Low immunostaining was detected in normal tubal and ovarian epithelia, as well as endometrium (Fig. 2d, e, f).

FOXM1 protein overexpression was significantly correlated to serous histology (*p* = 0.001), and advanced FIGO stage (*p* = 0.004), as shown in Table 2.

**Table 2:** Relationship between FOXM1 protein expression and clinicopathological variables of 90 patients considered for survival analyses

Characteristic	No.	Fc	P
Age		1.41	0.058
> 60	40		
≤ 60	50		
Histological type		1.76	0.001
Serous	50		
Non serous (14 clear-cell, 26 endometrioid)	40		
FIGO stage		1.71	0.004
III-IV	57		
I-II	33		
Grade		1.33	0.227
G3	73		
G1-2	17		
TR		1.32	0.126
> 0	46		
0	44		
Lymph node metastasis		1.15	0.526
Positive	23		
Negative	49		
Na	18		
Menopausal status		1.06	0.75
Pre	63		
Post	27		
Response to first line-chemotherapy		1.22	0.469
Non responder	12		
Responder	77		
Na	1		
Platinum sensitivity		0.98	0.923
Sensitive	56		
Partially sensitive-resistant	31		
Not treated	3		
Ascites		1.28	0.186
Yes	53		
No	36		
Na	1		
Cytology		1.33	0.138
Positive	56		
Negative	27		
Na	7		
CA125		1.04	0.683
1SD increase			

In order to study the association of FOXM1 protein expression with patients’ prognosis, FOXM1 IRS score was modelled as a binary (Low/High) factor and patients were categorized into the high and the low FOXM1 expression groups, respectively. Thresholds for FOXM1 categorization were computed both for DSS and PFS based on time-dependent ROC curves using 24 and 12 months, respectively, as time points that correspond approximately to 75% survival in each outcome. The estimated best thresholds for grouping FOXM1 expression as high versus low were 10.7 for DSS and 13.3 for PFS. DSS was regressed on FOXM1 expression groups via Cox regression with additional covariates being the FIGO stage, platinum resistance, and histotype dichotomized as serous versus non-serous. Given a significant departure from the proportional-hazards assumption, platinum response was entered as a stratification variable. Moreover, an interaction term for FOXM1 and histotype was entered in the model.

The effect of FOXM1 expression group on PFS was similarly modelled via Cox regression, with additional covariates again being the FIGO stage, platinum resistance and serous histotype. An interaction term was added for FOXM1 and platinum resistance.

**Fig. 3:**
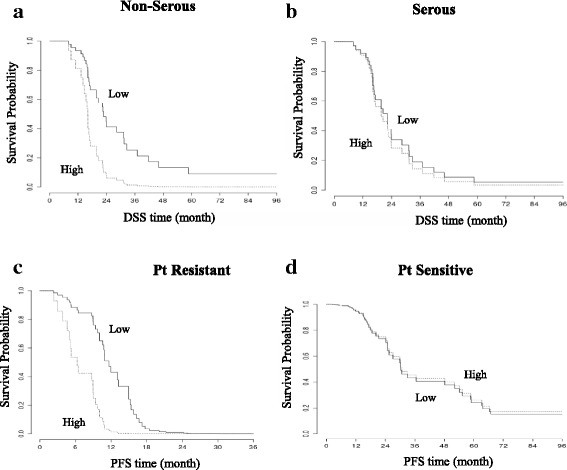
Kaplan-Meier disease specific survival (DSS) curves for EOC patients according to histology. **a** For non-serous patients, Kaplan-Meier plot of DSS shows a clear outcome difference between low and high FOXM1 expressing groups. **b** For serous patients, Kaplan–Meier plot of DSS shows the same survival probability between low and high FOXM1 expressing groups. Kaplan-Meier progression free survival (PFS) curves for EOC patients according to platinum response. **c** For Platinum (Pt)-resistant EOC patients, Kaplan-Meier plot shows a different risk of recurrence/progression between low and high FOXM1 expressing groups. **d** For Pt-sensitive patients, Kaplan–Meier plot does not show any outcome difference between low and high FOXM1 expressing groups

Considering DSS, the high level of FOXM1 was significantly associated with a worse prognosis (HR high vs low = 3.14, 95% CI = 1.04–9.43; *p*-value = 0.042) in non-serous histotypes (Fig. 3a). This association was not significant in serous samples, where it showed the same direction (HR high vs low = 1.17, 95% CI = 0.56–2.42; *p*-value = 0.67) (Fig. 3b).

Considering PFS, a high level of FOXM1 was significantly associated with a higher risk of disease recurrence/progression (HR high vs low = 5.07, 95% CI = 2.05–12.56; *p*-value < 0.001) in partially sensitive/resistant subjects (Fig. 3c), whereas the association was not significant in platinum sensitive subjects (HR high vs low = 0.94, 95% CI = 0.43–2.05; *p*-value = 0.88) (Fig. 3d).

### Histopathological features, genetic fingerprint, *BRCA* status and growth kinetic of new established OSPC2 and EOC-CC1 cell lines

We derived two new cell lines from clinical samples collected from either solid tumor (EOC-CC1) at first surgery or ascites (OSPC2) at disease progression, respectively. A detailed description of their immunocytochemical features, genetic fingerprint, *BRCA* status and growth rate analyses is reported in Additional file 5.

### FOXM1c was the isoform principally overexpressed in EOC-CC1 and OSPC2 cell lines and in matched tissue biopsies, and both FOXM1 c and b isoforms were successfully inhibited by siRNA transfection

**Fig. 4:**
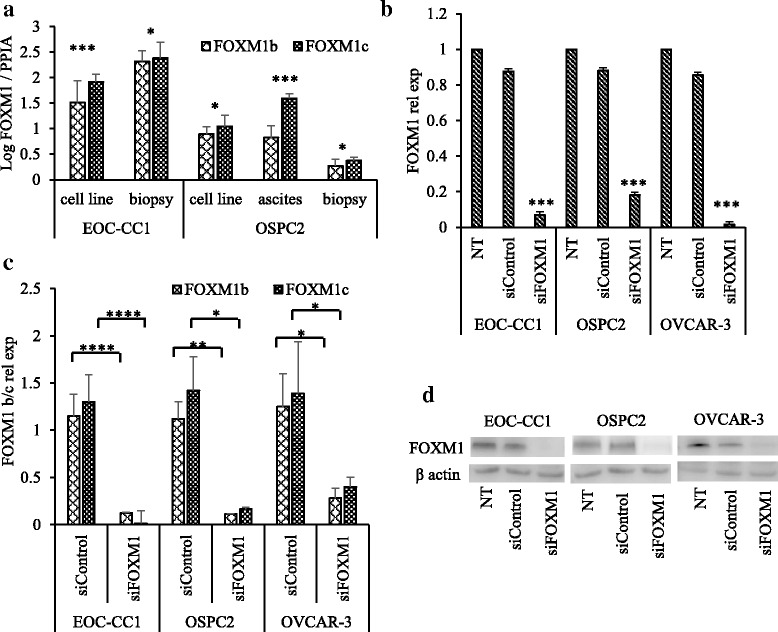
FOXM1 expression in EOC clinical samples and in derived cell lines. **a** Relative expression of FOXM1b and FOXM1c in paired EOC surgical/effusion samples and derived cell lines, as determined using RT-qPCR with isoform-specific primers and normalized against PPIA. **b** Expression of FOXM1 mRNA detected by RT-qPCR, normalized to GADPH in siFOXM1, siControl and untransfected (NT) cell lines. **c** Relative expression of FOXM1b and FOXM1c in primary cell lines after siRNA transfection, as determined using RT-qPCR with isoform-specific primers and normalized against PPIA. **d** Western blot analysis demonstrating the effectiveness of FOXM1 suppression in EOC cell lines. Data represent mean ± SD. Student’s *t*-test, *, *P* ≤ 0.05, **, *P* ≤ 0.01, ***, *P* ≤ 0.001, ****, *P* ≤ 0.0001

Since it is well known that alternative splicing of the Va (A1) and VIIa (A2) exons gives rise to three distinct FOXM1 variants, FOXM1a, FOXM1b, and FOXM1c [28], we first tested the expression of the two transcriptionally active isoforms, namely b and c, in EOC-CC1 and OSPC2 and in their respective primary EOC samples. FOXM1c was the predominant expressed isoform in all EOC tissue samples and in their derived cell lines (Fig. 4a). To determine the functional role of FOXM1 in EOC-CC1, OSPC2 and OVCAR-3 cell lines, its expression was transiently inhibited by siRNA. FOXM1 mRNA levels were significantly reduced in all EOC cell lines compared to control cells (scrambled siRNA), as detected by RT-qPCR (Fig. 4b). Following knockdown, we further confirmed the down-regulation of both FOXM1-b and -c isoforms by RT-qPCR (Fig. 4c). Western blot analysis demonstrated a reduced level of FOXM1 protein after 24 h transfection (Fig. 4d).

### Altered FOXM1 expression affected EOC cell proliferation, colony formation and invasion in vitro

**Fig. 5:**
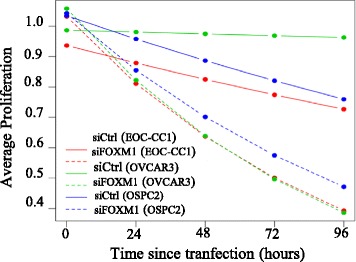
Cellular growth curves for EOC-CC1, OSPC2 and OVCAR-3 cell lines after siRNA transfection. Transient transfection of FOXM1-specific siRNA (siFOXM1) in EOC cell lines OVCAR-3, OSPC2, EOC-CC1, decreased their growth in culture for all time points in comparison to scrambled cells (siControl) (ANOVA test)

To determine the effect of reduced expression of FOXM1 on EOC biology, we knocked down overall expression of FOXM1 using a specific siRNA, as described above. Growth rate in culture was significantly reduced for all time points from 24 to 96 h in all cell lines (Fig. 5). Every 24 h, the proliferation in siFOXM1-EOC-CC1 cells was reduced by a factor of 0.84 compared to scrambled siRNA (siControl) cells (95% CI: 0.78–0.90; *p* < 0.001). Similarly, we estimated a FC = 0.78 (95% CI: 0.71–0.86; *p* < 0.001) and FC = 0.89 (95% CI: 0.82–0.96; *p* = 0.0011) for OVCAR-3 and OSPC2 cell lines, respectively (Fig. 5).

With flow cytometric analysis at 96 h culture after siRNA transfection, we found that FOXM1 knockdown did not induce any relevant change in cell-cycle profile of EOC cell lines, but slowed the growth kinetics of EOC cells in a cycle-specific fashion, as reported in Additional file 6: Figure S3.

**Fig. 6:**
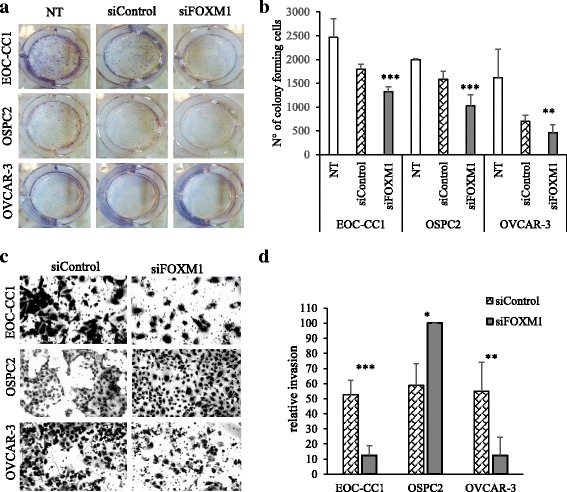
Targeting FOXM1 reduces colony formation capacity and alters invasion in EOC cells. **a** 24 h after siRNA transfection, EOC cells were plated in 6-well plate, and then colonies were stained and counted after incubation for 7–9 days. Results are representative of three independent experiments. **b** The graphs provide quantification of numbers of colony forming cells. Each column represents mean ± SD of quintuplicate determinations. One way ANOVA, **, *P* ≤ 0.01, ***, *P* ≤ 0.001. **c** Representative images showing invaded cells (matrigel-coated membrane) after 24 h. **d** Graphic representation of invasion results as fold change of invaded cells in 3 fields of triplicate wells; data show mean and SD from four independent experiments. *, *P* ≤ 0.05, **, *P* ≤ 0.01, ***, *P* ≤ 0.001, one-way ANOVA test

Clonogenic assays were performed 24 h after siRNA transfection, and cell numbers were measured 7–9 days later. A significantly reduced colony formation capacity was observed in the FOXM1-deleted populations in comparison to siControls in each EOC cell line (Fig. 6a, b).

*In vitro* transwell assays were used to study the effect of transient FOXM1 knockdown on EOC cells invasion. Significantly decreased invasion was observed in EOC-CC1 and OVCAR-3 cells that were FOXM1-silenced as compared to siControl cells, whereas OSPC2 cells showed an opposite behavior (Fig. 6c, d).

### FOXM1 knockdown rendered EOC-CC1 and OSPC2 cells more sensitive to chemotherapeutic agents in vitro

**Fig. 7:**
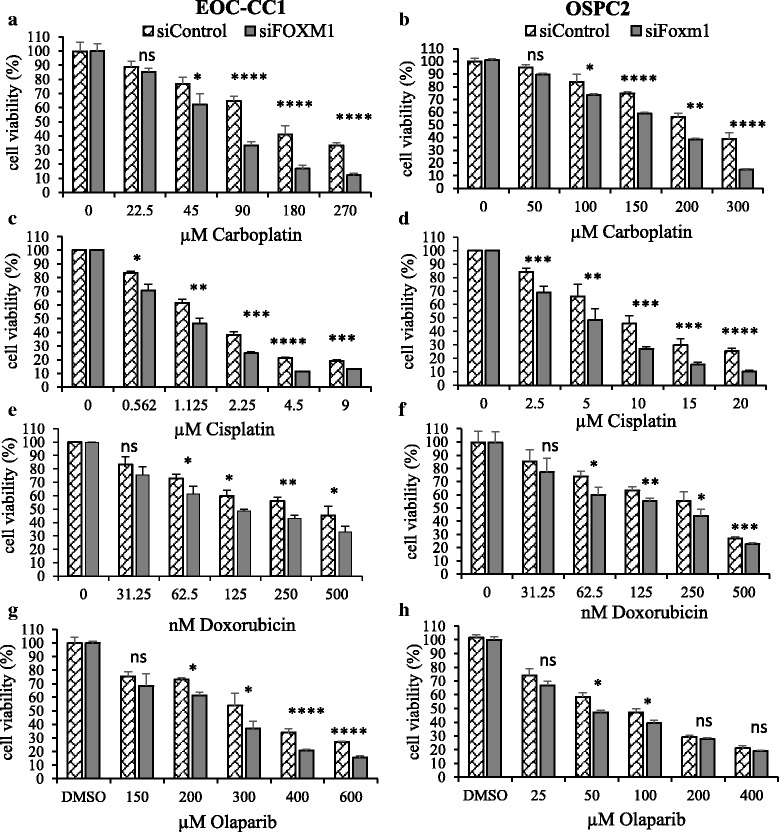
FOXM1 mediates resistance to drugs in EOC cells. EOC-CC1 **a, c, e, g** and OSPC2 **b, d, f, h** cell lines were transiently transfected with FOXM1-specific siRNA or scramble-siRNA for 24 h. After 48 h, cells were treated with increasing concentrations of the indicated drugs and proliferation rates were estimated by MTS assay. Results shown are representative of three independent experiments. Values were normalized to control cells and represent mean ± SD of quintuplicate determinations. Student’s *t* test was used for comparisons (*, *P* ≤ 0.05, **, *P* ≤ 0.01, ***, *P* ≤ 0.001, **** *P* ≤ 0.0001)

The role of FOXM1 in chemoresistance in our newly established EOC cell lines was examined 48 h after FOXM1 silencing with specific siRNA. EOC-CC1 and OSPC2 cells were treated with the indicated concentrations of drugs, as shown in Fig. 7. Cell viability was measured by MTS assay at 96 h post drug treatment. We evaluated the cytotoxic effect of: i) cisplatin and carboplatin; ii) doxorubicin hydrochloride; iii) olaparib. Interestingly, inhibition of FOXM1 increased sensitivity to both carboplatin (Fig. 7a-b) and cisplatin (Fig. 7c-d), and to doxorubicin hydrochloride (Fig. 7e-f) in both cell lines. Although viability of EOC cells decreased upon treatment with drugs in a dose-dependent manner, the increase in sensitivity to cisplatin, carboplatin and doxorubicin was independent of dose. Finally, FOXM1 inhibition significantly enhanced response to olaparib in EOC cells (Fig. 7g-h). We observed that the concentration of drugs that inhibited cell viability by 50% (the IC50) was lower in siFOXM1 than in siControl cells. The IC50 mean values for all drugs are shown in Table 3 as means ± SEM for three independent determinations.

**Table 3:** Estimated IC50 mean values ± SEM of drugs in primary EOC cell lines transfected with scramble-siRNA and FOXM1-specific siRNA

	IC50 values ± SEM					
	EOC-CC1			OSPC2		
Drug	siControl	siFOXM1		siControl	siFOXM1	
Cisplatin (μM)	1.9 ± 0.2	1.1 ± 0.1	*p* = 0.008	11 ± 0.4	5 ± 0.5	*p* < 0.001
Carboplatin (μM)	153 ± 15	57 ± 3	*p* = 0.003	236 ± 3	173 ± 2	*p* < 0.001
Doxorubicin (nM)	466 ± 17	119 ± 1	*p* < 0.001	270 ± 26	190 ± 3	*p* = 0.006
Olaparib (μM)	330 ± 7	233 ± 13	*p* = 0.05	101 ± 9	42 ± 2	*p* = 0.003

### Microarray analysis reveals the critical role of FOXM1 in the modulation of cell proliferation and in transcriptional regulation of genes involved in DNA repair, drug response and cancer metastasis

**Fig. 8:**
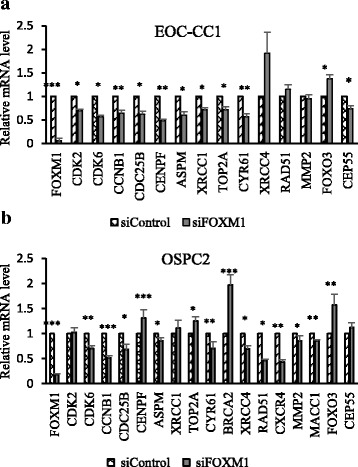
RT-qPCR validated genes in novel established EOC cell lines after FOXM1 inhibition. RT-qPCR analysis of FOXM1-target genes in siFOXM1 EOC-CC1 **a** and in siFOXM1 OSPC2 **b** cells compared to siControl cells, 24 h after transfection. Transcript expression levels were normalized against the geometric mean of SDHA and PPIA. Data represent mean ± SEM of three biological replicates. Mann Whitney test was used for comparisons (*, P ≤ 0.05, **, P ≤ 0.01)

To identify genes that were altered as a consequence of FOXM1 inhibition, we compared the gene expression profiles of siFOXM1 EOC cells to siControl ones, by microarray technology. Genes were selected according to their statistical significance (*P* < 0.001) and fold difference (2-fold) in each cell line (Additional file 7: Table S11, Table S12; Additional file 8: Table S13, Table S14; Additional file 9: Table S15, Table S16). When the fold change was averaged over all the three cell lines, 312 genes showed an average fold difference >2.0 and no additional filtering was applied on them (Additional file 10: Table S17). However, the individual fold changes of each cell line could differ significantly from the average fold change. In response to FOXM1 silencing, we found many up-regulated and very few under-expressed genes that behave homogeneously across cell lines. Interestingly, many of these genes are known to be involved in cell-cycle function (CDK2, CDK6, CCNB1, CEP55, CENPF), transcriptional regulation/tumor suppression (FOXO3, TOP2A), DNA-damage response (ASPM, XRCC4), and tumor progression (CYR61, MACC1, CXCR4). These genes were further investigated by RT-qPCR in EOC-CC1 and OSPC2 cell lines. Additionally, the expression levels of some known FOXM1 transcriptional targets involved in the cell cycle (CDC25B), invasion (MMP2), and DNA-repair mechanisms such as HR (BRCA2, RAD51) and base-excision repair (XRCC1) were assessed by RT-qPCR. Collectively, our findings showed that the EOC-CC1 and OSPC2 cell lines displayed a unique transcriptional profile in response to FOXM1 depletion (Fig. 8).

After FOXM1 knockdown, interactions among differentially expressed genes in new established EOC cell lines were investigated using Ingenuity Pathway Analysis (IPA) software. Functional analysis and annotation revealed a unique highly significant network in each cell line, respectively (Additional file 11: Figure S7). The main functions of the top genes in OSPC2 network were associated to sensitization to chemotherapy (CXCR4, ANKRD1, CYR61, EDN1, ATP1B1, ARHGEF1, RTF1), and tumor suppression (FGFR3, BCL11B). In EOC-CC1 network, down-regulation of FOXM1 elicited expression of several pro-inflammatory cytokines (CCL20, CXCL8), markers of subtype and chemoresistance (PTHLH, TFAM), immune response mediators (TLR5, IRAK2), and perturbed genes involved in tumor-stromal interactions (COL1A1), tumor vascularity (F2R, HMGB1, NFATC3) and hypercalcemia (PTHLH).

By enrichment analysis on a gene set of 111 invasion-related genes, we found that 4 genes (3.6%) were differentially expressed in siFOXM1 OSPC2 cells, namely, CXCR4, EPHB2, TIMP3, and TNFSF10. This constituted statistically significant enrichment compared to OSPC2’s background rate of 1.1% (387 out of 36.226) differential expression in genes unrelated to invasion (Fisher’s exact *P* = 0.032).

## Discussion

In the present study, we accurately selected two EOC patients’ cohorts with clear-cell and endometrioid histology, respectively, and we generated and compared their molecular signatures to normal endometrial tissue profiles, according to the new paradigm of ovarian cancer origin [3]. Our high throughput analyses revealed that FOXM1 gene was significantly up-regulated in both tumor subtypes. FOXM1 transcription factor is a potent oncogene involved in the onset and progression of multiple malignancies [10, 12], including HGSC [14–20], where its role as a marker of adverse prognosis and drug resistance has been extensively described [16, 17, 19]. On the contrary, its functions in less-common EOC subtypes, such as clear-cell and endometrioid, have never been investigated until now. Since false discovery is a concern when multiple events are tested in one sample, as in microarray experiments, we performed a validation study of FOXM1 expression through qRT-PCR considering all the three major EOC subtypes. We demonstrated for the first time the overexpression of FOXM1 in non-serous EOC compared to normal endometrium controls and in HGSC in comparison to either tubal epithelium or ovarian surface lining. When we examined the association of FOXM1 protein expression to clinicopathological features of patients belonging to the three subtype - specific cohorts, we found that FOXM1 level was significantly higher in serous than clear-cell and endometrioid EOCs, and correlated to advanced FIGO stage, regardless of tumor histotype. In addition, our survival analyses revealed that overexpression of FOXM1 was an independent predictor of increased risk of cancer progression in the subgroup of platinum-resistant patients, regardless of tumor histology and was an independent prognostic factor of worse disease specific survival in non-serous EOC, regardless of patient sensitivity to conventional platinum-based treatment. Overall, these findings suggest for the first time that FOXM1 is a reliable predictor of adverse outcome and a potential target to overcome drug-resistance even in non-serous EOCs.

Cell lines summarizing the characteristics of different histological and molecular EOC subtypes are a helpful resource to discover pathophysiology and to pre-clinically test anti-cancer drugs, but their extensive description is imperative for in vitro therapeutic studies. In view of our survival analyses suggesting FOXM1 involvement in patient outcome and drug resistance, we employed two new EOC cell lines established from FOXM1-expressing tumors, with serous (OSPC2) and clear-cell (EOC-CC1) histology, to evaluate and compare its functional role in two different EOC subtypes. First of all, we demonstrated that both newly established cell lines retained the histopathologic characteristics of the original clinical samples, thus representing a new platform to study the role of FOXM1 in a case of progressive HGSC, the most common and lethal form of EOC, and in a rarer clear-cell EOC. In agreement with FOXM1 isoforms’ expression in a panel of commercially available HGSC cell lines [34], we found that FOXM1c was the major isoform expressed in OSPC2 and EOC-CC1 cells and in the corresponding original clinical specimens. The suppression of both transcriptionally active FOXM1 isoforms b and c by FOXM1-specific siRNA transfection in our cellular models reduced the ability of EOC cells to proliferate as well as to form colonies, without inducing any relevant change in the proportion of cells in each cell cycle phase. Through these findings, the oncogenic role of FOXM1 was for the first time described in a clear-cell EOC and assessed in a metastatic HGSC cell line. When we examined the global transcriptional response of EOC cells to FOXM1 knockdown, we identified a large number of well-known cell cycle-related genes (CDC25B, CCNB1, CDK6, ASPM) in ovarian cancer [35–38], significantly down-regulated in both cell lines, coding for proteins structurally and functionally associated with the centromere-kinetochore complex, that were later validated by RT-qPCR. These results provided further evidence that loss of FOXM1 reduced EOC cells proliferation in a cell cycle-specific fashion.

In agreement with previously published studies [15–19], our experimental findings demonstrated that depletion of FOXM1 significantly attenuated the invasion ability of OVCAR-3 and EOC-CC1 cells, but unexpectedly potentiated the invasion ability of OSPC2 cells. This latter result was further strengthened by gene enrichment analysis on microarray data, which demonstrated siFOXM1 OSPC2 cells enriched by pro-invasive genes, namely EPHB2 [39] and TNFSF10 [40]. OSPC2 cell line was established from the ascites of an HGSC patient with disease progression after two cycle of carboplatin-paclitaxel therapy. The increased invasiveness of FOXM1-depleted OSPC2 cells might be ascribed to unique biological features, source patient genetics, or selection of subclones in culture. Another possible explanation may be that FOXM1 is a multifaceted player in cancer, and interacts with different signaling pathways [41] that may convey opposite effects according to which specific cellular model is involved.

A cell line-specific FOXM1-dependent gene-expression pattern was successfully validated by RT-qPCR for each cell line, respectively. Of interest, in FOXM1-depleted EOC-CC1 cells, we confirmed the down-regulation of cyclin-dependent kinase 2 (CDK2) gene, known as an ideal target to suppress in order to reduce the peritoneal spread of clear-cell EOC in xenograft mice and thereby to prolong their survival [42, 43]. Other genes specifically down-regulated in EOC-CC1 cells after FOXM1 inhibition were those coding for (i) the centrosomal protein CEP55, recently identified as an indicator of malignant conversion and progression in head-and-neck squamous cell carcinoma [44] and (ii) the DNA repair gene XRCC1, whose transcriptional regulation by FOXM1 has been demonstrated in osteosarcoma U2OS cells [45]. XRCC1 and CEP55 are supposed to be direct transcriptional targets of FOXM1 in serous EOC and head-and-neck squamous cell carcinoma, respectively [14, 44]. Moreover, in EOC-CC1 cells, we verified the transcriptional down-regulation of a structural protein of the kinetochore, named CENPF, that acts synergistically with FOXM1 to promote tumor growth and metastasis in prostate cancer and co-regulates other downstream targets and signaling pathways associated with prostate malignancy [46]. In addition to CENPF down-regulation, IPA analysis suggested many targets involved in tumor-stromal interactions and tumor vascularity by a complex regulatory network that might contribute to the effects of FOXM1 depletion in this cellular model. Finally, loss of FOXM1 in EOC-CC1 cells induced the down-regulation of the TOP2A gene, a marker of prognosis and response to therapy in platinum resistant/refractory EOCs [47].

The molecular profile of FOXM1-depleted OSPC2 cells revealed some common and other unique altered genes when compared to the profile of EOC-CC1. Despite the unexpected findings of invasion assay, we assessed by RT-qPCR that some FOXM1 downstream targets involved in metastasis, cancer progression, and cisplatin resistance, such as MMP2 [19] and MACC1 [48–50], were specifically down-regulated in OSPC2 cells after FOXM1 silencing. Down-regulation of the chemokine CXCR4 gene, known to be involved in cisplatin resistance [51], dissemination of peritoneal metastasis and development of cancer-initiating cells [52] in several cancer, including EOC, was another special feature of OSPC2 cells induced by FOXM1 loss. Moreover, IPA analysis generated connection between CXCR4 and NFkB, P38 MAPK, ERK1/2, CD3, TCR, RNA polymerase II, and estrogen receptor complexes.

Beyond altering tumor phenotype, we demonstrated that FOXM1 silencing conferred a greater sensitivity to standard first-line chemotherapeutic drugs, carboplatin and cisplatin, in our novel cellular models. Our data are consistent with those previously reported for commercially available HGSC cell lines [16, 18] and provide the first evidence of the efficacy of FOXM1 inhibition in overcoming platinum resistance in clear-cell EOC, a subtype that often displays a chemoresistant phenotype, which leads to poorer prognosis, especially in advanced-stage patients compared to HGSC ones [53]. Currently, no large-scale clinical study has identified a cytotoxic agent that is definitively effective against clear-cell EOCs, and the mechanism of resistance of these tumors to chemotherapy has not been well explained. Our molecular analyses showed the down-regulation of oncogene CYR61 [54] in FOXM1-depleted cells of both subtypes. Significant expression changes were also confirmed by RT-qPCR for some genes involved in homologous (HR) and non-homologous end joining (NHEJ) DNA damage-repair mechanisms in ovarian cancer, such as XRCC1 [55] in EOC-CC1, and XRCC4, RAD51 and BRCA2 [56] in OSPC2. Their molecular alteration might be also associated to the increased cellular response of FOXM1-depleted cells to second-line treatment drugs, such as doxorubicin [7] and olaparib [8, 9], clinically employed for relapsed EOC patients. Previously, the involvement of FOXM1 in resistance to doxorubicin has been reported in various carcinoma cell lines [57]. Our experimental findings suggested that the combined use of doxorubicin and FOXM1 inhibition might be an alternative strategy to enhance the therapeutic efficacy of PLD while, at the same time, reducing its dosage and limiting toxicity. In agreement, we also demonstrated a significant variation of the expression level of TOP2A gene whose protein product, topoisomerase II alpha, is the actual target poisoned by doxorubicin [47].

The efficacy of PARP inhibitors has been ascertained in HR-deficient breast and ovarian cancers as single agents or in combination with cytotoxic chemotherapy or ionizing radiation [58]. Essential components of HR are the tumor suppressor proteins *BRCA1* and *BRCA2*, since their absence results in unrepaired lesions, cell-cycle arrest and cell death in cancer cells exposed to cytotoxic chemotherapy [59]. However, *BRCA1/2* status is not the only useful biomarker to predict PARP-inhibitor effectiveness [60]. In the present study, we tested olaparib, the first PARP inhibitor approved for EOC [8], as a single agent, and found reduced cell viability at increasing drug concentrations, in both cellular models. The OSPC2 cell line was more sensitive than EOC-CC1 to olaparib, but they both showed a lower sensitivity if compared to published data derived from commercially available EOC cell lines [61]. Since our EOC cell lines did not harbor any deleterious mutations in *BRCA1/2* genes, it is likely that their responsiveness to this PARP inhibitor might be associated to other mild genetic, epigenetic or molecular HR defects, and we verified that FOXM1 inhibition enhanced olaparib cytotoxicity in both cell lines.

Finally, we demonstrated by RT-qPCR the significant overexpression of FOXO3 mRNA in FOXM1-depleted EOC cells of both subtypes. FOXO3, like FOXM1, is a forkhead transcription factor, a typical tumor suppressor and a functional antagonist of the oncogene FOXM1 [62]. Since FOXO3 competes for binding to the same FOXM1 transcriptional targets involved in the cell cycle regulation, in DNA-damage recognition and repair, and in cell survival, its overexpression could explain the increased sensitivity of FOXM1-depleted cells to chemotherapeutics. Targeting FOXM1 to increase FOXO3 expression may represent an innovative molecular therapeutic strategy to improve the efficacy of cytotoxic agents, especially in drug-resistant EOC.

## Conclusion

This is the first report proving evidence of a consistent overexpression of FOXM1 among the three major EOC subtypes, and demonstrating its prognostic value in non-serous EOC patients and in platinum-resistant cases, regardless of their histotype. A full description of the tumorigenic properties of FOXM1 has been reported for a clear-cell and a metastatic HGS EOC cell lines, accurately representing their tumors of origin. Importantly, our data suggest that FOXM1 might be considered a valid target for combinatorial anticancer therapy, despite molecular and histological tumor heterogeneity. Consistent with this view, FOXM1 small-molecule inhibitors [63] and other compounds that exert anticancer effects through suppression of the FOXM1-signaling cascade have been recently identified with the aim to develop new therapeutic strategies for treating chemotherapy-resistant EOC patients [64]. By IPA tool we identified new cell line-specific relationships among altered genes for future understanding of novel FOXM1-related mechanisms. Further investigations are needed to better comprehend the signaling cascade related to FOXM1 activation taking into account the diversity of EOC histotypes.

## Additional files

Additional file 1: Detailed protocols for the establishment and the characterization of the EOC cell lines. **Table S1:** Clinical features of patients and tumor characteristics of samples used to derive cell lines; **Table S2:** Antibodies features and detailed staining protocols; description of short tandem repeat (STR) DNA profiling; description of BRCA1/2 sequencing; determination of cell lines’ growth rate. (DOCX 23 kb)

Additional file 2: Description of microarray analysis on EOC tissue specimens and cell lines. **Table S3:** Genes related to tumor invasion-metastasis used for the Gene-Sets Enrichment Analysis (GSEA). (DOCX 18 kb)

Additional file 3: Table S4. Differentially expressed genes in endometrioid EOCs compared to normal endometrium. (XLSX 327 kb)

Additional file 4: Table S5. Differentially expressed genes in clear-cell EOCs compared to normal endometrium. (XLSX 337 kb)

Additional file 5: Results of the characterization of OSPC2 and EOC-CC1 cell lines; **Table S6:** Panel of immunocytochemical stains in EOC cell cultures; **Table S7:** STR profiles of EOC-CC1 and OSPC2 biopsies and derived cell lines; **Table S8:** List of *BRCA1* and *BRCA2* sequence variants in OSPC2 cell line; **Table S9:** List of *BRCA1* and *BRCA2* sequence variants in EOC-CC1 cell line; **Figure S1:** Immunohistochemical stain for FOXM1 in original clinical tumor samples and in derived cell lines. **Figure S2:** EOC cell lines growth curves; **Table S10:** Optimal cell densities for seeding different cell lines in culture. (DOCX 552 kb)

Additional file 6: Figure S3. Flow cytometric and BrdU analyses of DNA content in siFOXM1 EOC cells. (DOCX 1136 kb)

Additional file 7: **Figure S4:** Volcano plot displaying differential expressed genes between siFOXM1 and siControl EOC-CC1 cells. **Table S11:** List of down-regulated genes in siFOXM1 EOC-CC1 cells. **Table S12:** List of up-regulated genes in siFOXM1 EOC-CC1 cells. (DOCX 160 kb)

Additional file 8: **Figure S5:** Volcano plot displaying differential expressed genes between siFOXM1 and siControl OSPC2 cells. **Table S13:** List of down-regulated genes in siFOXM1 OSPC2 cells. **Table S14:** List of up-regulated genes in siFOXM1 OSPC2 cells. (DOCX 260 kb)

Additional file 9: **Figure S6:** Volcano plot displaying differential expressed genes between siFOXM1 and siControl OVCAR-3 cells. **Table S15:** List of down-regulated genes in siFOXM1 OVCAR-3 cells. **Table S16:** List of up-regulated genes in siFOXM1 OVCAR-3 cells. (DOCX 345 kb)

Additional file 10: Table S17. Differentially expressed genes in siFOXM1 EOC cells compared to siControl. (XLSX 100 kb)

Additional file 11: Figure S7. Functional analysis of the genome-wide transcriptional response in FOXM1-silenced EOC cells. Putative network reconstruction on EOC-CC1 and OSPC2 cells. (PDF 178 kb)

## Abbreviations

CC: Clear-cell
Ct: Comparative threshold cycle
DSS: Dspecific survival
END: Endometrioid
EOC: Epithelial ovarian cancer
FC: Fold Change
FDR: False Discovery Rate
FOXM1: Forkhead box M1
HGSC: High-grade serous carcinoma
HR: Homologous recombination
IC50: Half-maximal inhibitory concentration
IRS: Immunoreactive score
NE: Normal endometrium
NT: Untransfected
OSE: Ovarian surface epithelium
PARP: Poly-ADP-ribose polymerase
PFI: Platinum-free interval
PFS: Progression free survival
PLD: Pegylated liposomal doxorubicin hydrochloride
RT-qPCR: Quantitative reverse transcription PCR
siControl: scramble-siRNA transfected
siFOXM1: FOXM1-siRNA transfected
STR: Short Tandem Repeat
Td: Doubling time
TEC: Tubal epithelial cell.
