# Supplementary material for: FOXM1 expression is significantly associated with chemotherapy resistance and adverse prognosis in non-serous epithelial ovarian cancer patients
Source: J Exp Clin Cancer Res. 2017 May 8;36:63. doi: 10.1186/s13046-017-0536-y (PMC5422964; doi:10.1186/s13046-017-0536-y)
Supplement: Supplementary file 1 — Detailed protocols for the establishment and the characterization of the EOC cell lines. Table S1: Clinical features of patients and tumor characteristics of samples used to derive cell lines; Table S2: Antibodies features and detailed staining protocols; description of short tandem repeat (STR) DNA profiling; description of BRCA1/2 sequencing; determination of cell lines’ growth rate. (DOCX 23 kb) [file 13046_2017_536_MOESM1_ESM.docx]

**Additional File 1**

**Establishment and characterization of the EOC cell lines**

The OSPC2 cell line was established in permanent culture from the ascites of a patient affected by HGSC progressing after two cycles of carboplatin-containing regimen. Source-patient characteristics are described in Table S1. Ovarian ascites fluid was centrifuged and freshly isolated cells were plated in RPMI medium containing 10% FBS, 1% antibiotic and 0.3% antimycotic amphotericin B, in a humidified atmosphere of 95% air/5% CO2, at 37°C. The EOC-CC1 primary culture was obtained by enzymatic-mechanical disaggregation of a fresh ovarian biopsy into single-cell suspension from a chemotherapy-naïve clear-cell EOC patient at the time of the primary surgery [1]. Both cell lines were repetitively sub-cultured once to twice a week through 100 passage generations. The cells were maintained as a monolayer in RPMI-1640 medium supplemented with 10% FBS. OVCAR-3 (ATCC® HTB­161™), a model of progressive ovarian adenocarcinoma, was purchased from American Type Culture Collection and cultured under the same culture conditions. Cellular growth rates were determined as described below.

**Table S1** Clinical features of patients and tumor characteristics of samples used to derive cell lines

|  | **Patients derived cell lines** | |
| --- | --- | --- |
| **Clinical Parameter** | **EOC-CC1** | **OSPC2** |
| Age at diagnosis (y) | 50 | 43 |
| Tumor type | adenocarcinoma | adenocarcinoma |
| Histopathology sub-type | clear-cell | serous |
| Tumor grade | high | high |
| Disease stage | IV | IIIC |
| Ascites at surgery | yes | yes |
| CA125 (U/ml) at diagnosis | 1149 | 243 |
| Surgical debulking | sub-optimal | sub-optimal |
| Progression | yes | yes |
| Death | yes | yes |
| Cause of death | progression/pulmonary emphysema | progression |
| Overall survival (months) | 1 | 3 |
| First-line treatment | surgery | surgery, carboplatin/paclitaxel (2) |
| Previous personal history of cancer | no | no |
| Year of sampling | 2010 | 2005 |
| Chemotherapy naïve at sample collection | yes | no |

The epithelial nature, purity and the histological characteristics of EOC primary cell cultures were verified by immunocytochemical (ICC) staining on cytospin preparation, following standard procedures. ICC assays were performed using a panel of primary antibodies. Features and details of the staining protocols are reported in Table S2.

**Table S2** Antibodies features and details of staining protocols

| **Antibody** | **Type** | **Clone** | **Manifacturer** | **Dilution** | **Unmasking** | **Detection system** |
| --- | --- | --- | --- | --- | --- | --- |
| CKpan | Mouse | MNF116 | Dako | 1:100 | PH8.0 EDTA | EnVision |
| CLDN4 | Mouse | 3E2C1 | Invitrogen | 1:200 | PH8.0 EDTA | Novolink Polymer |
| Ber-EP4 | Mouse | Ber-EP4 | Leica | 1:100 | PH6.0 CITRATE | Novolink Polymer |
| WT-1 | Mouse | 6F-H2 | Dako | 1:50 | PH8.0 EDTA | Novolink Polymer |
| Vimentin | Mouse | V9 | Novocastra | 1:100 | not performed | EnVision |
| CD34 | Mouse | QBEND/10 | Leica | 1:200 | PH8.0 EDTA | Novolink Polymer |
| CK5/6 | Mouse | D5/16B4 | Invitrogen | 1:100 | PH8.0 EDTA | EnVision |
| CA125 | Mouse | Ov185:1 | Leica | \1:200 | PH8.0 EDTA | Novolink Polymer |
| EMA | Mouse | GP1.4 | Leica | 1:150 | PH8.0 EDTA | Novolink Polymer |
| p53 | Mouse | DO-7 | Thermo Scientific | 1:100 | PH8.0 EDTA | Novolink Polymer |
| Calretinin | Rabbit | Polyclonal | Invitogen | 1:200 | PH8.0 EDTA | EnVision |
| FOXM1 | Rabbit | Polyclonal | Santa Cruz biotechnology | 1:160 | PH6.0 CITRATE | Novolink Polymer |

**EOC cell lines authentication by short tandem repeat (STR) DNA profiling**

EOC cell lines were authenticated by short tandem repeat (STR) DNA profiling, as described below. Genomic DNA was isolated from EOC-CC1 and OSPC2 biopsies and cells harvested at the same passage (100th) used in *in vitro* experiments. STR profiling was perfomed using PowerPlex® Fusion System (Promega) according to the manufacturer's specifications. The amplification reaction was carried out on the thermal cycler Eppendorf™ Mastercycler™. Amplified fragments were detected by capillary electrophoresis Applied Biosystems 3130*xl* genetic analyzer. STR profiles were analyzed by GeneMapper 3.2.1 software.

**BRCA1/2 sequencing**

BRCA1 and BRCA2 mutation screening was performed using the BRCA MASTR assay v1.2 (Multiplicom, Niel, Belgium) and sequencing on MiSeq System Illumina (Illumina Inc. San Diego, California, United States) according to the manufacturer’s instructions, as described below and previously by Minucci et al. [2]. In particular starting from 50 ng of DNA, all coding regions of BRCA1 and BRCA2 were amplified using 93 amplicons divided over five separate Multiplex PCR amplification reaction. In a second step, a Universal PCR was performed to tag the amplicons with MIDs and adaptors using the Multiplicom MID Dx (Multiplicom) for Illumina MiSeq kit. The resulting tagged amplicons were mixed per sample by combining the different plexes, and subsequently purified using the Agencourt® AMPure® XP beads (Beckman Coulter Genomics, USA) and quantified by QuantiT_ PicoGreen_ dsDNA Reagent (LifeTechnologies). Next, the tagged amplicon libraries from different samples were pooled and further processed by bridge amplification followed by sequencing on MiSeq System Illumina (Illumina Inc. San Diego, California, United States) using the 2 × 250 bp paired-end sequencing chemistry and run on V2 sequencing flow cell according to the manufacturer’s protocols. The data analysis was performed with CE-IVD Amplicom Suite Software v.1.0.

**Growth rate analysis in novel established EOC cell lines**

Each cell line was seeded in three replicate wells per time point in 6-well plates. After 24, 48, 72, and 96 h culture, cells were counted on a hemocytometer chamber and assessed for viability via trypan blue exclusion. Doubling times (Td) were measured by the equation Td = ln 2/m, where m is the slope of linear fitting [3]. The highest number of cells showing continuing exponential growth after three days was selected for silencing and drug response MTT assays. For experiments in microscale, cells were seeded in 96-well plates at appropriate density in 200 μL medium and grown for 24 h before adding different amounts of the test compounds.

**REFERENCES**

1. Bellone S, Tassi R, Betti M, English D, Cocco E, Gasparrini S, et al. Mammaglobin B (SCGB2A1) is a novel tumour antigen highly differentially expressed in all major histological types of ovarian cancer: implications for ovarian cancer immunotherapy. Br J Cancer. 2013;109:462-71.
2. Minucci A, Scambia G, Santonocito C, Concolino P, Canu G, Mignone F, et al. Clinical impact on ovarian cancer patients of massive parallel sequencing for BRCA mutation detection: the experience at Gemelli hospital and a literature review. Expert Rev Mol Diagn. 2015;15:1383-403.
3. Kim DK. Estimating doubling time of cells in vitro. In Vitro Cell Dev Biol Anim. 1995 Jun;31(6):419-20.
