# Supplementary material for: FOXM1 expression is significantly associated with chemotherapy resistance and adverse prognosis in non-serous epithelial ovarian cancer patients
Source: J Exp Clin Cancer Res. 2017 May 8;36:63. doi: 10.1186/s13046-017-0536-y (PMC5422964; doi:10.1186/s13046-017-0536-y)
Supplement: Supplementary file 2 — Description of microarray analysis on EOC tissue specimens and cell lines. Table S3: Genes related to tumor invasion-metastasis used for the Gene-Sets Enrichment Analysis (GSEA). (DOCX 18 kb) [file 13046_2017_536_MOESM2_ESM.docx]

**Additional File 2. Microarray processing**

**Gene chip analysis on EOC tissue specimens**

Gene expression “signal” values obtained from Affymetrix Human HG-U133 plus 2.0 were computed via Affymetrix 5.0 software using the GeneChip 5.0 algorithm with arrays scaled to a median signal of 1500 [1], and transformed to their base-2 logarithms. Arrays were then normalized by standardizing them to their grand mean and average standard deviation. In order to select highly differentially expressed genes between tumor and normal groups, a non-statistical filtering was first applied. Genes were retained for analysis if the higher-expressing group had (a) an average normalized log2 (signal) of at least 6, and (b) 75% or more of its samples called Present by the GeneChip 5.0 algorithm. The comparison between tumor and normal samples was performed by means of Welch’s unequal-variance t-tests using SAS v9.4 software (The SAS Institute, Cary, NC, USA), and resulting p-values were adjusted for multiple comparisons using the false discovery rate (FDR) procedure [2]. Genes were considered of interest if the FDR q-value was 5% or smaller, and if the absolute value of the estimated fold change was 4.0 or greater.

**Genechip analysis on EOC cell lines**

Gene expression “gProcessedSignal” values were computed via Agilent’s Feature Extraction 11.5 software using standard default settings, and transformed to their base-2 logarithms. Arrays were then normalized by standardizing them to their grand mean and average standard deviation. In order to select highly differentially expressed “features” between siFOXM1 and siControl groups, a non-statistical filtering was first applied. Features were retained for analysis if at least one group had (a) an average adjusted log2 (signal) greater than 6, and (b) more than 50% scored as “well above background” by Agilent’s feature-extraction software, and the results were analyzed using SAS v9.3 software. Data for each feature were analyzed via two-way ANOVA with post-hoc analysis comparing siFOXM1-treated cultures to control-treated cultures within each cell line. Fold changes in feature expression between siFOXM1-treated and control-treated cultures were calculated as 2^|expression|, where |expression| is the absolute value of the adjusted log2-(signal) difference between groups. Features were considered noteworthy in volcano plots if they had both a fold change greater than 2 and an unadjusted p-value <0.001.

**Table S3** Genes related to tumor invasion/metastasis. List was previously compiled by SA biosciences (<http://www.sabiosciences.com/rt_pcr_product/HTML/PAHS-028Z.html>) and by Applied Biosystems (TaqMan® Array 96-well Human Tumor Metastasis Plate targets genes).

| **Gene symbols** |
| --- |
| APC, BRMS1, CASP8, CCL7, CD44, CD82, CDH1, CDH11, CDH6, CHD4, CDKN2A, CEACAM1, COL4A2, CST7, CTBP1, CTNNA1, CTSL, CTSK, CXCL12, CXCR2, CXCR4, DAPK1, DCC, DENR, EPHB2, ERBB2, ETV4, EWSR1, FAT, FGF2, FGFR4, FLT4, FN1, FXYD5, GNRH1, HGF, HPSE, HRAS, HTATIP2, IGF1, IL18, IL1B, IL1B1, ITGA7, ITGB3, KISS1, KISS1R, KRAS, LAMB1, LYPD3, MCAM, MET, MDM2, METAP2, MGAT5, MMP1, MMP2, MMP3, MMP7, MMP9, MMP10, MMP11, MMP13, MMP14, MTA1, MTA2, MTSS1, MYC, MYCL, NCAM1, NF2, NME1, NME4, NR4A3, PECAM1, PLAUR, PNN, PSCA, PTEN, PTGS2, RB1, RBL1, RBL2, RET, RHOC, RORB, RPSA, S100A4, SERPINB5, SERPINE1, SET, SMAD2, SMAD4, SNCG, SRC, SSTR2, SYK, TACSTD1, TCF20, TGFB1, TGFBR2, TIAM1, TIMP1, TIMP2, TIMP3, TIMP4, TMPRSS4, TNFSF10, TP53, TPBG, TRPM1, TSHR, TWIST1, VEGFA, VEGFC, WISP1 |

**REFERENCES**

1. Bellone S, Tassi R, Betti M, English D, Cocco E, Gasparrini S, et al. Mammaglobin B (SCGB2A1) is a novel tumour antigen highly differentially expressed in all major histological types of ovarian cancer: implications for ovarian cancer immunotherapy. Br J Cancer 2013;109:462–471.

2. BenjaminiY, and HochbergY. Controlling the false discovery rate: a practical and powerful approach to multiple testing. J. Roy. Stat. Soc. B. 1995;57:289–300.
