# Supplementary material for: FOXM1 expression is significantly associated with chemotherapy resistance and adverse prognosis in non-serous epithelial ovarian cancer patients
Source: J Exp Clin Cancer Res. 2017 May 8;36:63. doi: 10.1186/s13046-017-0536-y (PMC5422964; doi:10.1186/s13046-017-0536-y)
Supplement: Supplementary file 5 — Results of the characterization of OSPC2 and EOC-CC1 cell lines; Table S6: Panel of immunocytochemical stains in EOC cell cultures; Table S7: STR profiles of EOC-CC1 and OSPC2 biopsies and derived cell lines; Table S8: List of BRCA1 and BRCA2 sequence variants in OSPC2 cell line; Table S9: List of BRCA1 and BRCA2 sequence variants in EOC-CC1 cell line; Figure S1: Immunohistochemical stain for FOXM1 in original clinical tumor samples and in derived cell lines. Figure S2: EOC cell lines growth curves; Table S10: Optimal cell densities for seeding different cell lines in culture. (DOCX 552 kb) [file 13046_2017_536_MOESM5_ESM.docx]

**Additional File 5**

**Characterization of OSPC2 and EOC-CC1 cell lines**

The epithelial phenotype of both EOC cell lines, denoted by a monolayer, was assessed by ICC staining for epithelial, mesothelial, endothelial and mesenchymal markers, whose expression pattern is reported in Table S6. Both EOC cell lines were positive for pan cytokeratin, cytokeratin 5/6, EMA, p53, CA125, Ber-EP4 and claudin 4, and negative for CD34 and calretinin, confirming the epithelial phenotype of the cultures. The EOC-CC1 cell line was characterized by cells of heterogeneous sizes, some displaying abundant clear cytoplasm and eccentric hyperchromatic nuclei, typical features of clear-cell histology, whereas the OSPC2 cell line developed into predominantly spindle cells that grew in sheets of regular clusters. Fibroblast-shaped cells were completely absent. Nuclear expression of FOXM1 was confirmed by staining with FOXM1-specific antibody on cancer cells of the original OSPC2 specimen (ascites’ cytospin) (Figure S1A) and in derived cell line (Figure S1B), both showing a focal strong positivity. For EOC-CC1, a diffuse medium-to-strong positive nuclear stain for FOXM1 was detected in FFPE ovarian tissue (Figure S1C), as well as in cell culture (Figure S1D).

| **Table S6 Panel of immunocytochemical stains in primary EOC cell cultures**   \|  \| **Cell lines** \| \|  \| **FFPE EOC biopsies** \| \| \| --- \| --- \| --- \| --- \| --- \| --- \| \|  \| **EOC-CC1** \| **OSPC2** \|  \| **EOC-CC1** \| **OSPC2** \| \| **CKpan** \| positive \| positive \|  \| positive \| positive \| \| **CLDN4** \| positive \| positive \|  \| positive \| na \| \| **Ber-EP4** \| positive \| positive \|  \| na \| positive \| \| **WT-1** \| positive \| negative \|  \| na \| negative \| \| **Vimentin** \| positive \| negative \|  \| na \| na \| \| **CD34** \| negative \| negative \|  \| na \| negative \| \| **CK5/6** \| positive \| positive \|  \| na \| negative \| \| **CA125** \| positive \| positive \|  \| positive \| positive \| \| **EMA** \| positive \| positive \|  \| na \| na \| \| **p53** \| positive \| positive \|  \| positive \| positive \| \| **Calretinin** \| negative \| negative \|  \| na \| negative \| \| **FOXM1** \| positive \| positive \|  \| positive \| positive \|   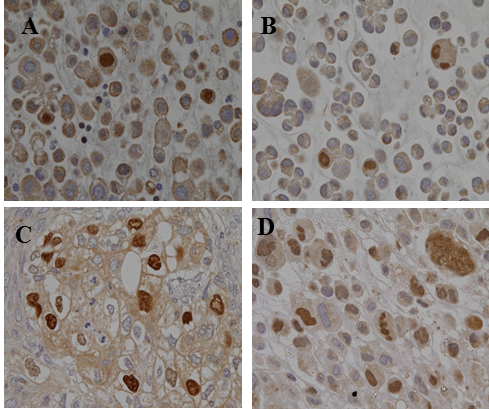 |  |  |
| --- | --- | --- | --- | --- | --- | --- | --- | --- | --- | --- | --- | --- | --- | --- | --- | --- | --- | --- | --- | --- | --- | --- | --- | --- | --- | --- | --- | --- | --- | --- | --- | --- | --- | --- | --- | --- | --- | --- | --- | --- | --- | --- | --- | --- | --- | --- | --- | --- | --- | --- | --- | --- | --- | --- | --- | --- | --- | --- | --- | --- | --- | --- | --- | --- | --- | --- | --- | --- | --- | --- | --- | --- | --- | --- | --- | --- | --- | --- | --- | --- | --- | --- | --- | --- | --- | --- |

**Figure S1** Immunocytochemical staining for FOXM1. Focal strong nuclear expression of FOXM1 was identified in the original OSPC2 specimen (ascites’ cytospin) (**A**) and in derived cell line (**B**). For EOC-CC1, a diffuse medium-to-strong positive nuclear signal for FOXM1 was detected in FFPE ovarian tissue (**C**), as well as in cell culture (**D**).

**Short tandem repeat (STR) analysis**

The unique identity of each cell line was confirmed by STR analysis. Moreover, a perfect concordance between STR profile of EOC-CC1 and OSPC2 patients’ biopsies and the derived cell lines was found. Comparison of DNA fingerprinting results in DSMZ database confirmed unique identity of each cell line. Table S7 depicts the complete STR profile for EOC-CC1 and OSPC2 patients’ biopsies and derived cell lines. A perfect concordance between STR profiles of tumor biopsies and their derived cell lines was found. Amelogenin marker is present, confirming that EOC-CC1 and OSPC2 are cells from females.

**Table S7 STR profiles of EOC-CC1 and OSPC2 biopsies and derived cell lines.**

| **Markers** | **EOC-CC1 bx** | **EOC-CC1 cell line** | **OSPC2 bx** | **OSPC2 cell line** |
| --- | --- | --- | --- | --- |
| **AMEL** | X | X | X | X |
| **D3S1358** | 17-18 | 17 | 14-16 | 14-16 |
| **D1S1656** | 12-15 | 12-15 | 12-14 | 12-14 |
| **D2S441** | 11-14 | 11-14 | 11 | 11 |
| **D10S1248** | 14-15 | 14 | 14-16 | 14 |
| **D13S317** | 11 | 11 | 11-12 | 11-12 |
| **Penta E** | 11-14 | 11-14 | 13-17 | 13-17 |
| **D16S539** | 11-12 | 11 | 9-13 | 9-13 |
| **D18S51** | 14-15 | 14-(15) | 16-20 | 16-20 |
| **D2S1338** | 23-26 | 26 | 17-24 | 17-24 |
| **CSF1PO** | 9-12 | 9-12 | 11-12 | 11-12 |
| **Penta D** | 10 | 10 | 9-12 | 9-12 |
| **TH01** | 6-7 | 6 | 6-9.3 | 6-9.3 |
| **vWA** | 14-16 | 14-16 | 14-18 | 14-18 |
| **D21S11** | 32.2-33.2 | 32.2-33.2 | 30-30.3 | 30-30.3 |
| **D7S820** | 8-12 | 8 | 10-12 | 10 |
| **D5S818** | 10-13 | 10-13 | 10-13 | 10-13 |
| **TPOX** | 9-10 | 9-10 | 8-11 | 8-11 |
| **DYS391** | - | - | - | - |
| **D8S1179** | 13-14 | 13 | 13-15 | 13-15 |
| **D12S391** | 20-22 | 20-22 | 20-23 | 20-23 |
| **D19S433** | 14-16 | 14-16 | 12-15 | 12-15 |
| **FGA** | 20-24 | 20 | 19-21 | 19-21 |
| **D22S1045** | 15-16 | 15-16 | 16 | 16 |

**BRCA1/2 Mutation Screening**

As described by the ClinVar database (http://www.ncbi.nlm.nih.gov/clinvar/), all detected variants were classified as polymorphisms with no pathogenetic significance, except for one intronic variant in the BRCA2 gene (c.1909+22_1909+22 delT). Conflicting interpretations are reported for this variant, so its clinical significance remains uncertain (Class 3). In OPSC2 cell line, four missense variations, three synonymous variations and seven internal variant sequence (IVS) in BRCA1 and five synonymous variations, one IVS and one untranslated region (UTR) variation in BRCA2 were found (Table S8). In EOC-CC1 cell line, one missense variation and one IVS in BRCA1, and five synonymous variations, three IVS and two UTR variations in BRCA2 were found (Table S9). As described by the ClinVar database (http://www.ncbi.nlm.nih.gov/clinvar/), all variants are classified as polymorphism with any pathogenetic significance, except for one intronic variant in BRCA2 gene (c.1909+22_1909+22 delT), found in both cell lines, for which there are conflicting interpretation about the clinical classification.

**Table S8 BRCA1 and BRCA2 sequence variants in OSPC2 cell line**

**______________________________________________________________________________**

**Exon Variants Mutation ClinVar**

**type Cassification**

______________________________________________________________________________

**BRCA1**

2 c. -18-115 T>C IVS hom B

9 c.548-58_548-58 delT IVS het B

11 c.3113 A>G (p.Glu1038Gly) M het B

11 c.2082 C>T (p.Ser694Ser) Syn hom B

11 c.3548 A>G (p.Lys1183Arg) M hom B

11 c.2311 T>C (p.Leu771Leu) Syn het B

11 c.2612 C>T (p.Pro871Leu) M het B

11 c.4097-141 A>C IVS hom B

13 c.4308 T>C (p.Ser1436Ser) Syn het B

14 c.4485-63 C>G IVS hom B

17 c.4837 A>G (p.Ser1613Gly) M het B

17 c.4987-92 A>G IVS het B

17 c.4987-68 A>G IVS het B

18 c.5152+66 G>A IVS hom B

_____________________________________________________________________________

**BRCA2**

10 c1909+22_1909+22 delT IVS hom CI

11 c.3396A>G (p.Lys1132Lys) Syn het B

11 c.3807 T>C (p.Val1269Val) Syn het B

11 c.4563 A>G (p.Leu1521Leu) Syn het B

11 c.6513 G>C (p.Val2171Val) Syn het B

14 c.7397T>C (Ala2466Ala) Syn het B

27 c10362 A>C 3’UTR hom B

_____________________________________________________________________________

B, benign; CI, conflicting interpretation; het, heterozygous; hom, homozygous; IVS, internal

variant sequence; M, missense; Syn, synonymous; UTR, untranslated region.

**Table S9 BRCA1 and BRCA2 sequence variants in EOC-CC1 cell line**

**______________________________________________________________________________**

**Exon Variants Mutation ClinVar**

**type Cassification**

______________________________________________________________________________

**BRCA1**

8 c.442-34 C>T IVS hom B

11 c.1067 A>G (pGln356Arg) M het B

______________________________________________________________________________

**BRCA2**

2 c.-26 G>A 5’UTR het B

7 c.631+183 T>A IVS hom B

10 c.1909+22_1909+22 delT IVS hom CI

11 c.3396 A>G (pLys1132Lys) Syn het B

11 c.3807 T>C (pVal1269Val) Syn het B

11 c.4563 A>G (pLeu1521Leu) Syn het B

11 c.6513 G>C (pVal2171Val) Syn het B

11 c.6841+80_6841+83 delTTAA IVS hom B

14 c.7397T>C (Ala2466Ala) Syn het B

27 c.10362 A>C 3’UTR hom B

______________________________________________________________________________

B, benign; CI, conflicting interpretation; het, heterozygous; hom, homozygous; IVS, internal variant sequence; M, missense; Syn, synonymous; UTR, untranslated region.

**Growth curves of cell lines**

Figure S2 depicts the cellular-growth curves (log) for each EOC cell line used in this study. It is possible to appreciate that after the first 48 h of incubation, the cellular growth begun in an exponential fashion in all cell lines (Figure S2). Seeding densities per well per cell line were identified as the optimal dynamic range in order to maintain a constant doubling time (Table S10).

**Figure S2** Proliferation rate of EOC cell lines. Cell counts were determined using a Neubauer chamber in the presence of trypan blue. All measures were perfomed in triplicate. A representative growth curve is shown for each cell line.

**Table S10 Optimal cell densities for seeding different cell lines**

| **EOC cell line** | **doubling time (hrs)** | **cell number per well (6 -well)** |
| --- | --- | --- |
|  |  |  |
| EOC-CC1 | 22 | 3-6 x 10^4^ |
| OSPC2 | 21 | 4-12 x 10^4^ |
| OVCAR-3 | 17 | 2-6 x 10^4^ |
|  |  |  |
