# Supplementary material for: FOXM1 expression is significantly associated with chemotherapy resistance and adverse prognosis in non-serous epithelial ovarian cancer patients
Source: J Exp Clin Cancer Res. 2017 May 8;36:63. doi: 10.1186/s13046-017-0536-y (PMC5422964; doi:10.1186/s13046-017-0536-y)

**Additional File 6**

**Flow cytometry and BrdU analysis results**

With flow cytometric analysis at 96 h culture after siRNA transfection, we found that FOXM1 knockdown didn’t induce any relevant change in cell-cycle profile of EOC cell lines (Figure S3 A, D, G), as shown by relative cell percentages reported inside the DNA histograms depicted in Fig S3 B, E, H. Si-FOXM1 treatments induced mild cell-cycle delay in G2/M (Figure S3 B, E, H), without significant effects on cell death. At previous time points, percent of cells in different phases of the cell cycle did not change considerably. For a more accurate cell-cycle analysis, EOC cells were treated with FOXM1-specific or scrambled siRNA up to 72 h, exposed briefly to BrdU and analyzed by flow cytometry. As a consequence of FOXM1 depletion, a weak effect on DNA synthesis was observed by BrdU analysis (Figure S3 C, F, I). A slight decrease in BrdU incorporation and a modest increase of the amount of G2/M cells were observed at 72 h in FOXM1-silenced cells. In conclusion, FOXM1 down-regulation significantly affected cell proliferation but it did not cause any specific cell-cycle phase delay in EOC cells. Accordingly, inhibition of FOXM1 did not induce apoptosis. Inhibition of FOXM1 expression slowed the growth kinetics of EOC cells in a cycle-specific fashion.


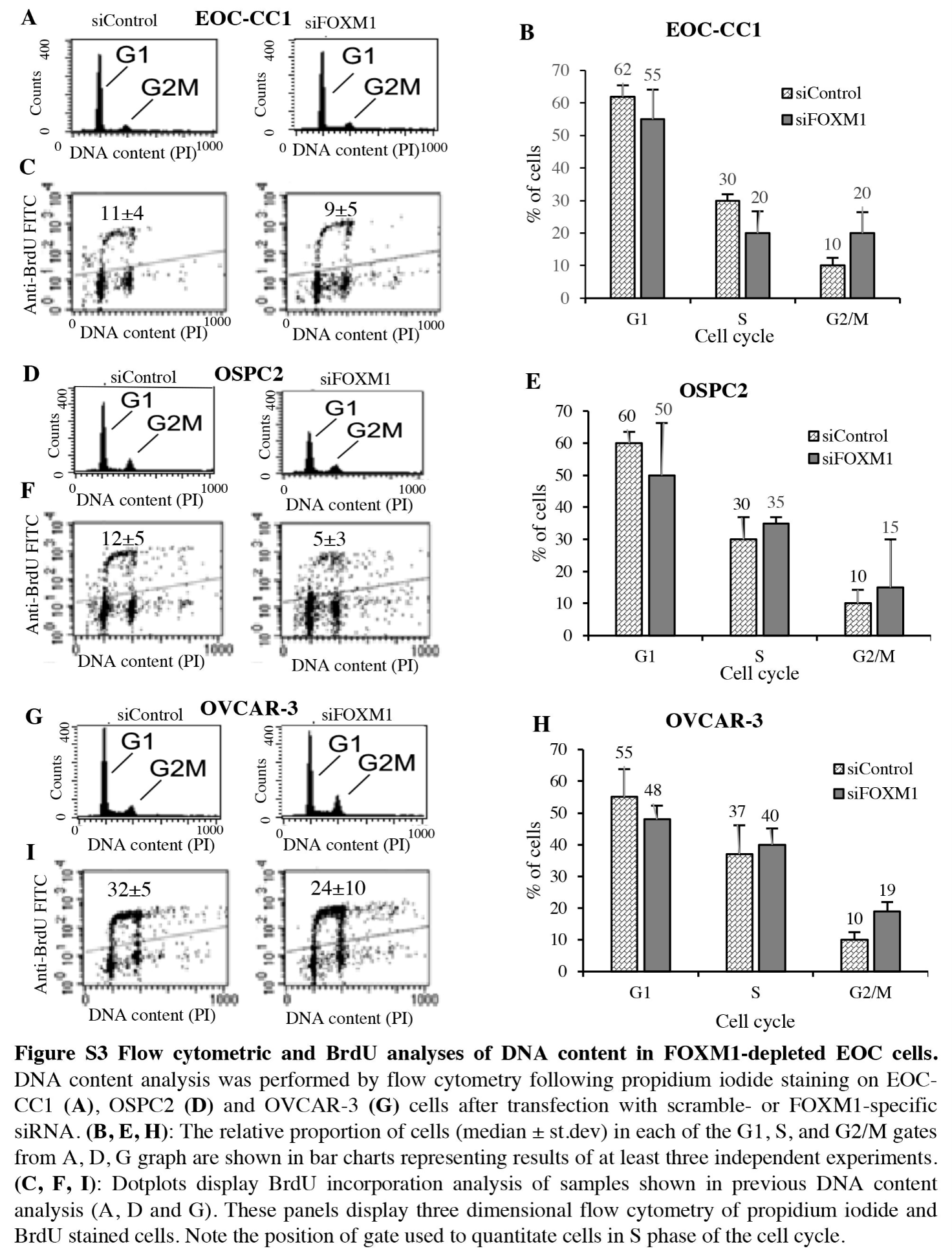

Supplement: Supplementary file 6 — Flow cytometric and BrdU analyses of DNA content in siFOXM1 EOC cells. (DOCX 1136 kb) [file 13046_2017_536_MOESM6_ESM.docx]
