# Supplementary material for: FOXM1 expression is significantly associated with chemotherapy resistance and adverse prognosis in non-serous epithelial ovarian cancer patients
Source: J Exp Clin Cancer Res. 2017 May 8;36:63. doi: 10.1186/s13046-017-0536-y (PMC5422964; doi:10.1186/s13046-017-0536-y)
Supplement: Supplementary file 7 — Figure S4: Volcano plot displaying differential expressed genes between siFOXM1 and siControl EOC-CC1 cells. Table S11: List of down-regulated genes in siFOXM1 EOC-CC1 cells. Table S12: List of up-regulated genes in siFOXM1 EOC-CC1 cells. (DOCX 160 kb) [file 13046_2017_536_MOESM7_ESM.docx]

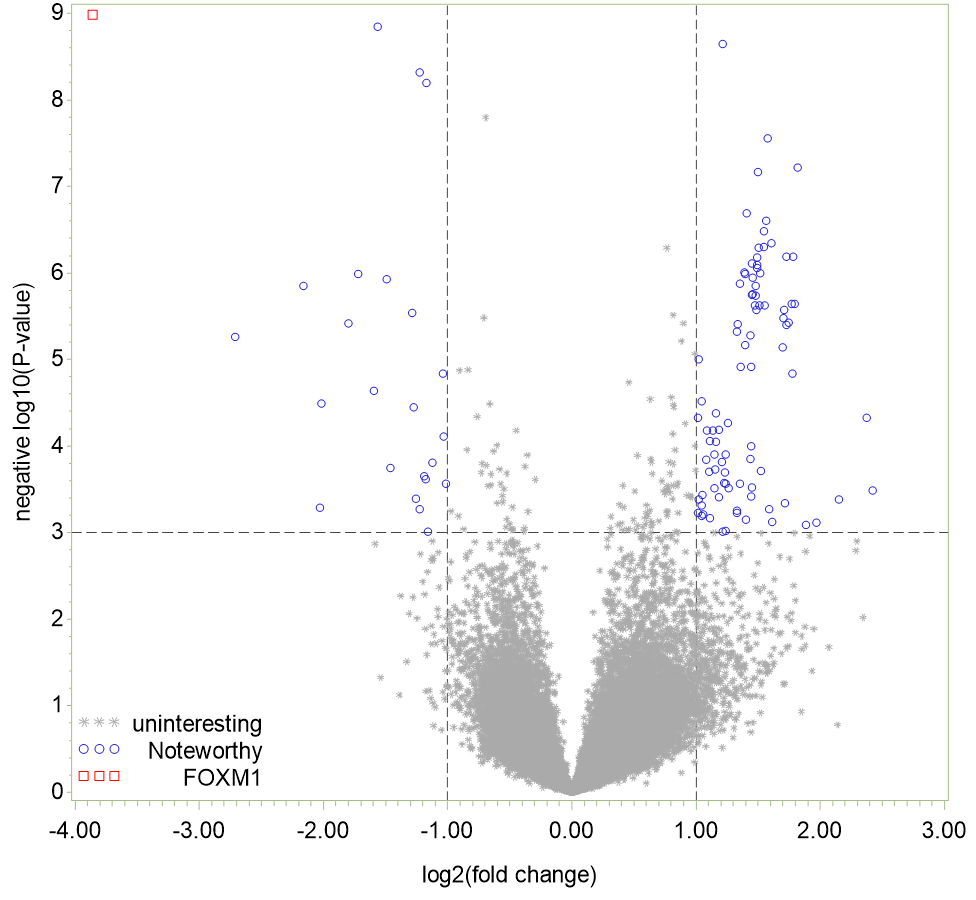


**Figure S4**. Volcano plot displaying differential expressed genes between siFOXM1 and siControl EOC-CC1 cells. The horizontal axis shows the base-2 logarithms of gene-expression fold changes for siFOXM1 cells compared to siControl cells. Vertical reference lines at +1.00 and –1.00 denote expression changes of 2 folds up and 2 folds down, respectively; data points outside these two vertical reference lines denote genes that show a >2-fold change in expression. The vertical axis shows the negative of the base-10 logarithm of the unadjusted P-values associated with expression changes. The horizontal line at 3 denotes unadjusted P=0.001; data points above this horizontal reference line thus have P<0.001 associated with their expression change. The red square denotes FOXM1. Blue circles denote non-FOXM1 genes that meet the double requirement of >2-fold change and P<0.001, whereas gray asterisks denote genes that do not meet the double requirement.

| **FeatureNum** | **GeneName** | **Estimate** | **Standard Error** | **DF** | **Pr > \|t\|** | **False Discovery Rate p-value** | **Fold Change** | **Direction of Change** |
| --- | --- | --- | --- | --- | --- | --- | --- | --- |
| 55112 | FOXM1 | -3.860 | 0.229 | 12 | <.0001 | <.0001 | 14.52 | Down |
| 55714 | SRD5A1 | -2.710 | 0.352 | 12 | <.0001 | 0.0024 | 6.54 | Down |
| 59943 | SRD5A1 | -2.160 | 0.246 | 12 | <.0001 | 0.0010 | 4.47 | Down |
| 48478 | NEURL1B | -2.026 | 0.431 | 12 | 0.0005 | 0.0697 | 4.07 | Down |
| 46129 | COL1A1 | -2.014 | 0.313 | 12 | <.0001 | 0.0104 | 4.04 | Down |
| 37932 | IRAK2 | -1.802 | 0.226 | 12 | <.0001 | 0.0018 | 3.49 | Down |
| 23244 | DDAH1 | -1.720 | 0.190 | 12 | <.0001 | 0.0009 | 3.29 | Down |
| 12743 | TROAP | -1.594 | 0.239 | 12 | <.0001 | 0.0079 | 3.02 | Down |
| 3349 | FAM96A | -1.565 | 0.096 | 12 | <.0001 | <.0001 | 2.96 | Down |
| 59536 | DNAJB6 | -1.491 | 0.167 | 12 | <.0001 | 0.0010 | 2.81 | Down |
| 7563 | PPP1R3F | -1.459 | 0.274 | 12 | 0.0002 | 0.0370 | 2.75 | Down |
| 46839 | DNAJB6 | -1.287 | 0.157 | 12 | <.0001 | 0.0016 | 2.44 | Down |
| 59416 | NDE1 | -1.273 | 0.200 | 12 | <.0001 | 0.0110 | 2.42 | Down |
| 20403 | TMEM185B | -1.253 | 0.259 | 12 | 0.0004 | 0.0585 | 2.38 | Down |
| 5044 | APOO | -1.225 | 0.083 | 12 | <.0001 | <.0001 | 2.34 | Down |
| 2834 | TMEM185B | -1.225 | 0.262 | 12 | 0.0005 | 0.0715 | 2.34 | Down |
| 6390 | MAX | -1.192 | 0.229 | 12 | 0.0002 | 0.0412 | 2.29 | Down |
| 46811 | TMEM185B | -1.180 | 0.229 | 12 | 0.0002 | 0.0441 | 2.27 | Down |
| 59118 | BAMBI | -1.170 | 0.081 | 12 | <.0001 | <.0001 | 2.25 | Down |
| 28534 | TMEM185B | -1.162 | 0.268 | 12 | 0.0010 | 0.1008 | 2.24 | Down |
| 35494 | TMEM185B | -1.120 | 0.207 | 12 | 0.0002 | 0.0333 | 2.17 | Down |
| 53547 | PTHLH | -1.038 | 0.148 | 12 | <.0001 | 0.0051 | 2.05 | Down |
| 12384 | STMN3 | -1.034 | 0.177 | 12 | <.0001 | 0.0205 | 2.05 | Down |
| 21708 | TRIB1 | -1.017 | 0.200 | 12 | 0.0003 | 0.0475 | 2.02 | Down |

| **FeatureNum** | **GeneName** | **Estimate** | **Standard Error** | **DF** | **Pr > \|t\|** | **False Discovery Rate p-value** | **Fold Change** | **Direction of Change** |
| --- | --- | --- | --- | --- | --- | --- | --- | --- |
| 34220 | PANK3 | 2.422 | 0.487 | 12 | 0.0003 | 0.0509 | 5.36 | UP |
| 38318 | SAR1B | 2.374 | 0.384 | 12 | <.0001 | 0.0136 | 5.18 | UP |
| 265 | MYB | 2.149 | 0.446 | 12 | 0.0004 | 0.0595 | 4.44 | UP |
| 3555 | ACPL2 | 1.971 | 0.441 | 12 | 0.0008 | 0.0884 | 3.92 | UP |
| 34388 | RUNX2 | 1.885 | 0.426 | 12 | 0.0008 | 0.0923 | 3.69 | UP |
| 11845 | EMP2 | 1.816 | 0.154 | 12 | <.0001 | 0.0002 | 3.52 | UP |
| 52756 | THG1L | 1.797 | 0.214 | 12 | <.0001 | 0.0014 | 3.48 | UP |
| 19944 | THG1L | 1.780 | 0.188 | 12 | <.0001 | 0.0008 | 3.43 | UP |
| 18854 | THG1L | 1.777 | 0.254 | 12 | <.0001 | 0.0051 | 3.43 | UP |
| 34233 | THG1L | 1.770 | 0.211 | 12 | <.0001 | 0.0014 | 3.41 | UP |
| 14268 | THG1L | 1.745 | 0.218 | 12 | <.0001 | 0.0018 | 3.35 | UP |
| 49039 | THG1L | 1.730 | 0.183 | 12 | <.0001 | 0.0008 | 3.32 | UP |
| 23916 | THG1L | 1.729 | 0.218 | 12 | <.0001 | 0.0018 | 3.31 | UP |
| 21457 | A_24_P280897 | 1.715 | 0.359 | 12 | 0.0005 | 0.0637 | 3.28 | UP |
| 30115 | THG1L | 1.707 | 0.207 | 12 | <.0001 | 0.0015 | 3.27 | UP |
| 30626 | THG1L | 1.707 | 0.211 | 12 | <.0001 | 0.0017 | 3.26 | UP |
| 55288 | THG1L | 1.696 | 0.226 | 12 | <.0001 | 0.0029 | 3.24 | UP |
| 28108 | TMEM50B | 1.615 | 0.360 | 12 | 0.0007 | 0.0873 | 3.06 | UP |
| 16347 | STEAP3 | 1.607 | 0.164 | 12 | <.0001 | 0.0007 | 3.05 | UP |
| 6012 | IL17RD | 1.591 | 0.340 | 12 | 0.0005 | 0.0715 | 3.01 | UP |
| 28677 | A_33_P321056 | 1.579 | 0.125 | 12 | <.0001 | <.0001 | 2.99 | UP |
| 48676 | YWHAH | 1.563 | 0.151 | 12 | <.0001 | 0.0005 | 2.95 | UP |
| 43741 | CHST15 | 1.553 | 0.186 | 12 | <.0001 | 0.0014 | 2.93 | UP |
| 44058 | POLR3E | 1.549 | 0.154 | 12 | <.0001 | 0.0006 | 2.93 | UP |
| 18305 | YWHAH | 1.548 | 0.160 | 12 | <.0001 | 0.0007 | 2.92 | UP |
| 37812 | TEAD1 | 1.522 | 0.288 | 12 | 0.0002 | 0.0385 | 2.87 | UP |
| 44453 | YWHAH | 1.518 | 0.167 | 12 | <.0001 | 0.0009 | 2.86 | UP |
| 40874 | YWHAH | 1.508 | 0.180 | 12 | <.0001 | 0.0014 | 2.84 | UP |
| 5256 | EMP2 | 1.503 | 0.155 | 12 | <.0001 | 0.0007 | 2.83 | UP |
| 917 | YWHAH | 1.500 | 0.129 | 12 | <.0001 | 0.0002 | 2.83 | UP |
| 6477 | YWHAH | 1.491 | 0.158 | 12 | <.0001 | 0.0008 | 2.81 | UP |
| 1161 | EMP2 | 1.490 | 0.162 | 12 | <.0001 | 0.0009 | 2.81 | UP |
| 24591 | EMP2 | 1.490 | 0.161 | 12 | <.0001 | 0.0009 | 2.81 | UP |
| 50266 | YWHAH | 1.486 | 0.180 | 12 | <.0001 | 0.0015 | 2.80 | UP |
| 36988 | EMP2 | 1.483 | 0.173 | 12 | <.0001 | 0.0012 | 2.80 | UP |
| 18978 | SNRNP27 | 1.482 | 0.169 | 12 | <.0001 | 0.0010 | 2.79 | UP |
| 60802 | EMP2 | 1.476 | 0.177 | 12 | <.0001 | 0.0014 | 2.78 | UP |
| 12227 | EMP2 | 1.458 | 0.162 | 12 | <.0001 | 0.0009 | 2.75 | UP |
| 10787 | EMP2 | 1.453 | 0.169 | 12 | <.0001 | 0.0012 | 2.74 | UP |
| 41421 | FOXO3 | 1.453 | 0.289 | 12 | 0.0003 | 0.0489 | 2.74 | UP |
| 52005 | EMP2 | 1.452 | 0.169 | 12 | <.0001 | 0.0012 | 2.74 | UP |
| 8538 | YWHAH | 1.448 | 0.156 | 12 | <.0001 | 0.0009 | 2.73 | UP |
| 61454 | EMP2 | 1.446 | 0.203 | 12 | <.0001 | 0.0046 | 2.72 | UP |
| 33787 | FOXO3 | 1.445 | 0.254 | 12 | 0.0001 | 0.0252 | 2.72 | UP |
| 37716 | CCDC149 | 1.443 | 0.296 | 12 | 0.0004 | 0.0566 | 2.72 | UP |
| 48140 | DRAM1 | 1.439 | 0.263 | 12 | 0.0001 | 0.0320 | 2.71 | UP |
| 29913 | YWHAH | 1.437 | 0.186 | 12 | <.0001 | 0.0023 | 2.71 | UP |
| 62212 | POLR3E | 1.411 | 0.134 | 12 | <.0001 | 0.0005 | 2.66 | UP |
| 11359 | MFSD6 | 1.402 | 0.310 | 12 | 0.0007 | 0.0838 | 2.64 | UP |
| 42970 | YWHAH | 1.398 | 0.154 | 12 | <.0001 | 0.0009 | 2.63 | UP |
| 38049 | SLC41A1 | 1.393 | 0.185 | 12 | <.0001 | 0.0028 | 2.63 | UP |
| 39054 | LSM14A | 1.387 | 0.153 | 12 | <.0001 | 0.0009 | 2.62 | UP |
| 6497 | LSM14A | 1.362 | 0.191 | 12 | <.0001 | 0.0046 | 2.57 | UP |
| 29609 | A_24_P358205 | 1.356 | 0.267 | 12 | 0.0003 | 0.0475 | 2.56 | UP |
| 41187 | ILF3 | 1.353 | 0.153 | 12 | <.0001 | 0.0010 | 2.55 | UP |
| 35799 | DNMT1 | 1.336 | 0.168 | 12 | <.0001 | 0.0018 | 2.53 | UP |
| 834 | PHLDA1 | 1.330 | 0.288 | 12 | 0.0006 | 0.0749 | 2.51 | UP |
| 34987 | EMP2 | 1.330 | 0.170 | 12 | <.0001 | 0.0021 | 2.51 | UP |
| 13135 | KIAA0100 | 1.329 | 0.286 | 12 | 0.0006 | 0.0739 | 2.51 | UP |
| 45397 | TFAP2C | 1.262 | 0.252 | 12 | 0.0003 | 0.0501 | 2.40 | UP |
| 48000 | TFAP2C | 1.260 | 0.207 | 12 | <.0001 | 0.0154 | 2.39 | UP |
| 60486 | SEMA4F | 1.241 | 0.286 | 12 | 0.0010 | 0.1002 | 2.36 | UP |
| 12299 | SH3PXD2A | 1.239 | 0.223 | 12 | 0.0001 | 0.0293 | 2.36 | UP |
| 9429 | STEAP3 | 1.236 | 0.244 | 12 | 0.0003 | 0.0475 | 2.36 | UP |
| 50329 | CCDC149 | 1.234 | 0.235 | 12 | 0.0002 | 0.0393 | 2.35 | UP |
| 39260 | A_24_P255314 | 1.229 | 0.242 | 12 | 0.0003 | 0.0475 | 2.34 | UP |
| 45710 | ANO10 | 1.217 | 0.077 | 12 | <.0001 | <.0001 | 2.32 | UP |
| 46540 | CLIC5 | 1.215 | 0.281 | 12 | 0.0010 | 0.1008 | 2.32 | UP |
| 11062 | ZBTB47 | 1.209 | 0.223 | 12 | 0.0002 | 0.0333 | 2.31 | UP |
| 35177 | IL6 | 1.187 | 0.244 | 12 | 0.0004 | 0.0566 | 2.28 | UP |
| 10106 | SNX27 | 1.186 | 0.198 | 12 | <.0001 | 0.0180 | 2.27 | UP |
| 33144 | CCDC149 | 1.163 | 0.201 | 12 | <.0001 | 0.0228 | 2.24 | UP |
| 2658 | FNBP1 | 1.158 | 0.185 | 12 | <.0001 | 0.0125 | 2.23 | UP |
| 37433 | NFIB | 1.156 | 0.218 | 12 | 0.0002 | 0.0376 | 2.23 | UP |
| 47325 | NFIB | 1.149 | 0.229 | 12 | 0.0003 | 0.0496 | 2.22 | UP |
| 50721 | KCNS3 | 1.146 | 0.206 | 12 | 0.0001 | 0.0293 | 2.21 | UP |
| 34629 | ANO10 | 1.135 | 0.190 | 12 | <.0001 | 0.0180 | 2.20 | UP |
| 30636 | EIF4E2 | 1.114 | 0.246 | 12 | 0.0007 | 0.0824 | 2.16 | UP |
| 3711 | ARG2 | 1.111 | 0.192 | 12 | <.0001 | 0.0228 | 2.16 | UP |
| 51495 | TFAP2C | 1.106 | 0.210 | 12 | 0.0002 | 0.0390 | 2.15 | UP |
| 3138 | WHAMM | 1.088 | 0.183 | 12 | <.0001 | 0.0180 | 2.13 | UP |
| 54891 | TFAP2C | 1.082 | 0.198 | 12 | 0.0001 | 0.0323 | 2.12 | UP |
| 15685 | A_24_P84608 | 1.060 | 0.230 | 12 | 0.0006 | 0.0764 | 2.08 | UP |
| 5183 | TFAP2C | 1.052 | 0.215 | 12 | 0.0004 | 0.0556 | 2.07 | UP |
| 60108 | METTL16 | 1.045 | 0.161 | 12 | <.0001 | 0.0099 | 2.06 | UP |
| 54103 | ALDH9A1 | 1.044 | 0.228 | 12 | 0.0006 | 0.0784 | 2.06 | UP |
| 38199 | RNF144A | 1.042 | 0.220 | 12 | 0.0005 | 0.0671 | 2.06 | UP |
| 12098 | TFAP2C | 1.020 | 0.211 | 12 | 0.0004 | 0.0589 | 2.03 | UP |
| 25842 | ARID1B | 1.019 | 0.140 | 12 | <.0001 | 0.0039 | 2.03 | UP |
| 56330 | AKAP8 | 1.018 | 0.165 | 12 | <.0001 | 0.0136 | 2.02 | UP |
| 55927 | LBH | 1.014 | 0.219 | 12 | 0.0006 | 0.0749 | 2.02 | UP |
