# Supplementary material for: FOXM1 expression is significantly associated with chemotherapy resistance and adverse prognosis in non-serous epithelial ovarian cancer patients
Source: J Exp Clin Cancer Res. 2017 May 8;36:63. doi: 10.1186/s13046-017-0536-y (PMC5422964; doi:10.1186/s13046-017-0536-y)
Supplement: Supplementary file 8 — Figure S5: Volcano plot displaying differential expressed genes between siFOXM1 and siControl OSPC2 cells. Table S13: List of down-regulated genes in siFOXM1 OSPC2 cells. Table S14: List of up-regulated genes in siFOXM1 OSPC2 cells. (DOCX 260 kb) [file 13046_2017_536_MOESM8_ESM.docx]

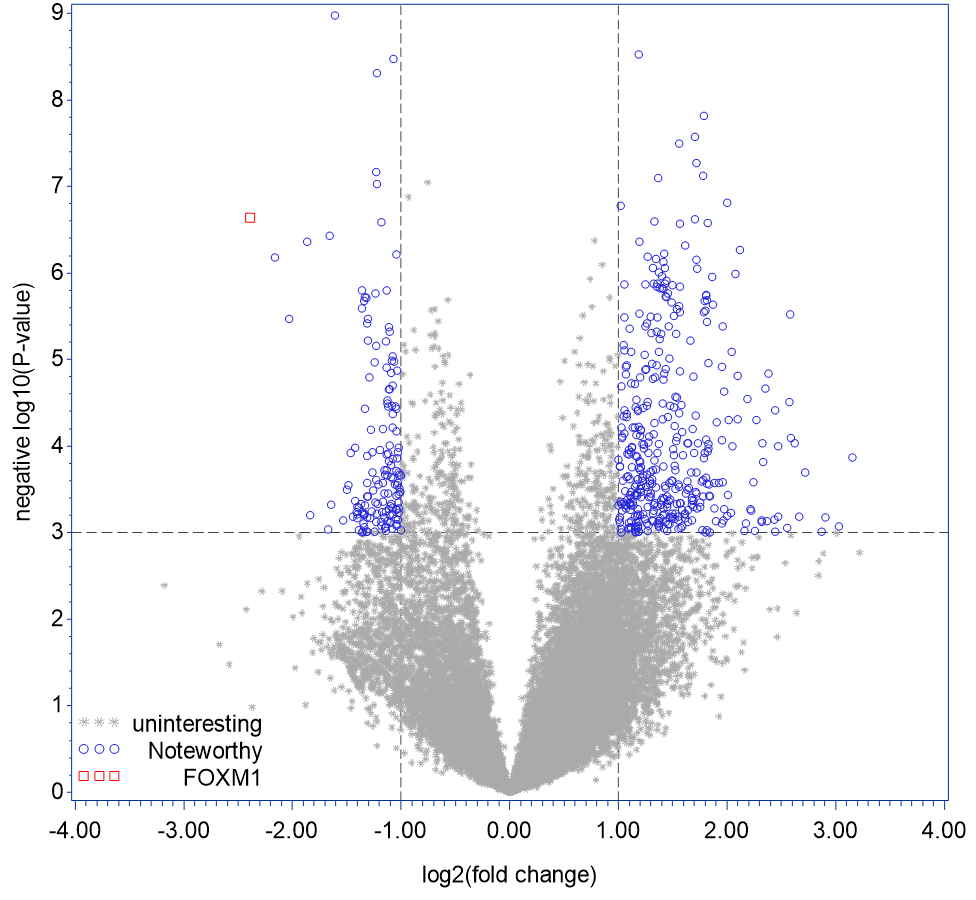


**Figure S5.** Volcano plot displaying differential expressed genes between siFOXM1 and siControl OSPC2 cells. The horizontal axis shows the base-2 logarithms of gene-expression fold changes for siFOXM1 cells compared to siControl cells. Vertical reference lines at +1.00 and –1.00 denote expression changes of 2 folds up and 2 folds down, respectively; data points outside these two vertical reference lines denote genes that show a >2-fold change in expression. The vertical axis shows the negative of the base-10 logarithm of the unadjusted P-values associated with expression changes. The horizontal line at 3 denotes unadjusted P=0.001; data points above this horizontal reference line thus have P<0.001 associated with their expression change. The red square denotes FOXM1. Blue circles denote non-FOXM1 genes that meet the double requirement of >2-fold change and P<0.001, whereas gray asterisks denote genes that do not meet the double requirement.

| **FeatureNum** | **GeneName** | **Estimate** | **Standard Error** | **DF** | **Pr > \|t\|** | **False Discovery Rate p-value** | **Fold Change** | **Direction of Change** |
| --- | --- | --- | --- | --- | --- | --- | --- | --- |
| 55112 | FOXM1 | -2.392 | 0.229 | 12 | <.0001 | 0.0003 | 5.25 | Down |
| 59559 | CA2 | -2.164 | 0.229 | 12 | <.0001 | 0.0005 | 4.48 | Down |
| 52089 | CYR61 | -2.033 | 0.251 | 12 | <.0001 | 0.0008 | 4.09 | Down |
| 23244 | DDAH1 | -1.863 | 0.190 | 12 | <.0001 | 0.0004 | 3.64 | Down |
| 292 | B4GALNT1 | -1.837 | 0.401 | 12 | 0.0006 | 0.0181 | 3.57 | Down |
| 30136 | FABP6 | -1.670 | 0.382 | 12 | 0.0009 | 0.0220 | 3.18 | Down |
| 59536 | DNAJB6 | -1.660 | 0.167 | 12 | <.0001 | 0.0004 | 3.16 | Down |
| 51067 | UBC | -1.645 | 0.347 | 12 | 0.0005 | 0.0159 | 3.13 | Down |
| 3349 | FAM96A | -1.607 | 0.096 | 12 | <.0001 | <.0001 | 3.05 | Down |
| 7358 | THC2550570 | -1.537 | 0.341 | 12 | 0.0007 | 0.0193 | 2.90 | Down |
| 44925 | RPL26 | -1.498 | 0.301 | 12 | 0.0003 | 0.0130 | 2.82 | Down |
| 16300 | UBC | -1.484 | 0.294 | 12 | 0.0003 | 0.0122 | 2.80 | Down |
| 40031 | RPL22L1 | -1.468 | 0.263 | 12 | 0.0001 | 0.0076 | 2.77 | Down |
| 47806 | ANXA2 | -1.445 | 0.317 | 12 | 0.0007 | 0.0185 | 2.72 | Down |
| 46422 | RPL17 | -1.425 | 0.297 | 12 | 0.0004 | 0.0154 | 2.69 | Down |
| 39069 | H3F3A | -1.420 | 0.251 | 12 | 0.0001 | 0.0071 | 2.68 | Down |
| 21429 | RPS23 | -1.407 | 0.304 | 12 | 0.0006 | 0.0173 | 2.65 | Down |
| 47396 | RPL5 | -1.406 | 0.306 | 12 | 0.0006 | 0.0180 | 2.65 | Down |
| 22553 | RPL26 | -1.405 | 0.310 | 12 | 0.0007 | 0.0189 | 2.65 | Down |
| 26048 | CCNB1 | -1.403 | 0.298 | 12 | 0.0005 | 0.0161 | 2.64 | Down |
| 8580 | CCNB1 | -1.389 | 0.295 | 12 | 0.0005 | 0.0163 | 2.62 | Down |
| 46979 | CCNB1 | -1.388 | 0.319 | 12 | 0.0009 | 0.0224 | 2.62 | Down |
| 28683 | CCNB1 | -1.370 | 0.288 | 12 | 0.0005 | 0.0158 | 2.59 | Down |
| 21842 | RPL11 | -1.364 | 0.315 | 12 | 0.0010 | 0.0229 | 2.57 | Down |
| 46839 | DNAJB6 | -1.364 | 0.157 | 12 | <.0001 | 0.0006 | 2.57 | Down |
| 10572 | KRT18P55 | -1.364 | 0.294 | 12 | 0.0006 | 0.0173 | 2.57 | Down |
| 27636 | GNG11 | -1.361 | 0.164 | 12 | <.0001 | 0.0008 | 2.57 | Down |
| 41979 | CCNB1 | -1.354 | 0.313 | 12 | 0.0010 | 0.0228 | 2.56 | Down |
| 54092 | CCNB1 | -1.350 | 0.293 | 12 | 0.0006 | 0.0177 | 2.55 | Down |
| 33193 | XLOC_l2_0019 | -1.345 | 0.302 | 12 | 0.0008 | 0.0202 | 2.54 | Down |
| 59036 | RAN | -1.343 | 0.305 | 12 | 0.0009 | 0.0213 | 2.54 | Down |
| 26235 | GNG11 | -1.340 | 0.158 | 12 | <.0001 | 0.0007 | 2.53 | Down |
| 44929 | CCNB1 | -1.339 | 0.291 | 12 | 0.0006 | 0.0177 | 2.53 | Down |
| 18680 | RPL21 | -1.337 | 0.292 | 12 | 0.0006 | 0.0181 | 2.53 | Down |
| 42636 | RPL5 | -1.337 | 0.282 | 12 | 0.0005 | 0.0159 | 2.53 | Down |
| 8310 | A_24_P169843 | -1.336 | 0.211 | 12 | <.0001 | 0.0038 | 2.52 | Down |
| 23049 | GNG11 | -1.332 | 0.156 | 12 | <.0001 | 0.0007 | 2.52 | Down |
| 58079 | GNG11 | -1.322 | 0.155 | 12 | <.0001 | 0.0007 | 2.50 | Down |
| 62204 | RPL4 | -1.321 | 0.305 | 12 | 0.0010 | 0.0228 | 2.50 | Down |
| 11290 | THC2507627 | -1.316 | 0.270 | 12 | 0.0004 | 0.0143 | 2.49 | Down |
| 49675 | RPL26 | -1.315 | 0.259 | 12 | 0.0003 | 0.0119 | 2.49 | Down |
| 38850 | HOXB6 | -1.315 | 0.289 | 12 | 0.0007 | 0.0187 | 2.49 | Down |
| 15443 | H3F3A | -1.313 | 0.237 | 12 | 0.0001 | 0.0079 | 2.48 | Down |
| 35413 | CCNB1 | -1.310 | 0.302 | 12 | 0.0010 | 0.0228 | 2.48 | Down |
| 32518 | GNG11 | -1.310 | 0.164 | 12 | <.0001 | 0.0009 | 2.48 | Down |
| 52879 | RPL7 | -1.306 | 0.268 | 12 | 0.0004 | 0.0144 | 2.47 | Down |
| 1063 | GNG11 | -1.305 | 0.171 | 12 | <.0001 | 0.0012 | 2.47 | Down |
| 15682 | GNG11 | -1.303 | 0.161 | 12 | <.0001 | 0.0008 | 2.47 | Down |
| 57399 | GNG11 | -1.292 | 0.187 | 12 | <.0001 | 0.0022 | 2.45 | Down |
| 42600 | PRPS1 | -1.282 | 0.282 | 12 | 0.0007 | 0.0188 | 2.43 | Down |
| 13427 | A_19_P008089 | -1.275 | 0.214 | 12 | <.0001 | 0.0054 | 2.42 | Down |
| 42258 | A_24_P186944 | -1.268 | 0.274 | 12 | 0.0006 | 0.0172 | 2.41 | Down |
| 15854 | A_24_P247454 | -1.267 | 0.241 | 12 | 0.0002 | 0.0105 | 2.41 | Down |
| 7030 | POTEM | -1.267 | 0.227 | 12 | 0.0001 | 0.0076 | 2.41 | Down |
| 44243 | TPT1 | -1.259 | 0.253 | 12 | 0.0003 | 0.0131 | 2.39 | Down |
| 50457 | CXCR4 | -1.247 | 0.173 | 12 | <.0001 | 0.0018 | 2.37 | Down |
| 12229 | UBB | -1.245 | 0.287 | 12 | 0.0010 | 0.0228 | 2.37 | Down |
| 35701 | CXCR4 | -1.235 | 0.143 | 12 | <.0001 | 0.0007 | 2.35 | Down |
| 42918 | A_24_P366415 | -1.234 | 0.258 | 12 | 0.0004 | 0.0157 | 2.35 | Down |
| 35112 | RPL24 | -1.232 | 0.275 | 12 | 0.0008 | 0.0198 | 2.35 | Down |
| 35839 | EDN1 | -1.232 | 0.106 | 12 | <.0001 | 0.0002 | 2.35 | Down |
| 29195 | A_24_P15821 | -1.231 | 0.240 | 12 | 0.0002 | 0.0115 | 2.35 | Down |
| 55481 | GNG11 | -1.229 | 0.163 | 12 | <.0001 | 0.0013 | 2.34 | Down |
| 5044 | APOO | -1.223 | 0.083 | 12 | <.0001 | <.0001 | 2.33 | Down |
| 33693 | CXCR4 | -1.221 | 0.108 | 12 | <.0001 | 0.0002 | 2.33 | Down |
| 48715 | A_24_P24790 | -1.220 | 0.265 | 12 | 0.0006 | 0.0177 | 2.33 | Down |
| 42836 | LOC729313 | -1.215 | 0.255 | 12 | 0.0005 | 0.0158 | 2.32 | Down |
| 724 | A_24_P606663 | -1.209 | 0.239 | 12 | 0.0003 | 0.0121 | 2.31 | Down |
| 38451 | RPL17 | -1.198 | 0.213 | 12 | 0.0001 | 0.0073 | 2.29 | Down |
| 209 | RPL23AP7 | -1.183 | 0.264 | 12 | 0.0008 | 0.0198 | 2.27 | Down |
| 57529 | WSB2 | -1.181 | 0.115 | 12 | <.0001 | 0.0003 | 2.27 | Down |
| 36702 | A_33_P327732 | -1.179 | 0.267 | 12 | 0.0008 | 0.0208 | 2.26 | Down |
| 10900 | LOC100288602 | -1.175 | 0.254 | 12 | 0.0006 | 0.0175 | 2.26 | Down |
| 23755 | EBNA1BP2 | -1.171 | 0.196 | 12 | <.0001 | 0.0054 | 2.25 | Down |
| 12714 | PPIA | -1.169 | 0.244 | 12 | 0.0004 | 0.0156 | 2.25 | Down |
| 25834 | ODC1 | -1.165 | 0.229 | 12 | 0.0003 | 0.0119 | 2.24 | Down |
| 36381 | CXCR4 | -1.154 | 0.235 | 12 | 0.0004 | 0.0138 | 2.23 | Down |
| 52251 | PSMA7 | -1.151 | 0.253 | 12 | 0.0007 | 0.0187 | 2.22 | Down |
| 25428 | PPIA | -1.151 | 0.217 | 12 | 0.0002 | 0.0102 | 2.22 | Down |
| 21094 | CR595167 | -1.149 | 0.220 | 12 | 0.0002 | 0.0108 | 2.22 | Down |
| 23031 | XLOC_l2_0021 | -1.149 | 0.262 | 12 | 0.0009 | 0.0215 | 2.22 | Down |
| 36364 | CCT4 | -1.142 | 0.263 | 12 | 0.0010 | 0.0227 | 2.21 | Down |
| 47379 | ZMYND17 | -1.138 | 0.258 | 12 | 0.0009 | 0.0213 | 2.20 | Down |
| 43856 | EDN1 | -1.138 | 0.149 | 12 | <.0001 | 0.0012 | 2.20 | Down |
| 34855 | POTEKP | -1.137 | 0.245 | 12 | 0.0006 | 0.0172 | 2.20 | Down |
| 42276 | EBNA1BP2 | -1.133 | 0.204 | 12 | 0.0001 | 0.0078 | 2.19 | Down |
| 49757 | EDN1 | -1.132 | 0.159 | 12 | <.0001 | 0.0018 | 2.19 | Down |
| 4996 | EBNA1BP2 | -1.132 | 0.214 | 12 | 0.0002 | 0.0102 | 2.19 | Down |
| 20618 | CXCR4 | -1.131 | 0.130 | 12 | <.0001 | 0.0006 | 2.19 | Down |
| 62708 | PGK1 | -1.129 | 0.252 | 12 | 0.0007 | 0.0197 | 2.19 | Down |
| 15767 | A_24_P878388 | -1.129 | 0.217 | 12 | 0.0002 | 0.0109 | 2.19 | Down |
| 11678 | F3 | -1.128 | 0.174 | 12 | <.0001 | 0.0034 | 2.19 | Down |
| 28523 | EBNA1BP2 | -1.127 | 0.175 | 12 | <.0001 | 0.0035 | 2.18 | Down |
| 46273 | COX2 | -1.120 | 0.228 | 12 | 0.0004 | 0.0138 | 2.17 | Down |
| 47010 | CXCR4 | -1.120 | 0.175 | 12 | <.0001 | 0.0037 | 2.17 | Down |
| 21708 | TRIB1 | -1.117 | 0.200 | 12 | 0.0001 | 0.0076 | 2.17 | Down |
| 32048 | CXCR4 | -1.116 | 0.167 | 12 | <.0001 | 0.0027 | 2.17 | Down |
| 49063 | EDN1 | -1.115 | 0.141 | 12 | <.0001 | 0.0010 | 2.17 | Down |
| 30843 | TMSB4X | -1.114 | 0.238 | 12 | 0.0005 | 0.0165 | 2.16 | Down |
| 456 | EBNA1BP2 | -1.109 | 0.204 | 12 | 0.0002 | 0.0087 | 2.16 | Down |
| 14695 | KPNA2 | -1.108 | 0.242 | 12 | 0.0006 | 0.0181 | 2.16 | Down |
| 9216 | EDN1 | -1.107 | 0.165 | 12 | <.0001 | 0.0027 | 2.15 | Down |
| 6704 | EBNA1BP2 | -1.105 | 0.194 | 12 | <.0001 | 0.0069 | 2.15 | Down |
| 31380 | CXCR4 | -1.103 | 0.141 | 12 | <.0001 | 0.0011 | 2.15 | Down |
| 32574 | FGFBP1 | -1.101 | 0.246 | 12 | 0.0008 | 0.0198 | 2.15 | Down |
| 51678 | PGK1 | -1.097 | 0.232 | 12 | 0.0005 | 0.0161 | 2.14 | Down |
| 18356 | H2AFZ | -1.095 | 0.230 | 12 | 0.0005 | 0.0159 | 2.14 | Down |
| 48391 | EBNA1BP2 | -1.094 | 0.203 | 12 | 0.0002 | 0.0093 | 2.13 | Down |
| 15300 | EDN1 | -1.091 | 0.156 | 12 | <.0001 | 0.0020 | 2.13 | Down |
| 8600 | EBNA1BP2 | -1.089 | 0.210 | 12 | 0.0002 | 0.0111 | 2.13 | Down |
| 59502 | ZBED2 | -1.087 | 0.150 | 12 | <.0001 | 0.0017 | 2.12 | Down |
| 55846 | AGPAT9 | -1.087 | 0.170 | 12 | <.0001 | 0.0036 | 2.12 | Down |
| 5664 | PGK1 | -1.083 | 0.247 | 12 | 0.0009 | 0.0218 | 2.12 | Down |
| 35241 | CCT3 | -1.080 | 0.230 | 12 | 0.0005 | 0.0163 | 2.11 | Down |
| 27476 | RPS7P5 | -1.079 | 0.209 | 12 | 0.0002 | 0.0113 | 2.11 | Down |
| 39873 | PGK1 | -1.079 | 0.235 | 12 | 0.0006 | 0.0179 | 2.11 | Down |
| 11310 | CXCR4 | -1.076 | 0.159 | 12 | <.0001 | 0.0025 | 2.11 | Down |
| 23709 | EDN1 | -1.076 | 0.179 | 12 | <.0001 | 0.0052 | 2.11 | Down |
| 24052 | CXCR4 | -1.075 | 0.147 | 12 | <.0001 | 0.0016 | 2.11 | Down |
| 29748 | EBNA1BP2 | -1.075 | 0.172 | 12 | <.0001 | 0.0041 | 2.11 | Down |
| 24447 | EBNA1BP2 | -1.075 | 0.196 | 12 | 0.0001 | 0.0083 | 2.11 | Down |
| 44309 | RPIA | -1.072 | 0.071 | 12 | <.0001 | <.0001 | 2.10 | Down |
| 57001 | EDN1 | -1.064 | 0.147 | 12 | <.0001 | 0.0017 | 2.09 | Down |
| 29783 | A_23_P109677 | -1.059 | 0.209 | 12 | 0.0003 | 0.0121 | 2.08 | Down |
| 61418 | A_33_P324699 | -1.051 | 0.165 | 12 | <.0001 | 0.0037 | 2.07 | Down |
| 57711 | PGK1 | -1.050 | 0.232 | 12 | 0.0007 | 0.0191 | 2.07 | Down |
| 35886 | PGK1 | -1.046 | 0.235 | 12 | 0.0008 | 0.0205 | 2.06 | Down |
| 62038 | A_23_P109677 | -1.045 | 0.176 | 12 | <.0001 | 0.0055 | 2.06 | Down |
| 55897 | RPL21 | -1.044 | 0.223 | 12 | 0.0005 | 0.0165 | 2.06 | Down |
| 14518 | POTEE | -1.043 | 0.224 | 12 | 0.0005 | 0.0167 | 2.06 | Down |
| 37095 | RBM8A | -1.043 | 0.213 | 12 | 0.0004 | 0.0142 | 2.06 | Down |
| 15381 | C1orf43 | -1.043 | 0.110 | 12 | <.0001 | 0.0005 | 2.06 | Down |
| 18461 | RPL21 | -1.042 | 0.197 | 12 | 0.0002 | 0.0102 | 2.06 | Down |
| 50820 | EDN1 | -1.042 | 0.164 | 12 | <.0001 | 0.0038 | 2.06 | Down |
| 4537 | TUBA1A | -1.041 | 0.235 | 12 | 0.0008 | 0.0208 | 2.06 | Down |
| 47626 | EDN1 | -1.038 | 0.147 | 12 | <.0001 | 0.0019 | 2.05 | Down |
| 58047 | A_24_P358131 | -1.036 | 0.205 | 12 | 0.0003 | 0.0121 | 2.05 | Down |
| 62314 | A_23_P109677 | -1.035 | 0.189 | 12 | 0.0001 | 0.0082 | 2.05 | Down |
| 50746 | RBM8A | -1.033 | 0.229 | 12 | 0.0007 | 0.0192 | 2.05 | Down |
| 56087 | A_33_P339087 | -1.030 | 0.184 | 12 | 0.0001 | 0.0076 | 2.04 | Down |
| 10520 | A_23_P109677 | -1.028 | 0.202 | 12 | 0.0003 | 0.0119 | 2.04 | Down |
| 2183 | A_23_P109677 | -1.025 | 0.209 | 12 | 0.0004 | 0.0140 | 2.03 | Down |
| 21021 | A_23_P109677 | -1.023 | 0.214 | 12 | 0.0004 | 0.0157 | 2.03 | Down |
| 45470 | RBM8A | -1.022 | 0.221 | 12 | 0.0006 | 0.0175 | 2.03 | Down |
| 27361 | A_33_P323941 | -1.022 | 0.181 | 12 | 0.0001 | 0.0071 | 2.03 | Down |
| 53041 | A_23_P109677 | -1.022 | 0.194 | 12 | 0.0002 | 0.0105 | 2.03 | Down |
| 57157 | RBM8A | -1.020 | 0.218 | 12 | 0.0005 | 0.0165 | 2.03 | Down |
| 17565 | A_23_P109677 | -1.015 | 0.195 | 12 | 0.0002 | 0.0108 | 2.02 | Down |
| 19269 | THC2598763 | -1.015 | 0.203 | 12 | 0.0003 | 0.0127 | 2.02 | Down |
| 23243 | ENST00000407 | -1.011 | 0.204 | 12 | 0.0003 | 0.0134 | 2.01 | Down |
| 12996 | PSMC1 | -1.005 | 0.231 | 12 | 0.0009 | 0.0224 | 2.01 | Down |
| 28726 | A_23_P109677 | -1.001 | 0.192 | 12 | 0.0002 | 0.0108 | 2.00 | Down |

| **FeatureNum** | **GeneName** | **Estimate** | **Standard Error** | **DF** | **Pr > \|t\|** | **False Discovery Rate p-value** | **Fold Change** | **Direction of Change** |
| --- | --- | --- | --- | --- | --- | --- | --- | --- |
| 32309 | MTL5 | 3.153 | 0.573 | 12 | 0.0001 | 0.0081 | 8.90 | UP |
| 41665 | TK2 | 3.031 | 0.688 | 12 | 0.0009 | 0.0213 | 8.17 | UP |
| 40810 | PRRX2 | 2.906 | 0.639 | 12 | 0.0007 | 0.0187 | 7.49 | UP |
| 11150 | MPP2 | 2.869 | 0.663 | 12 | 0.0010 | 0.0228 | 7.30 | UP |
| 20922 | F2R | 2.722 | 0.517 | 12 | 0.0002 | 0.0105 | 6.60 | UP |
| 22193 | DUSP13 | 2.665 | 0.585 | 12 | 0.0007 | 0.0185 | 6.34 | UP |
| 44266 | SH3D21 | 2.619 | 0.456 | 12 | <.0001 | 0.0067 | 6.14 | UP |
| 40012 | PDPR | 2.587 | 0.444 | 12 | <.0001 | 0.0062 | 6.01 | UP |
| 38208 | NEURL3 | 2.581 | 0.316 | 12 | <.0001 | 0.0008 | 5.98 | UP |
| 28741 | LOC100505585 | 2.570 | 0.398 | 12 | <.0001 | 0.0035 | 5.94 | UP |
| 62465 | LOH12CR2 | 2.553 | 0.582 | 12 | 0.0009 | 0.0216 | 5.87 | UP |
| 4152 | RASSF2 | 2.472 | 0.435 | 12 | 0.0001 | 0.0070 | 5.55 | UP |
| 44647 | ENST00000451 | 2.469 | 0.542 | 12 | 0.0007 | 0.0185 | 5.54 | UP |
| 5142 | FAM59B | 2.444 | 0.565 | 12 | 0.0010 | 0.0228 | 5.44 | UP |
| 8877 | LOC645722 | 2.443 | 0.387 | 12 | <.0001 | 0.0039 | 5.44 | UP |
| 45833 | CCDC146 | 2.433 | 0.539 | 12 | 0.0007 | 0.0192 | 5.40 | UP |
| 60702 | IGFBP7 | 2.380 | 0.341 | 12 | <.0001 | 0.0020 | 5.20 | UP |
| 36069 | C1R | 2.368 | 0.527 | 12 | 0.0007 | 0.0196 | 5.16 | UP |
| 19798 | TMEM63C | 2.351 | 0.351 | 12 | <.0001 | 0.0027 | 5.10 | UP |
| 54979 | TNFSF10 | 2.331 | 0.430 | 12 | 0.0002 | 0.0089 | 5.03 | UP |
| 51127 | SLC16A6 | 2.323 | 0.405 | 12 | <.0001 | 0.0067 | 5.00 | UP |
| 32269 | HSPA12A | 2.317 | 0.515 | 12 | 0.0007 | 0.0195 | 4.98 | UP |
| 15650 | LOC100129268 | 2.316 | 0.517 | 12 | 0.0008 | 0.0198 | 4.98 | UP |
| 51902 | RORC | 2.269 | 0.369 | 12 | <.0001 | 0.0045 | 4.82 | UP |
| 50730 | ALDH7A1 | 2.255 | 0.520 | 12 | 0.0010 | 0.0227 | 4.77 | UP |
| 41327 | SLC16A2 | 2.244 | 0.440 | 12 | 0.0003 | 0.0118 | 4.74 | UP |
| 46919 | FOXA2 | 2.221 | 0.478 | 12 | 0.0006 | 0.0170 | 4.66 | UP |
| 16953 | LOC388780 | 2.213 | 0.473 | 12 | 0.0005 | 0.0165 | 4.64 | UP |
| 44337 | IGF2 | 2.187 | 0.336 | 12 | <.0001 | 0.0033 | 4.55 | UP |
| 34220 | PANK3 | 2.170 | 0.487 | 12 | 0.0008 | 0.0201 | 4.50 | UP |
| 40677 | TMEM229B | 2.159 | 0.497 | 12 | 0.0010 | 0.0226 | 4.47 | UP |
| 38199 | RNF144A | 2.119 | 0.220 | 12 | <.0001 | 0.0004 | 4.34 | UP |
| 30248 | SPON2 | 2.096 | 0.302 | 12 | <.0001 | 0.0021 | 4.27 | UP |
| 28198 | MRAS | 2.095 | 0.341 | 12 | <.0001 | 0.0045 | 4.27 | UP |
| 51865 | LYPD6 | 2.078 | 0.229 | 12 | <.0001 | 0.0006 | 4.22 | UP |
| 28108 | TMEM50B | 2.049 | 0.360 | 12 | 0.0001 | 0.0070 | 4.14 | UP |
| 13894 | SLC2A5 | 2.041 | 0.276 | 12 | <.0001 | 0.0015 | 4.12 | UP |
| 3555 | ACPL2 | 2.036 | 0.441 | 12 | 0.0006 | 0.0175 | 4.10 | UP |
| 26136 | LOC100287616 | 2.013 | 0.328 | 12 | <.0001 | 0.0045 | 4.04 | UP |
| 62066 | GSTO2 | 2.010 | 0.410 | 12 | 0.0004 | 0.0141 | 4.03 | UP |
| 29524 | TCF7L1 | 2.003 | 0.438 | 12 | 0.0006 | 0.0183 | 4.01 | UP |
| 38049 | SLC41A1 | 1.998 | 0.185 | 12 | <.0001 | 0.0002 | 4.00 | UP |
| 37716 | CCDC149 | 1.972 | 0.296 | 12 | <.0001 | 0.0028 | 3.92 | UP |
| 62917 | H2BFM | 1.971 | 0.421 | 12 | 0.0005 | 0.0165 | 3.92 | UP |
| 28644 | ANKRD43 | 1.967 | 0.437 | 12 | 0.0007 | 0.0195 | 3.91 | UP |
| 6772 | ASAP3 | 1.960 | 0.247 | 12 | <.0001 | 0.0010 | 3.89 | UP |
| 38318 | SAR1B | 1.957 | 0.384 | 12 | 0.0003 | 0.0119 | 3.88 | UP |
| 44694 | LINC00174 | 1.954 | 0.438 | 12 | 0.0008 | 0.0201 | 3.87 | UP |
| 8080 | ASPRV1 | 1.952 | 0.274 | 12 | <.0001 | 0.0018 | 3.87 | UP |
| 2797 | LOC100505932 | 1.949 | 0.337 | 12 | <.0001 | 0.0065 | 3.86 | UP |
| 59027 | ENST00000445 | 1.922 | 0.378 | 12 | 0.0003 | 0.0119 | 3.79 | UP |
| 6882 | BCOR | 1.905 | 0.312 | 12 | <.0001 | 0.0047 | 3.75 | UP |
| 52188 | ABHD1 | 1.897 | 0.423 | 12 | 0.0007 | 0.0197 | 3.72 | UP |
| 55181 | AQP1 | 1.886 | 0.371 | 12 | 0.0003 | 0.0119 | 3.70 | UP |
| 7086 | HIP1 | 1.886 | 0.402 | 12 | 0.0005 | 0.0164 | 3.70 | UP |
| 12299 | SH3PXD2A | 1.867 | 0.223 | 12 | <.0001 | 0.0007 | 3.65 | UP |
| 30115 | THG1L | 1.860 | 0.207 | 12 | <.0001 | 0.0006 | 3.63 | UP |
| 34388 | RUNX2 | 1.839 | 0.426 | 12 | 0.0010 | 0.0229 | 3.58 | UP |
| 62821 | LOC439911 | 1.839 | 0.377 | 12 | 0.0004 | 0.0143 | 3.58 | UP |
| 37788 | PRR15L | 1.836 | 0.376 | 12 | 0.0004 | 0.0142 | 3.57 | UP |
| 18 | CYB5R4 | 1.835 | 0.363 | 12 | 0.0003 | 0.0121 | 3.57 | UP |
| 3700 | LOC728392 | 1.828 | 0.323 | 12 | 0.0001 | 0.0072 | 3.55 | UP |
| 57242 | LRRK1 | 1.828 | 0.319 | 12 | <.0001 | 0.0067 | 3.55 | UP |
| 18854 | THG1L | 1.827 | 0.254 | 12 | <.0001 | 0.0018 | 3.55 | UP |
| 11130 | SLC22A23 | 1.821 | 0.177 | 12 | <.0001 | 0.0003 | 3.53 | UP |
| 42018 | LOC389634 | 1.820 | 0.325 | 12 | 0.0001 | 0.0074 | 3.53 | UP |
| 61607 | SEMA4D | 1.818 | 0.417 | 12 | 0.0009 | 0.0224 | 3.53 | UP |
| 52756 | THG1L | 1.816 | 0.214 | 12 | <.0001 | 0.0007 | 3.52 | UP |
| 58089 | PRR15L | 1.816 | 0.358 | 12 | 0.0003 | 0.0120 | 3.52 | UP |
| 55288 | THG1L | 1.813 | 0.226 | 12 | <.0001 | 0.0009 | 3.51 | UP |
| 34233 | THG1L | 1.810 | 0.211 | 12 | <.0001 | 0.0007 | 3.51 | UP |
| 694 | PRR15L | 1.809 | 0.350 | 12 | 0.0002 | 0.0111 | 3.50 | UP |
| 30626 | THG1L | 1.808 | 0.211 | 12 | <.0001 | 0.0007 | 3.50 | UP |
| 52513 | ZC3HAV1 | 1.807 | 0.311 | 12 | <.0001 | 0.0064 | 3.50 | UP |
| 14268 | THG1L | 1.799 | 0.218 | 12 | <.0001 | 0.0008 | 3.48 | UP |
| 46641 | TCF7 | 1.798 | 0.416 | 12 | 0.0010 | 0.0229 | 3.48 | UP |
| 6161 | FZD7 | 1.796 | 0.212 | 12 | <.0001 | 0.0007 | 3.47 | UP |
| 34133 | WNT5A | 1.793 | 0.321 | 12 | 0.0001 | 0.0076 | 3.46 | UP |
| 43193 | TACC2 | 1.788 | 0.135 | 12 | <.0001 | <.0001 | 3.45 | UP |
| 23916 | THG1L | 1.788 | 0.218 | 12 | <.0001 | 0.0008 | 3.45 | UP |
| 53081 | HEG1 | 1.788 | 0.326 | 12 | 0.0001 | 0.0082 | 3.45 | UP |
| 35426 | PRR15L | 1.782 | 0.347 | 12 | 0.0002 | 0.0115 | 3.44 | UP |
| 11845 | EMP2 | 1.781 | 0.154 | 12 | <.0001 | 0.0002 | 3.44 | UP |
| 50811 | PRR15L | 1.778 | 0.365 | 12 | 0.0004 | 0.0143 | 3.43 | UP |
| 10072 | SLC22A15 | 1.770 | 0.407 | 12 | 0.0009 | 0.0225 | 3.41 | UP |
| 49894 | PRR15L | 1.765 | 0.345 | 12 | 0.0003 | 0.0117 | 3.40 | UP |
| 38564 | PRR15L | 1.755 | 0.372 | 12 | 0.0005 | 0.0161 | 3.38 | UP |
| 5746 | FAM160B1 | 1.753 | 0.366 | 12 | 0.0004 | 0.0157 | 3.37 | UP |
| 61970 | HOXA6 | 1.733 | 0.302 | 12 | <.0001 | 0.0067 | 3.32 | UP |
| 19944 | THG1L | 1.727 | 0.188 | 12 | <.0001 | 0.0005 | 3.31 | UP |
| 27959 | LOC440993 | 1.724 | 0.367 | 12 | 0.0005 | 0.0164 | 3.30 | UP |
| 62881 | BEND7 | 1.719 | 0.145 | 12 | <.0001 | 0.0002 | 3.29 | UP |
| 49039 | THG1L | 1.718 | 0.183 | 12 | <.0001 | 0.0005 | 3.29 | UP |
| 42072 | NINL | 1.715 | 0.349 | 12 | 0.0004 | 0.0138 | 3.28 | UP |
| 61937 | DTX4 | 1.715 | 0.326 | 12 | 0.0002 | 0.0105 | 3.28 | UP |
| 10526 | LOC100507002 | 1.713 | 0.275 | 12 | <.0001 | 0.0042 | 3.28 | UP |
| 21106 | TGFB2 | 1.708 | 0.345 | 12 | 0.0003 | 0.0135 | 3.27 | UP |
| 5470 | TIMP3 | 1.707 | 0.135 | 12 | <.0001 | 0.0001 | 3.27 | UP |
| 16347 | STEAP3 | 1.703 | 0.164 | 12 | <.0001 | 0.0003 | 3.26 | UP |
| 33192 | GPER | 1.700 | 0.305 | 12 | 0.0001 | 0.0076 | 3.25 | UP |
| 9429 | STEAP3 | 1.690 | 0.244 | 12 | <.0001 | 0.0021 | 3.23 | UP |
| 56794 | ALDH5A1 | 1.689 | 0.352 | 12 | 0.0004 | 0.0154 | 3.22 | UP |
| 41604 | PRR15L | 1.687 | 0.358 | 12 | 0.0005 | 0.0162 | 3.22 | UP |
| 35972 | PHF8 | 1.686 | 0.282 | 12 | <.0001 | 0.0054 | 3.22 | UP |
| 3110 | THBS4 | 1.679 | 0.354 | 12 | 0.0005 | 0.0159 | 3.20 | UP |
| 39763 | PRR15L | 1.677 | 0.348 | 12 | 0.0004 | 0.0151 | 3.20 | UP |
| 1108 | PRR15L | 1.675 | 0.372 | 12 | 0.0007 | 0.0195 | 3.19 | UP |
| 41792 | SETBP1 | 1.675 | 0.345 | 12 | 0.0004 | 0.0146 | 3.19 | UP |
| 61453 | RHOQ | 1.670 | 0.318 | 12 | 0.0002 | 0.0105 | 3.18 | UP |
| 11747 | LRP4 | 1.661 | 0.218 | 12 | <.0001 | 0.0012 | 3.16 | UP |
| 2681 | SNCA | 1.646 | 0.329 | 12 | 0.0003 | 0.0127 | 3.13 | UP |
| 53887 | ZRSR2 | 1.645 | 0.353 | 12 | 0.0006 | 0.0168 | 3.13 | UP |
| 29959 | MTL5 | 1.642 | 0.286 | 12 | <.0001 | 0.0067 | 3.12 | UP |
| 48341 | TCF7 | 1.640 | 0.366 | 12 | 0.0008 | 0.0198 | 3.12 | UP |
| 4720 | DHTKD1 | 1.637 | 0.285 | 12 | <.0001 | 0.0067 | 3.11 | UP |
| 34841 | TNRC6A | 1.636 | 0.320 | 12 | 0.0003 | 0.0117 | 3.11 | UP |
| 30891 | GXYLT2 | 1.632 | 0.328 | 12 | 0.0003 | 0.0130 | 3.10 | UP |
| 25119 | LHX2 | 1.624 | 0.330 | 12 | 0.0004 | 0.0138 | 3.08 | UP |
| 26290 | ENST00000527 | 1.616 | 0.356 | 12 | 0.0007 | 0.0188 | 3.07 | UP |
| 24220 | TCF7 | 1.614 | 0.350 | 12 | 0.0006 | 0.0177 | 3.06 | UP |
| 24617 | RIMBP3 | 1.613 | 0.166 | 12 | <.0001 | 0.0004 | 3.06 | UP |
| 18723 | LBH | 1.607 | 0.350 | 12 | 0.0006 | 0.0180 | 3.05 | UP |
| 10587 | TCF7 | 1.593 | 0.287 | 12 | 0.0001 | 0.0078 | 3.02 | UP |
| 53266 | CLMN | 1.585 | 0.359 | 12 | 0.0008 | 0.0212 | 3.00 | UP |
| 39223 | LBH | 1.585 | 0.340 | 12 | 0.0005 | 0.0167 | 3.00 | UP |
| 44297 | FGD3 | 1.584 | 0.267 | 12 | <.0001 | 0.0057 | 3.00 | UP |
| 7787 | XLOC_002133 | 1.583 | 0.247 | 12 | <.0001 | 0.0036 | 2.99 | UP |
| 53988 | MAP2K6 | 1.575 | 0.330 | 12 | 0.0005 | 0.0158 | 2.98 | UP |
| 41063 | LBH | 1.572 | 0.358 | 12 | 0.0009 | 0.0215 | 2.97 | UP |
| 11062 | ZBTB47 | 1.568 | 0.223 | 12 | <.0001 | 0.0020 | 2.96 | UP |
| 39054 | LSM14A | 1.567 | 0.153 | 12 | <.0001 | 0.0003 | 2.96 | UP |
| 37263 | LBH | 1.567 | 0.328 | 12 | 0.0005 | 0.0158 | 2.96 | UP |
| 45728 | ADCY9 | 1.566 | 0.179 | 12 | <.0001 | 0.0006 | 2.96 | UP |
| 34629 | ANO10 | 1.563 | 0.190 | 12 | <.0001 | 0.0008 | 2.95 | UP |
| 3889 | ENST00000423 | 1.560 | 0.187 | 12 | <.0001 | 0.0007 | 2.95 | UP |
| 28677 | A_33_P321056 | 1.559 | 0.125 | 12 | <.0001 | 0.0001 | 2.95 | UP |
| 7088 | RAPGEF3 | 1.558 | 0.249 | 12 | <.0001 | 0.0041 | 2.94 | UP |
| 20360 | LBH | 1.557 | 0.310 | 12 | 0.0003 | 0.0125 | 2.94 | UP |
| 23949 | TCF7 | 1.554 | 0.355 | 12 | 0.0009 | 0.0218 | 2.94 | UP |
| 41095 | TCF7 | 1.551 | 0.340 | 12 | 0.0007 | 0.0185 | 2.93 | UP |
| 5666 | LBH | 1.550 | 0.306 | 12 | 0.0003 | 0.0121 | 2.93 | UP |
| 6641 | ASH1L | 1.549 | 0.344 | 12 | 0.0007 | 0.0195 | 2.93 | UP |
| 3183 | AF390550 | 1.541 | 0.324 | 12 | 0.0005 | 0.0158 | 2.91 | UP |
| 43741 | CHST15 | 1.539 | 0.186 | 12 | <.0001 | 0.0008 | 2.91 | UP |
| 33081 | NLGN4X | 1.537 | 0.263 | 12 | <.0001 | 0.0061 | 2.90 | UP |
| 50329 | CCDC149 | 1.536 | 0.235 | 12 | <.0001 | 0.0032 | 2.90 | UP |
| 19560 | TET1 | 1.532 | 0.197 | 12 | <.0001 | 0.0011 | 2.89 | UP |
| 8179 | RAB15 | 1.528 | 0.264 | 12 | <.0001 | 0.0065 | 2.88 | UP |
| 55457 | LBH | 1.526 | 0.331 | 12 | 0.0006 | 0.0177 | 2.88 | UP |
| 22785 | SNCA | 1.526 | 0.288 | 12 | 0.0002 | 0.0102 | 2.88 | UP |
| 53185 | LOC100653296 | 1.525 | 0.305 | 12 | 0.0003 | 0.0128 | 2.88 | UP |
| 19169 | HOXA4 | 1.523 | 0.232 | 12 | <.0001 | 0.0032 | 2.87 | UP |
| 45766 | LHX6 | 1.517 | 0.251 | 12 | <.0001 | 0.0051 | 2.86 | UP |
| 35375 | FGFR3 | 1.516 | 0.282 | 12 | 0.0002 | 0.0094 | 2.86 | UP |
| 4932 | GDF11 | 1.514 | 0.335 | 12 | 0.0007 | 0.0191 | 2.86 | UP |
| 9566 | THC2697887 | 1.513 | 0.273 | 12 | 0.0001 | 0.0078 | 2.85 | UP |
| 38130 | IL11RA | 1.512 | 0.237 | 12 | <.0001 | 0.0037 | 2.85 | UP |
| 1320 | TBL1Y | 1.512 | 0.186 | 12 | <.0001 | 0.0008 | 2.85 | UP |
| 21663 | XLOC_l2_0146 | 1.503 | 0.267 | 12 | 0.0001 | 0.0073 | 2.84 | UP |
| 26676 | RAB26 | 1.502 | 0.331 | 12 | 0.0007 | 0.0187 | 2.83 | UP |
| 34987 | EMP2 | 1.497 | 0.170 | 12 | <.0001 | 0.0006 | 2.82 | UP |
| 16931 | NICN1 | 1.496 | 0.325 | 12 | 0.0006 | 0.0177 | 2.82 | UP |
| 51794 | TNS1 | 1.495 | 0.292 | 12 | 0.0003 | 0.0116 | 2.82 | UP |
| 43954 | METTL16 | 1.493 | 0.328 | 12 | 0.0007 | 0.0185 | 2.82 | UP |
| 60802 | EMP2 | 1.488 | 0.177 | 12 | <.0001 | 0.0007 | 2.80 | UP |
| 2478 | PSPC1 | 1.488 | 0.291 | 12 | 0.0003 | 0.0117 | 2.80 | UP |
| 59540 | RAB3A | 1.479 | 0.231 | 12 | <.0001 | 0.0036 | 2.79 | UP |
| 17647 | LOC100130027 | 1.478 | 0.306 | 12 | 0.0004 | 0.0150 | 2.79 | UP |
| 7946 | CNPY4 | 1.472 | 0.290 | 12 | 0.0003 | 0.0119 | 2.77 | UP |
| 31944 | ZCCHC24 | 1.468 | 0.324 | 12 | 0.0007 | 0.0188 | 2.77 | UP |
| 25171 | FAM49A | 1.467 | 0.330 | 12 | 0.0008 | 0.0203 | 2.76 | UP |
| 33144 | CCDC149 | 1.467 | 0.201 | 12 | <.0001 | 0.0016 | 2.76 | UP |
| 9236 | ATP6V1E2 | 1.464 | 0.185 | 12 | <.0001 | 0.0010 | 2.76 | UP |
| 49666 | AGR2 | 1.462 | 0.305 | 12 | 0.0004 | 0.0155 | 2.75 | UP |
| 52005 | EMP2 | 1.456 | 0.169 | 12 | <.0001 | 0.0007 | 2.74 | UP |
| 60218 | SLC25A40 | 1.453 | 0.326 | 12 | 0.0008 | 0.0203 | 2.74 | UP |
| 37714 | CC2D2A | 1.452 | 0.235 | 12 | <.0001 | 0.0043 | 2.74 | UP |
| 54190 | MSX1 | 1.452 | 0.334 | 12 | 0.0010 | 0.0227 | 2.74 | UP |
| 42865 | BCL2L15 | 1.451 | 0.308 | 12 | 0.0005 | 0.0161 | 2.73 | UP |
| 23763 | LBH | 1.451 | 0.305 | 12 | 0.0005 | 0.0158 | 2.73 | UP |
| 20643 | ZHX3 | 1.448 | 0.297 | 12 | 0.0004 | 0.0143 | 2.73 | UP |
| 10787 | EMP2 | 1.443 | 0.169 | 12 | <.0001 | 0.0007 | 2.72 | UP |
| 1161 | EMP2 | 1.442 | 0.162 | 12 | <.0001 | 0.0006 | 2.72 | UP |
| 58679 | ANXA8L2 | 1.437 | 0.203 | 12 | <.0001 | 0.0019 | 2.71 | UP |
| 6087 | XLOC_007769 | 1.437 | 0.321 | 12 | 0.0008 | 0.0198 | 2.71 | UP |
| 44453 | YWHAH | 1.432 | 0.167 | 12 | <.0001 | 0.0007 | 2.70 | UP |
| 53172 | TCF7 | 1.432 | 0.286 | 12 | 0.0003 | 0.0127 | 2.70 | UP |
| 5256 | EMP2 | 1.431 | 0.155 | 12 | <.0001 | 0.0005 | 2.70 | UP |
| 14187 | TBL1Y | 1.428 | 0.161 | 12 | <.0001 | 0.0006 | 2.69 | UP |
| 47547 | ZNF815 | 1.427 | 0.283 | 12 | 0.0003 | 0.0122 | 2.69 | UP |
| 11914 | TRPV1 | 1.423 | 0.294 | 12 | 0.0004 | 0.0148 | 2.68 | UP |
| 7913 | RPL32P3 | 1.423 | 0.149 | 12 | <.0001 | 0.0005 | 2.68 | UP |
| 22102 | LOC150381 | 1.422 | 0.310 | 12 | 0.0006 | 0.0179 | 2.68 | UP |
| 61454 | EMP2 | 1.421 | 0.203 | 12 | <.0001 | 0.0020 | 2.68 | UP |
| 36176 | C18orf8 | 1.420 | 0.324 | 12 | 0.0009 | 0.0217 | 2.68 | UP |
| 29975 | RAPGEF3 | 1.417 | 0.302 | 12 | 0.0005 | 0.0165 | 2.67 | UP |
| 48937 | LOC145474 | 1.416 | 0.264 | 12 | 0.0002 | 0.0095 | 2.67 | UP |
| 12227 | EMP2 | 1.415 | 0.162 | 12 | <.0001 | 0.0006 | 2.67 | UP |
| 9894 | LAMB2P1 | 1.413 | 0.151 | 12 | <.0001 | 0.0005 | 2.66 | UP |
| 17627 | RNF213 | 1.413 | 0.205 | 12 | <.0001 | 0.0023 | 2.66 | UP |
| 45510 | PAQR5 | 1.409 | 0.308 | 12 | 0.0006 | 0.0181 | 2.66 | UP |
| 16630 | RHOQ | 1.409 | 0.291 | 12 | 0.0004 | 0.0146 | 2.66 | UP |
| 59098 | TBL1Y | 1.407 | 0.219 | 12 | <.0001 | 0.0035 | 2.65 | UP |
| 38771 | ALPK3 | 1.406 | 0.229 | 12 | <.0001 | 0.0045 | 2.65 | UP |
| 24591 | EMP2 | 1.403 | 0.161 | 12 | <.0001 | 0.0006 | 2.65 | UP |
| 8538 | YWHAH | 1.402 | 0.156 | 12 | <.0001 | 0.0006 | 2.64 | UP |
| 50266 | YWHAH | 1.396 | 0.180 | 12 | <.0001 | 0.0011 | 2.63 | UP |
| 51742 | CYFIP2 | 1.396 | 0.294 | 12 | 0.0005 | 0.0159 | 2.63 | UP |
| 21389 | LMTK2 | 1.390 | 0.289 | 12 | 0.0004 | 0.0152 | 2.62 | UP |
| 28849 | WHAMM | 1.387 | 0.305 | 12 | 0.0007 | 0.0188 | 2.61 | UP |
| 40089 | C12orf70 | 1.382 | 0.310 | 12 | 0.0008 | 0.0202 | 2.61 | UP |
| 40874 | YWHAH | 1.381 | 0.180 | 12 | <.0001 | 0.0012 | 2.60 | UP |
| 6477 | YWHAH | 1.379 | 0.158 | 12 | <.0001 | 0.0006 | 2.60 | UP |
| 44975 | PHF12 | 1.376 | 0.311 | 12 | 0.0008 | 0.0209 | 2.59 | UP |
| 48676 | YWHAH | 1.375 | 0.151 | 12 | <.0001 | 0.0006 | 2.59 | UP |
| 58751 | HS6ST1 | 1.374 | 0.306 | 12 | 0.0007 | 0.0197 | 2.59 | UP |
| 29913 | YWHAH | 1.374 | 0.186 | 12 | <.0001 | 0.0015 | 2.59 | UP |
| 46695 | RIMS3 | 1.373 | 0.240 | 12 | <.0001 | 0.0067 | 2.59 | UP |
| 49344 | CRLF1 | 1.369 | 0.311 | 12 | 0.0009 | 0.0213 | 2.58 | UP |
| 43916 | YPEL1 | 1.368 | 0.119 | 12 | <.0001 | 0.0002 | 2.58 | UP |
| 47597 | IQSEC1 | 1.361 | 0.168 | 12 | <.0001 | 0.0008 | 2.57 | UP |
| 44058 | POLR3E | 1.360 | 0.154 | 12 | <.0001 | 0.0006 | 2.57 | UP |
| 56140 | TFAP2A | 1.358 | 0.305 | 12 | 0.0008 | 0.0201 | 2.56 | UP |
| 12362 | FBXO11 | 1.357 | 0.288 | 12 | 0.0005 | 0.0161 | 2.56 | UP |
| 5323 | EPB41L1 | 1.357 | 0.243 | 12 | 0.0001 | 0.0076 | 2.56 | UP |
| 42970 | YWHAH | 1.354 | 0.154 | 12 | <.0001 | 0.0006 | 2.56 | UP |
| 36988 | EMP2 | 1.352 | 0.173 | 12 | <.0001 | 0.0011 | 2.55 | UP |
| 15082 | HERPUD2 | 1.350 | 0.189 | 12 | <.0001 | 0.0018 | 2.55 | UP |
| 51924 | SMO | 1.346 | 0.253 | 12 | 0.0002 | 0.0100 | 2.54 | UP |
| 16791 | TBL1Y | 1.346 | 0.143 | 12 | <.0001 | 0.0005 | 2.54 | UP |
| 36167 | CD14 | 1.342 | 0.267 | 12 | 0.0003 | 0.0126 | 2.53 | UP |
| 54461 | HOXA5 | 1.338 | 0.194 | 12 | <.0001 | 0.0022 | 2.53 | UP |
| 43724 | CRNDE | 1.337 | 0.281 | 12 | 0.0005 | 0.0158 | 2.53 | UP |
| 917 | YWHAH | 1.331 | 0.129 | 12 | <.0001 | 0.0003 | 2.52 | UP |
| 25400 | PXK | 1.329 | 0.218 | 12 | <.0001 | 0.0048 | 2.51 | UP |
| 41837 | CRNDE | 1.328 | 0.244 | 12 | 0.0001 | 0.0086 | 2.51 | UP |
| 37362 | SOX2 | 1.327 | 0.280 | 12 | 0.0005 | 0.0159 | 2.51 | UP |
| 17567 | XLOC_l2_0131 | 1.326 | 0.285 | 12 | 0.0006 | 0.0170 | 2.51 | UP |
| 4653 | XLOC_001265 | 1.325 | 0.150 | 12 | <.0001 | 0.0006 | 2.51 | UP |
| 50062 | SFT2D3 | 1.324 | 0.225 | 12 | <.0001 | 0.0059 | 2.50 | UP |
| 24018 | SCARNA3 | 1.320 | 0.294 | 12 | 0.0007 | 0.0196 | 2.50 | UP |
| 6497 | LSM14A | 1.319 | 0.191 | 12 | <.0001 | 0.0022 | 2.50 | UP |
| 14833 | PPAPDC2 | 1.318 | 0.249 | 12 | 0.0002 | 0.0102 | 2.49 | UP |
| 60486 | SEMA4F | 1.317 | 0.286 | 12 | 0.0006 | 0.0177 | 2.49 | UP |
| 22543 | TBL1Y | 1.315 | 0.143 | 12 | <.0001 | 0.0005 | 2.49 | UP |
| 27388 | ENST00000485 | 1.313 | 0.255 | 12 | 0.0002 | 0.0114 | 2.49 | UP |
| 3138 | WHAMM | 1.312 | 0.183 | 12 | <.0001 | 0.0018 | 2.48 | UP |
| 28611 | LOC153684 | 1.309 | 0.258 | 12 | 0.0003 | 0.0119 | 2.48 | UP |
| 36309 | C2orf15 | 1.308 | 0.291 | 12 | 0.0007 | 0.0195 | 2.48 | UP |
| 35799 | DNMT1 | 1.304 | 0.168 | 12 | <.0001 | 0.0011 | 2.47 | UP |
| 14735 | ZNF850 | 1.299 | 0.247 | 12 | 0.0002 | 0.0105 | 2.46 | UP |
| 18305 | YWHAH | 1.299 | 0.160 | 12 | <.0001 | 0.0008 | 2.46 | UP |
| 21051 | GPER | 1.298 | 0.266 | 12 | 0.0004 | 0.0143 | 2.46 | UP |
| 4021 | CMPK2 | 1.297 | 0.264 | 12 | 0.0004 | 0.0138 | 2.46 | UP |
| 33958 | LOC100509256 | 1.290 | 0.252 | 12 | 0.0003 | 0.0116 | 2.45 | UP |
| 21009 | SMAD6 | 1.288 | 0.254 | 12 | 0.0003 | 0.0120 | 2.44 | UP |
| 60830 | HIP1 | 1.285 | 0.220 | 12 | <.0001 | 0.0060 | 2.44 | UP |
| 37457 | SNX18 | 1.285 | 0.264 | 12 | 0.0004 | 0.0144 | 2.44 | UP |
| 37289 | TOP1MT | 1.279 | 0.289 | 12 | 0.0008 | 0.0208 | 2.43 | UP |
| 10353 | TRMT2B | 1.272 | 0.228 | 12 | 0.0001 | 0.0076 | 2.42 | UP |
| 50659 | IRF2BPL | 1.272 | 0.221 | 12 | <.0001 | 0.0067 | 2.42 | UP |
| 4177 | FLJ42627 | 1.271 | 0.197 | 12 | <.0001 | 0.0035 | 2.41 | UP |
| 37928 | CRNDE | 1.269 | 0.257 | 12 | 0.0003 | 0.0135 | 2.41 | UP |
| 19608 | ZNF319 | 1.268 | 0.269 | 12 | 0.0005 | 0.0162 | 2.41 | UP |
| 44499 | XLOC_002643 | 1.268 | 0.292 | 12 | 0.0010 | 0.0226 | 2.41 | UP |
| 62212 | POLR3E | 1.267 | 0.134 | 12 | <.0001 | 0.0005 | 2.41 | UP |
| 59046 | LINC00087 | 1.264 | 0.242 | 12 | 0.0002 | 0.0108 | 2.40 | UP |
| 3780 | LOC729870 | 1.264 | 0.158 | 12 | <.0001 | 0.0009 | 2.40 | UP |
| 57723 | AAK1 | 1.263 | 0.198 | 12 | <.0001 | 0.0036 | 2.40 | UP |
| 386 | FLJ42627 | 1.261 | 0.282 | 12 | 0.0008 | 0.0200 | 2.40 | UP |
| 49961 | AMIGO2 | 1.257 | 0.178 | 12 | <.0001 | 0.0019 | 2.39 | UP |
| 34366 | LOC100506251 | 1.254 | 0.249 | 12 | 0.0003 | 0.0125 | 2.38 | UP |
| 61705 | XLOC_l2_0020 | 1.248 | 0.177 | 12 | <.0001 | 0.0019 | 2.38 | UP |
| 27501 | TBL1X | 1.247 | 0.141 | 12 | <.0001 | 0.0006 | 2.37 | UP |
| 58491 | BC030764 | 1.247 | 0.157 | 12 | <.0001 | 0.0010 | 2.37 | UP |
| 55494 | C19orf23 | 1.244 | 0.275 | 12 | 0.0007 | 0.0191 | 2.37 | UP |
| 18978 | SNRNP27 | 1.240 | 0.169 | 12 | <.0001 | 0.0015 | 2.36 | UP |
| 49712 | MXI1 | 1.235 | 0.219 | 12 | 0.0001 | 0.0072 | 2.35 | UP |
| 28327 | CCDC149 | 1.232 | 0.215 | 12 | <.0001 | 0.0068 | 2.35 | UP |
| 26119 | LOC155060 | 1.230 | 0.194 | 12 | <.0001 | 0.0037 | 2.35 | UP |
| 57687 | SEPT6 | 1.230 | 0.236 | 12 | 0.0002 | 0.0108 | 2.35 | UP |
| 61963 | HOXA2 | 1.229 | 0.268 | 12 | 0.0006 | 0.0180 | 2.34 | UP |
| 61788 | PRKAR2B | 1.223 | 0.213 | 12 | <.0001 | 0.0067 | 2.33 | UP |
| 49522 | LOC100128191 | 1.221 | 0.238 | 12 | 0.0002 | 0.0116 | 2.33 | UP |
| 55274 | ATP9A | 1.220 | 0.236 | 12 | 0.0002 | 0.0112 | 2.33 | UP |
| 16427 | MEGF6 | 1.210 | 0.249 | 12 | 0.0004 | 0.0144 | 2.31 | UP |
| 30208 | CBLN3 | 1.209 | 0.203 | 12 | <.0001 | 0.0055 | 2.31 | UP |
| 43048 | RFX2 | 1.209 | 0.245 | 12 | 0.0003 | 0.0135 | 2.31 | UP |
| 3298 | FAM83E | 1.206 | 0.239 | 12 | 0.0003 | 0.0123 | 2.31 | UP |
| 17256 | TBL1Y | 1.206 | 0.202 | 12 | <.0001 | 0.0054 | 2.31 | UP |
| 36317 | LOC645586 | 1.206 | 0.225 | 12 | 0.0002 | 0.0095 | 2.31 | UP |
| 50161 | EGFR | 1.202 | 0.262 | 12 | 0.0006 | 0.0181 | 2.30 | UP |
| 5065 | BCL2L11 | 1.202 | 0.174 | 12 | <.0001 | 0.0022 | 2.30 | UP |
| 49241 | LPP-AS2 | 1.201 | 0.221 | 12 | 0.0002 | 0.0087 | 2.30 | UP |
| 35658 | LOC100507316 | 1.199 | 0.204 | 12 | <.0001 | 0.0060 | 2.30 | UP |
| 9827 | LOC100505679 | 1.198 | 0.240 | 12 | 0.0003 | 0.0128 | 2.29 | UP |
| 7274 | RALGPS1 | 1.198 | 0.226 | 12 | 0.0002 | 0.0102 | 2.29 | UP |
| 37071 | FBXO17 | 1.195 | 0.146 | 12 | <.0001 | 0.0008 | 2.29 | UP |
| 8048 | TBL1Y | 1.194 | 0.122 | 12 | <.0001 | 0.0004 | 2.29 | UP |
| 55927 | LBH | 1.193 | 0.219 | 12 | 0.0002 | 0.0087 | 2.29 | UP |
| 45710 | ANO10 | 1.189 | 0.077 | 12 | <.0001 | <.0001 | 2.28 | UP |
| 3447 | C17orf76 | 1.188 | 0.197 | 12 | <.0001 | 0.0052 | 2.28 | UP |
| 47474 | PLEKHM1 | 1.186 | 0.274 | 12 | 0.0010 | 0.0228 | 2.28 | UP |
| 51082 | GPC4 | 1.186 | 0.223 | 12 | 0.0002 | 0.0098 | 2.28 | UP |
| 30007 | MTHFR | 1.185 | 0.273 | 12 | 0.0010 | 0.0228 | 2.27 | UP |
| 23943 | CNKSR3 | 1.183 | 0.264 | 12 | 0.0008 | 0.0198 | 2.27 | UP |
| 16016 | KCNMB4 | 1.181 | 0.230 | 12 | 0.0002 | 0.0114 | 2.27 | UP |
| 48454 | ZFHX3 | 1.181 | 0.187 | 12 | <.0001 | 0.0038 | 2.27 | UP |
| 15656 | TMOD1 | 1.179 | 0.269 | 12 | 0.0009 | 0.0216 | 2.26 | UP |
| 51388 | LOC100652740 | 1.179 | 0.266 | 12 | 0.0008 | 0.0208 | 2.26 | UP |
| 44191 | IL20RB | 1.179 | 0.252 | 12 | 0.0005 | 0.0166 | 2.26 | UP |
| 52620 | BCOR | 1.179 | 0.267 | 12 | 0.0009 | 0.0213 | 2.26 | UP |
| 5352 | TBL1Y | 1.179 | 0.187 | 12 | <.0001 | 0.0039 | 2.26 | UP |
| 46829 | MTHFR | 1.178 | 0.256 | 12 | 0.0006 | 0.0179 | 2.26 | UP |
| 20011 | CDAN1 | 1.175 | 0.213 | 12 | 0.0001 | 0.0080 | 2.26 | UP |
| 41105 | MTHFR | 1.173 | 0.256 | 12 | 0.0006 | 0.0182 | 2.26 | UP |
| 25638 | XLOC_000822 | 1.173 | 0.240 | 12 | 0.0004 | 0.0142 | 2.26 | UP |
| 19111 | GSDMB | 1.170 | 0.237 | 12 | 0.0003 | 0.0136 | 2.25 | UP |
| 3986 | C17orf107 | 1.169 | 0.239 | 12 | 0.0004 | 0.0141 | 2.25 | UP |
| 57455 | KIAA1671 | 1.169 | 0.180 | 12 | <.0001 | 0.0034 | 2.25 | UP |
| 10106 | SNX27 | 1.165 | 0.198 | 12 | <.0001 | 0.0060 | 2.24 | UP |
| 12939 | MTHFR | 1.162 | 0.229 | 12 | 0.0003 | 0.0121 | 2.24 | UP |
| 25391 | DENND5B | 1.162 | 0.260 | 12 | 0.0008 | 0.0198 | 2.24 | UP |
| 8896 | NF1P2 | 1.158 | 0.268 | 12 | 0.0010 | 0.0229 | 2.23 | UP |
| 34168 | TSPAN10 | 1.157 | 0.267 | 12 | 0.0010 | 0.0228 | 2.23 | UP |
| 12715 | FBXO17 | 1.157 | 0.182 | 12 | <.0001 | 0.0037 | 2.23 | UP |
| 55989 | KLK7 | 1.156 | 0.262 | 12 | 0.0009 | 0.0213 | 2.23 | UP |
| 62655 | FHDC1 | 1.155 | 0.209 | 12 | 0.0001 | 0.0080 | 2.23 | UP |
| 22004 | FAAH2 | 1.154 | 0.264 | 12 | 0.0009 | 0.0222 | 2.23 | UP |
| 47325 | NFIB | 1.153 | 0.229 | 12 | 0.0003 | 0.0125 | 2.22 | UP |
| 28923 | LOC441268 | 1.151 | 0.235 | 12 | 0.0004 | 0.0141 | 2.22 | UP |
| 55971 | FBXO17 | 1.151 | 0.192 | 12 | <.0001 | 0.0052 | 2.22 | UP |
| 18879 | R3HDM1 | 1.150 | 0.252 | 12 | 0.0006 | 0.0184 | 2.22 | UP |
| 36036 | RHOQ | 1.149 | 0.169 | 12 | <.0001 | 0.0024 | 2.22 | UP |
| 27585 | MTHFR | 1.139 | 0.241 | 12 | 0.0005 | 0.0160 | 2.20 | UP |
| 14924 | FBXO17 | 1.131 | 0.188 | 12 | <.0001 | 0.0052 | 2.19 | UP |
| 43082 | MTHFR | 1.128 | 0.236 | 12 | 0.0004 | 0.0157 | 2.19 | UP |
| 40069 | TTC39C | 1.124 | 0.210 | 12 | 0.0002 | 0.0095 | 2.18 | UP |
| 23685 | THC2756939 | 1.124 | 0.198 | 12 | 0.0001 | 0.0070 | 2.18 | UP |
| 17543 | PNRC1 | 1.121 | 0.197 | 12 | 0.0001 | 0.0070 | 2.17 | UP |
| 18368 | XLOC_l2_0037 | 1.119 | 0.231 | 12 | 0.0004 | 0.0146 | 2.17 | UP |
| 25970 | AFF1 | 1.118 | 0.151 | 12 | <.0001 | 0.0015 | 2.17 | UP |
| 62862 | HS2ST1 | 1.118 | 0.249 | 12 | 0.0007 | 0.0195 | 2.17 | UP |
| 20966 | MLXIPL | 1.117 | 0.226 | 12 | 0.0003 | 0.0135 | 2.17 | UP |
| 23659 | CAMK2G | 1.117 | 0.254 | 12 | 0.0009 | 0.0215 | 2.17 | UP |
| 2740 | FBXO17 | 1.115 | 0.164 | 12 | <.0001 | 0.0024 | 2.17 | UP |
| 8362 | ZCCHC3 | 1.115 | 0.199 | 12 | 0.0001 | 0.0075 | 2.17 | UP |
| 59399 | MECP2 | 1.115 | 0.235 | 12 | 0.0005 | 0.0160 | 2.17 | UP |
| 35509 | NR2F1 | 1.112 | 0.238 | 12 | 0.0005 | 0.0167 | 2.16 | UP |
| 42317 | C16orf45 | 1.112 | 0.253 | 12 | 0.0009 | 0.0213 | 2.16 | UP |
| 25842 | ARID1B | 1.105 | 0.140 | 12 | <.0001 | 0.0010 | 2.15 | UP |
| 31296 | IFNAR2 | 1.104 | 0.232 | 12 | 0.0005 | 0.0158 | 2.15 | UP |
| 2780 | CMTM1 | 1.104 | 0.226 | 12 | 0.0004 | 0.0141 | 2.15 | UP |
| 48029 | SLCO3A1 | 1.104 | 0.231 | 12 | 0.0005 | 0.0158 | 2.15 | UP |
| 39038 | FBXO17 | 1.103 | 0.231 | 12 | 0.0005 | 0.0158 | 2.15 | UP |
| 20756 | AK124971 | 1.101 | 0.214 | 12 | 0.0002 | 0.0114 | 2.15 | UP |
| 51298 | SNTB1 | 1.099 | 0.246 | 12 | 0.0008 | 0.0198 | 2.14 | UP |
| 47048 | ZDHHC20 | 1.098 | 0.250 | 12 | 0.0009 | 0.0216 | 2.14 | UP |
| 17301 | VGLL4 | 1.096 | 0.195 | 12 | 0.0001 | 0.0074 | 2.14 | UP |
| 28528 | EVC | 1.096 | 0.197 | 12 | 0.0001 | 0.0078 | 2.14 | UP |
| 48539 | SSBP2 | 1.093 | 0.211 | 12 | 0.0002 | 0.0110 | 2.13 | UP |
| 15235 | CABLES2 | 1.087 | 0.248 | 12 | 0.0009 | 0.0219 | 2.12 | UP |
| 27644 | CYBRD1 | 1.085 | 0.225 | 12 | 0.0004 | 0.0149 | 2.12 | UP |
| 50086 | LOC100509205 | 1.080 | 0.194 | 12 | 0.0001 | 0.0077 | 2.11 | UP |
| 10 | LOC100506844 | 1.079 | 0.151 | 12 | <.0001 | 0.0018 | 2.11 | UP |
| 46816 | TBL1Y | 1.078 | 0.173 | 12 | <.0001 | 0.0041 | 2.11 | UP |
| 45649 | TMOD1 | 1.077 | 0.208 | 12 | 0.0002 | 0.0110 | 2.11 | UP |
| 24899 | ZNRF3 | 1.074 | 0.173 | 12 | <.0001 | 0.0043 | 2.11 | UP |
| 54103 | ALDH9A1 | 1.071 | 0.228 | 12 | 0.0005 | 0.0164 | 2.10 | UP |
| 42534 | QKI | 1.070 | 0.150 | 12 | <.0001 | 0.0018 | 2.10 | UP |
| 41187 | ILF3 | 1.068 | 0.153 | 12 | <.0001 | 0.0020 | 2.10 | UP |
| 49083 | HNRNPUL1 | 1.068 | 0.226 | 12 | 0.0005 | 0.0160 | 2.10 | UP |
| 41252 | LOC100506214 | 1.065 | 0.215 | 12 | 0.0003 | 0.0133 | 2.09 | UP |
| 11441 | FAM134A | 1.062 | 0.224 | 12 | 0.0005 | 0.0159 | 2.09 | UP |
| 60789 | ID2 | 1.058 | 0.130 | 12 | <.0001 | 0.0008 | 2.08 | UP |
| 23633 | TMOD1 | 1.058 | 0.179 | 12 | <.0001 | 0.0058 | 2.08 | UP |
| 5886 | UCK1 | 1.057 | 0.167 | 12 | <.0001 | 0.0039 | 2.08 | UP |
| 11493 | DEPTOR | 1.056 | 0.142 | 12 | <.0001 | 0.0015 | 2.08 | UP |
| 27558 | RELL1 | 1.055 | 0.120 | 12 | <.0001 | 0.0006 | 2.08 | UP |
| 9482 | TMEM209 | 1.048 | 0.219 | 12 | 0.0005 | 0.0158 | 2.07 | UP |
| 21053 | XLOC_l2_0132 | 1.047 | 0.169 | 12 | <.0001 | 0.0042 | 2.07 | UP |
| 25203 | IRS2 | 1.046 | 0.139 | 12 | <.0001 | 0.0013 | 2.06 | UP |
| 49412 | FAM123B | 1.042 | 0.203 | 12 | 0.0002 | 0.0116 | 2.06 | UP |
| 48925 | RBM20 | 1.036 | 0.178 | 12 | <.0001 | 0.0062 | 2.05 | UP |
| 53261 | ZNF592 | 1.036 | 0.223 | 12 | 0.0006 | 0.0170 | 2.05 | UP |
| 47497 | DKK3 | 1.032 | 0.176 | 12 | <.0001 | 0.0060 | 2.05 | UP |
| 31388 | WASF1 | 1.030 | 0.217 | 12 | 0.0005 | 0.0158 | 2.04 | UP |
| 35852 | FZD1 | 1.029 | 0.238 | 12 | 0.0010 | 0.0229 | 2.04 | UP |
| 50844 | PTPN3 | 1.027 | 0.152 | 12 | <.0001 | 0.0025 | 2.04 | UP |
| 3720 | PMP22 | 1.024 | 0.170 | 12 | <.0001 | 0.0052 | 2.03 | UP |
| 26023 | C1orf186 | 1.022 | 0.095 | 12 | <.0001 | 0.0002 | 2.03 | UP |
| 56704 | RBM33 | 1.021 | 0.233 | 12 | 0.0009 | 0.0218 | 2.03 | UP |
| 30884 | FBXO17 | 1.018 | 0.212 | 12 | 0.0004 | 0.0156 | 2.03 | UP |
| 26346 | EPHB2 | 1.017 | 0.223 | 12 | 0.0007 | 0.0185 | 2.02 | UP |
| 28815 | WHAMM | 1.016 | 0.190 | 12 | 0.0002 | 0.0095 | 2.02 | UP |
| 28411 | NPAS2 | 1.014 | 0.227 | 12 | 0.0008 | 0.0198 | 2.02 | UP |
| 15749 | KDM4D | 1.012 | 0.189 | 12 | 0.0002 | 0.0095 | 2.02 | UP |
| 16311 | OTX1 | 1.006 | 0.223 | 12 | 0.0007 | 0.0193 | 2.01 | UP |
| 11215 | TMOD1 | 1.003 | 0.183 | 12 | 0.0001 | 0.0084 | 2.00 | UP |
| 43545 | FAM24B | 1.002 | 0.212 | 12 | 0.0005 | 0.0160 | 2.00 | UP |
