# Supplementary material for: FOXM1 expression is significantly associated with chemotherapy resistance and adverse prognosis in non-serous epithelial ovarian cancer patients
Source: J Exp Clin Cancer Res. 2017 May 8;36:63. doi: 10.1186/s13046-017-0536-y (PMC5422964; doi:10.1186/s13046-017-0536-y)
Supplement: Supplementary file 9 — Figure S6: Volcano plot displaying differential expressed genes between siFOXM1 and siControl OVCAR-3 cells. Table S15: List of down-regulated genes in siFOXM1 OVCAR-3 cells. Table S16: List of up-regulated genes in siFOXM1 OVCAR-3 cells. (DOCX 345 kb) [file 13046_2017_536_MOESM9_ESM.docx]

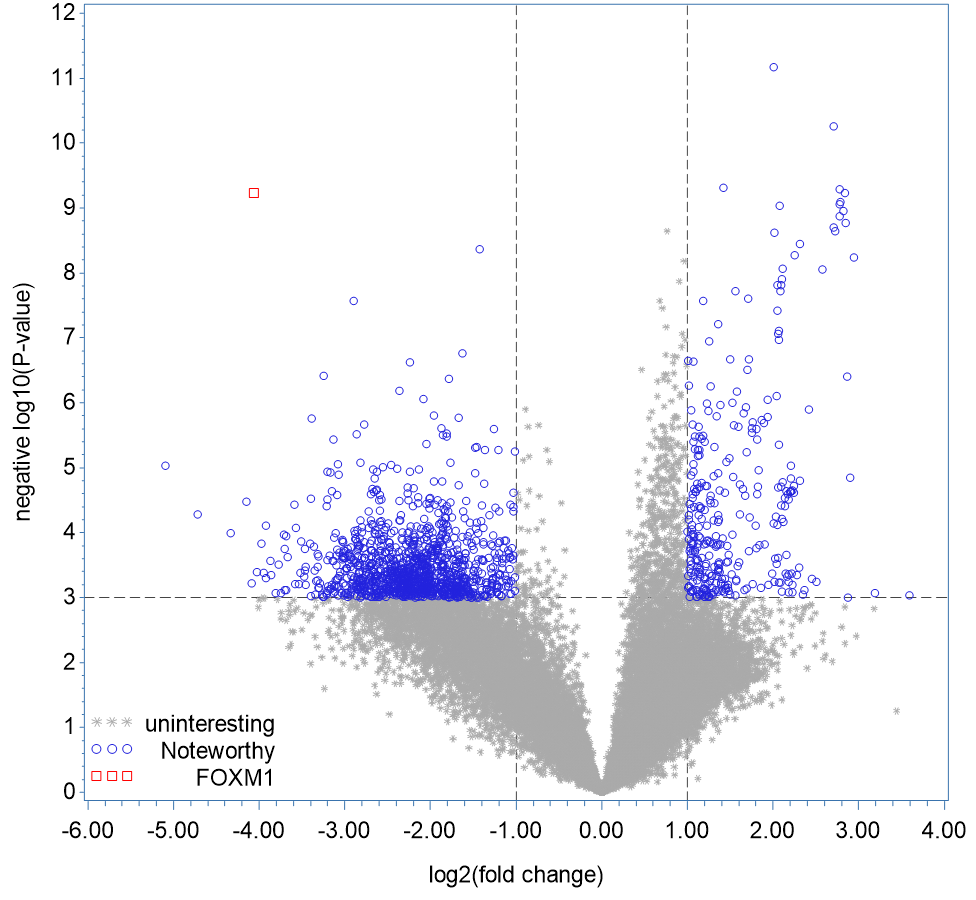


**Figure S6.** Volcano plot displaying differential expressed genes between siFOXM1 and siControl OVCAR-3 cells. The horizontal axis shows the base-2 logarithms of gene-expression fold changes for siFOXM1 cells compared to siControl cells. Vertical reference lines at +1.00 and –1.00 denote expression changes of 2 folds up and 2 folds down, respectively; data points outside these two vertical reference lines denote genes that show a >2-fold change in expression. The vertical axis shows the negative of the base-10 logarithm of the unadjusted P-values associated with expression changes. The horizontal line at 3 denotes unadjusted P=0.001; data points above this horizontal reference line thus have P<0.001 associated with their expression change. The red square denotes FOXM1. Blue circles denote non-FOXM1 genes that meet the double requirement of >2-fold change and P<0.001, whereas gray asterisks denote genes that do not meet the double requirement.

| **FeatureNum** | **GeneName** | **Estimate** | **Standard Error** | **DF** | **Pr > \|t\|** | **False Discovery Rate p-value** | **Fold Change** | **Direction of Change** |
| --- | --- | --- | --- | --- | --- | --- | --- | --- |
| 18370 | DYNC2H1 | -5.098 | 0.697 | 12 | <.0001 | 0.0010 | 34.26 | Down |
| 28435 | SCEL | -4.718 | 0.772 | 12 | <.0001 | 0.0029 | 26.32 | Down |
| 55831 | TNPO1 | -4.335 | 0.763 | 12 | 0.0001 | 0.0043 | 20.18 | Down |
| 25140 | NUPL1 | -4.150 | 0.647 | 12 | <.0001 | 0.0022 | 17.76 | Down |
| 17012 | TOP2A | -4.091 | 0.888 | 12 | 0.0006 | 0.0094 | 17.04 | Down |
| 55112 | FOXM1 | -4.058 | 0.229 | 12 | <.0001 | <.0001 | 16.66 | Down |
| 12604 | SUCLA2 | -4.029 | 0.832 | 12 | 0.0004 | 0.0081 | 16.33 | Down |
| 16447 | CPD | -3.975 | 0.729 | 12 | 0.0001 | 0.0052 | 15.73 | Down |
| 19407 | SUCLA2 | -3.952 | 0.819 | 12 | 0.0004 | 0.0082 | 15.47 | Down |
| 31402 | SUCLA2 | -3.925 | 0.670 | 12 | <.0001 | 0.0037 | 15.19 | Down |
| 14452 | SUCLA2 | -3.923 | 0.833 | 12 | 0.0005 | 0.0089 | 15.17 | Down |
| 49541 | SUCLA2 | -3.873 | 0.764 | 12 | 0.0003 | 0.0069 | 14.65 | Down |
| 58240 | PIKFYVE | -3.862 | 0.810 | 12 | 0.0005 | 0.0085 | 14.54 | Down |
| 36912 | USP34 | -3.808 | 0.863 | 12 | 0.0008 | 0.0104 | 14.01 | Down |
| 15486 | SUCLA2 | -3.777 | 0.756 | 12 | 0.0003 | 0.0073 | 13.71 | Down |
| 34221 | MYCBP2 | -3.743 | 0.850 | 12 | 0.0009 | 0.0104 | 13.39 | Down |
| 20434 | TLR3 | -3.712 | 0.657 | 12 | 0.0001 | 0.0044 | 13.11 | Down |
| 54800 | PSMD10 | -3.704 | 0.694 | 12 | 0.0002 | 0.0057 | 13.03 | Down |
| 25364 | NCOA3 | -3.702 | 0.828 | 12 | 0.0008 | 0.0100 | 13.01 | Down |
| 44639 | BOD1L | -3.690 | 0.827 | 12 | 0.0008 | 0.0101 | 12.90 | Down |
| 10040 | RAB27B | -3.688 | 0.656 | 12 | 0.0001 | 0.0046 | 12.88 | Down |
| 59548 | SUCLA2 | -3.669 | 0.711 | 12 | 0.0002 | 0.0066 | 12.72 | Down |
| 20052 | LOC643733 | -3.590 | 0.566 | 12 | <.0001 | 0.0024 | 12.05 | Down |
| 15625 | NHS | -3.579 | 0.781 | 12 | 0.0006 | 0.0094 | 11.95 | Down |
| 11535 | DCP2 | -3.575 | 0.617 | 12 | <.0001 | 0.0039 | 11.92 | Down |
| 60764 | ZNF148 | -3.538 | 0.770 | 12 | 0.0006 | 0.0094 | 11.61 | Down |
| 20881 | COG3 | -3.517 | 0.730 | 12 | 0.0004 | 0.0082 | 11.45 | Down |
| 44104 | IFIT2 | -3.513 | 0.639 | 12 | 0.0001 | 0.0051 | 11.42 | Down |
| 2491 | METAP2 | -3.472 | 0.731 | 12 | 0.0005 | 0.0086 | 11.10 | Down |
| 715 | CHD9 | -3.471 | 0.651 | 12 | 0.0002 | 0.0057 | 11.09 | Down |
| 29200 | OTUD4 | -3.467 | 0.712 | 12 | 0.0004 | 0.0080 | 11.06 | Down |
| 44914 | LNPEP | -3.461 | 0.700 | 12 | 0.0003 | 0.0075 | 11.01 | Down |
| 48957 | NCAPG | -3.460 | 0.755 | 12 | 0.0006 | 0.0095 | 11.01 | Down |
| 40772 | FAM190B | -3.407 | 0.625 | 12 | 0.0001 | 0.0052 | 10.61 | Down |
| 62747 | PKI55 | -3.396 | 0.524 | 12 | <.0001 | 0.0021 | 10.53 | Down |
| 30932 | INSIG2 | -3.393 | 0.781 | 12 | 0.0010 | 0.0108 | 10.51 | Down |
| 15709 | SPP1 | -3.384 | 0.393 | 12 | <.0001 | 0.0004 | 10.44 | Down |
| 40714 | ZNF614 | -3.357 | 0.623 | 12 | 0.0002 | 0.0055 | 10.25 | Down |
| 24967 | ZNF678 | -3.356 | 0.690 | 12 | 0.0004 | 0.0081 | 10.24 | Down |
| 57801 | CENPF | -3.344 | 0.765 | 12 | 0.0009 | 0.0107 | 10.16 | Down |
| 2595 | VCAN | -3.339 | 0.718 | 12 | 0.0006 | 0.0092 | 10.12 | Down |
| 10617 | SEL1L | -3.316 | 0.636 | 12 | 0.0002 | 0.0063 | 9.96 | Down |
| 14211 | KIAA0947 | -3.316 | 0.671 | 12 | 0.0003 | 0.0075 | 9.96 | Down |
| 58198 | ARHGAP21 | -3.316 | 0.711 | 12 | 0.0006 | 0.0091 | 9.96 | Down |
| 43015 | WDR36 | -3.311 | 0.734 | 12 | 0.0007 | 0.0098 | 9.93 | Down |
| 37329 | SCLT1 | -3.308 | 0.640 | 12 | 0.0002 | 0.0065 | 9.90 | Down |
| 20980 | PHF3 | -3.301 | 0.730 | 12 | 0.0007 | 0.0098 | 9.85 | Down |
| 45639 | DOCK7 | -3.289 | 0.713 | 12 | 0.0006 | 0.0093 | 9.77 | Down |
| 28757 | DCAF13 | -3.263 | 0.748 | 12 | 0.0009 | 0.0107 | 9.60 | Down |
| 26013 | RAD50 | -3.261 | 0.752 | 12 | 0.0010 | 0.0109 | 9.58 | Down |
| 51172 | GPSM2 | -3.250 | 0.327 | 12 | <.0001 | 0.0001 | 9.51 | Down |
| 16302 | VPS13C | -3.248 | 0.750 | 12 | 0.0010 | 0.0109 | 9.50 | Down |
| 14267 | MAP7D3 | -3.216 | 0.511 | 12 | <.0001 | 0.0024 | 9.30 | Down |
| 16684 | DNM1L | -3.215 | 0.734 | 12 | 0.0009 | 0.0106 | 9.28 | Down |
| 21894 | CCDC99 | -3.212 | 0.728 | 12 | 0.0009 | 0.0104 | 9.27 | Down |
| 10593 | ARL6IP1 | -3.207 | 0.496 | 12 | <.0001 | 0.0021 | 9.23 | Down |
| 39722 | SPP1 | -3.206 | 0.448 | 12 | <.0001 | 0.0012 | 9.23 | Down |
| 51810 | PLS3 | -3.206 | 0.630 | 12 | 0.0003 | 0.0068 | 9.23 | Down |
| 8373 | UTP14C | -3.204 | 0.720 | 12 | 0.0008 | 0.0102 | 9.22 | Down |
| 20496 | TTC14 | -3.192 | 0.702 | 12 | 0.0007 | 0.0097 | 9.14 | Down |
| 39830 | GPAM | -3.177 | 0.709 | 12 | 0.0007 | 0.0100 | 9.04 | Down |
| 15676 | TET2 | -3.174 | 0.611 | 12 | 0.0002 | 0.0064 | 9.03 | Down |
| 48056 | ZNF84 | -3.169 | 0.444 | 12 | <.0001 | 0.0012 | 8.99 | Down |
| 8224 | SELT | -3.162 | 0.703 | 12 | 0.0007 | 0.0099 | 8.95 | Down |
| 15291 | SPP1 | -3.148 | 0.472 | 12 | <.0001 | 0.0018 | 8.87 | Down |
| 51436 | LRPPRC | -3.147 | 0.688 | 12 | 0.0006 | 0.0095 | 8.86 | Down |
| 49577 | AQR | -3.143 | 0.619 | 12 | 0.0003 | 0.0069 | 8.83 | Down |
| 57268 | ARL6IP1 | -3.132 | 0.618 | 12 | 0.0003 | 0.0069 | 8.77 | Down |
| 20749 | USP24 | -3.132 | 0.712 | 12 | 0.0009 | 0.0104 | 8.77 | Down |
| 10428 | BANK1 | -3.132 | 0.390 | 12 | <.0001 | 0.0006 | 8.77 | Down |
| 40896 | KIDINS220 | -3.122 | 0.688 | 12 | 0.0007 | 0.0097 | 8.70 | Down |
| 46741 | KIF16B | -3.122 | 0.560 | 12 | 0.0001 | 0.0048 | 8.70 | Down |
| 12740 | USP25 | -3.117 | 0.703 | 12 | 0.0008 | 0.0103 | 8.67 | Down |
| 33446 | SLC30A9 | -3.111 | 0.666 | 12 | 0.0005 | 0.0090 | 8.64 | Down |
| 9599 | FAM133B | -3.111 | 0.683 | 12 | 0.0007 | 0.0097 | 8.64 | Down |
| 34952 | BBX | -3.110 | 0.548 | 12 | 0.0001 | 0.0043 | 8.63 | Down |
| 50998 | BBX | -3.099 | 0.598 | 12 | 0.0002 | 0.0064 | 8.57 | Down |
| 6836 | PPIP5K2 | -3.098 | 0.621 | 12 | 0.0003 | 0.0073 | 8.56 | Down |
| 56846 | SBF2 | -3.092 | 0.661 | 12 | 0.0005 | 0.0090 | 8.53 | Down |
| 30310 | KIF23 | -3.090 | 0.683 | 12 | 0.0007 | 0.0098 | 8.51 | Down |
| 30664 | FNIP1 | -3.089 | 0.581 | 12 | 0.0002 | 0.0058 | 8.51 | Down |
| 30337 | SPP1 | -3.087 | 0.470 | 12 | <.0001 | 0.0019 | 8.50 | Down |
| 60174 | DHX29 | -3.084 | 0.654 | 12 | 0.0005 | 0.0088 | 8.48 | Down |
| 46911 | KIAA1432 | -3.083 | 0.419 | 12 | <.0001 | 0.0010 | 8.47 | Down |
| 22983 | CAB39L | -3.080 | 0.707 | 12 | 0.0009 | 0.0108 | 8.45 | Down |
| 23292 | SPP1 | -3.075 | 0.434 | 12 | <.0001 | 0.0013 | 8.43 | Down |
| 22289 | PPIP5K2 | -3.068 | 0.675 | 12 | 0.0007 | 0.0097 | 8.39 | Down |
| 33852 | ZNF84 | -3.067 | 0.643 | 12 | 0.0005 | 0.0085 | 8.38 | Down |
| 61497 | CNOT8 | -3.063 | 0.583 | 12 | 0.0002 | 0.0060 | 8.36 | Down |
| 29514 | SMG1 | -3.052 | 0.630 | 12 | 0.0004 | 0.0081 | 8.30 | Down |
| 37073 | RAD50 | -3.036 | 0.688 | 12 | 0.0008 | 0.0104 | 8.20 | Down |
| 58362 | MACC1 | -3.033 | 0.548 | 12 | 0.0001 | 0.0050 | 8.18 | Down |
| 9548 | BOD1L | -3.032 | 0.595 | 12 | 0.0003 | 0.0068 | 8.18 | Down |
| 59955 | CKAP2 | -3.030 | 0.550 | 12 | 0.0001 | 0.0051 | 8.17 | Down |
| 58121 | BBX | -3.028 | 0.585 | 12 | 0.0002 | 0.0065 | 8.16 | Down |
| 18319 | XRCC4 | -3.022 | 0.656 | 12 | 0.0006 | 0.0093 | 8.12 | Down |
| 24508 | BBX | -3.018 | 0.584 | 12 | 0.0002 | 0.0065 | 8.10 | Down |
| 13765 | SMC6 | -3.018 | 0.575 | 12 | 0.0002 | 0.0061 | 8.10 | Down |
| 40374 | BBX | -3.017 | 0.550 | 12 | 0.0001 | 0.0051 | 8.10 | Down |
| 61965 | BBX | -3.016 | 0.586 | 12 | 0.0002 | 0.0066 | 8.09 | Down |
| 4843 | IARS | -3.012 | 0.568 | 12 | 0.0002 | 0.0059 | 8.07 | Down |
| 41763 | ARL6IP1 | -3.011 | 0.586 | 12 | 0.0002 | 0.0066 | 8.06 | Down |
| 54140 | ZNF84 | -3.010 | 0.689 | 12 | 0.0009 | 0.0107 | 8.06 | Down |
| 4038 | BBX | -3.000 | 0.615 | 12 | 0.0004 | 0.0080 | 8.00 | Down |
| 25118 | PTPN13 | -2.997 | 0.680 | 12 | 0.0009 | 0.0104 | 7.98 | Down |
| 15160 | BRWD1 | -2.994 | 0.582 | 12 | 0.0002 | 0.0066 | 7.96 | Down |
| 42319 | BBX | -2.993 | 0.566 | 12 | 0.0002 | 0.0059 | 7.96 | Down |
| 28979 | C5orf41 | -2.992 | 0.657 | 12 | 0.0007 | 0.0097 | 7.96 | Down |
| 54307 | PLS1 | -2.985 | 0.607 | 12 | 0.0004 | 0.0077 | 7.92 | Down |
| 10623 | GBP3 | -2.984 | 0.677 | 12 | 0.0008 | 0.0104 | 7.91 | Down |
| 47090 | TTK | -2.979 | 0.631 | 12 | 0.0005 | 0.0088 | 7.89 | Down |
| 19233 | KIAA2018 | -2.978 | 0.606 | 12 | 0.0004 | 0.0077 | 7.88 | Down |
| 19515 | SMCHD1 | -2.975 | 0.604 | 12 | 0.0004 | 0.0077 | 7.86 | Down |
| 16695 | MYCBP2 | -2.968 | 0.573 | 12 | 0.0002 | 0.0065 | 7.82 | Down |
| 3095 | PPIP5K2 | -2.968 | 0.674 | 12 | 0.0009 | 0.0104 | 7.82 | Down |
| 46208 | BBX | -2.966 | 0.556 | 12 | 0.0002 | 0.0057 | 7.81 | Down |
| 30341 | ARL6IP1 | -2.965 | 0.677 | 12 | 0.0009 | 0.0106 | 7.81 | Down |
| 17116 | XRCC4 | -2.965 | 0.684 | 12 | 0.0010 | 0.0109 | 7.81 | Down |
| 42861 | BBX | -2.954 | 0.564 | 12 | 0.0002 | 0.0062 | 7.75 | Down |
| 4514 | LRBA | -2.952 | 0.589 | 12 | 0.0003 | 0.0072 | 7.74 | Down |
| 59975 | NAMPT | -2.943 | 0.675 | 12 | 0.0009 | 0.0107 | 7.69 | Down |
| 35350 | PLCE1 | -2.941 | 0.566 | 12 | 0.0002 | 0.0064 | 7.68 | Down |
| 18045 | ANKRD32 | -2.934 | 0.592 | 12 | 0.0003 | 0.0075 | 7.64 | Down |
| 40415 | BIRC2 | -2.932 | 0.546 | 12 | 0.0002 | 0.0056 | 7.63 | Down |
| 61719 | ZNF84 | -2.931 | 0.573 | 12 | 0.0003 | 0.0067 | 7.62 | Down |
| 31246 | TAF2 | -2.928 | 0.662 | 12 | 0.0008 | 0.0104 | 7.61 | Down |
| 22849 | ZZZ3 | -2.927 | 0.567 | 12 | 0.0002 | 0.0066 | 7.61 | Down |
| 42390 | ZZZ3 | -2.924 | 0.615 | 12 | 0.0005 | 0.0086 | 7.59 | Down |
| 27080 | MNS1 | -2.922 | 0.636 | 12 | 0.0006 | 0.0094 | 7.58 | Down |
| 46819 | ARL6IP1 | -2.912 | 0.650 | 12 | 0.0008 | 0.0100 | 7.53 | Down |
| 22539 | ZZZ3 | -2.907 | 0.620 | 12 | 0.0005 | 0.0089 | 7.50 | Down |
| 11221 | DDX21 | -2.903 | 0.629 | 12 | 0.0006 | 0.0093 | 7.48 | Down |
| 61329 | TOPORS | -2.898 | 0.569 | 12 | 0.0003 | 0.0068 | 7.46 | Down |
| 54757 | ARL6IP1 | -2.898 | 0.641 | 12 | 0.0007 | 0.0098 | 7.45 | Down |
| 49859 | RPS6KC1 | -2.893 | 0.229 | 12 | <.0001 | <.0001 | 7.43 | Down |
| 39340 | PPIP5K2 | -2.893 | 0.637 | 12 | 0.0007 | 0.0097 | 7.43 | Down |
| 46632 | CFI | -2.890 | 0.575 | 12 | 0.0003 | 0.0071 | 7.41 | Down |
| 35635 | FRMD4B | -2.886 | 0.504 | 12 | <.0001 | 0.0042 | 7.39 | Down |
| 41915 | RLF | -2.884 | 0.610 | 12 | 0.0005 | 0.0087 | 7.38 | Down |
| 35645 | BORA | -2.881 | 0.508 | 12 | 0.0001 | 0.0044 | 7.37 | Down |
| 16030 | TROVE2 | -2.878 | 0.519 | 12 | 0.0001 | 0.0049 | 7.35 | Down |
| 17329 | SPP1 | -2.871 | 0.518 | 12 | 0.0001 | 0.0049 | 7.32 | Down |
| 55620 | TTC37 | -2.871 | 0.633 | 12 | 0.0007 | 0.0097 | 7.32 | Down |
| 50273 | UBR1 | -2.869 | 0.567 | 12 | 0.0003 | 0.0070 | 7.30 | Down |
| 8970 | HTATSF1 | -2.865 | 0.351 | 12 | <.0001 | 0.0006 | 7.29 | Down |
| 61388 | ZNF84 | -2.865 | 0.659 | 12 | 0.0009 | 0.0108 | 7.28 | Down |
| 58963 | PPIP5K2 | -2.863 | 0.625 | 12 | 0.0006 | 0.0094 | 7.28 | Down |
| 62008 | FNIP2 | -2.857 | 0.574 | 12 | 0.0003 | 0.0074 | 7.25 | Down |
| 19489 | IBTK | -2.856 | 0.522 | 12 | 0.0001 | 0.0052 | 7.24 | Down |
| 50163 | OPA1 | -2.856 | 0.609 | 12 | 0.0005 | 0.0089 | 7.24 | Down |
| 30138 | TFRC | -2.856 | 0.638 | 12 | 0.0008 | 0.0100 | 7.24 | Down |
| 56766 | SKIV2L2 | -2.855 | 0.497 | 12 | <.0001 | 0.0041 | 7.24 | Down |
| 43969 | PPP1R9A | -2.852 | 0.637 | 12 | 0.0008 | 0.0100 | 7.22 | Down |
| 17003 | BANK1 | -2.852 | 0.603 | 12 | 0.0005 | 0.0088 | 7.22 | Down |
| 24863 | HSPA8 | -2.851 | 0.538 | 12 | 0.0002 | 0.0059 | 7.22 | Down |
| 26147 | ACSL4 | -2.849 | 0.635 | 12 | 0.0007 | 0.0100 | 7.21 | Down |
| 1991 | EEF1A1 | -2.849 | 0.552 | 12 | 0.0002 | 0.0066 | 7.20 | Down |
| 9080 | PLCB4 | -2.848 | 0.572 | 12 | 0.0003 | 0.0074 | 7.20 | Down |
| 45359 | CNOT8 | -2.846 | 0.525 | 12 | 0.0002 | 0.0053 | 7.19 | Down |
| 35492 | IPO5 | -2.844 | 0.533 | 12 | 0.0002 | 0.0058 | 7.18 | Down |
| 7204 | CNOT8 | -2.838 | 0.587 | 12 | 0.0004 | 0.0081 | 7.15 | Down |
| 44540 | MAPK6 | -2.838 | 0.591 | 12 | 0.0004 | 0.0083 | 7.15 | Down |
| 28944 | ZBTB11 | -2.835 | 0.608 | 12 | 0.0006 | 0.0091 | 7.14 | Down |
| 59381 | SPP1 | -2.834 | 0.528 | 12 | 0.0002 | 0.0056 | 7.13 | Down |
| 3794 | BAZ1A | -2.831 | 0.508 | 12 | 0.0001 | 0.0048 | 7.11 | Down |
| 11382 | UTRN | -2.830 | 0.524 | 12 | 0.0002 | 0.0054 | 7.11 | Down |
| 35962 | ZBTB11 | -2.828 | 0.624 | 12 | 0.0007 | 0.0098 | 7.10 | Down |
| 26965 | NRIP1 | -2.827 | 0.585 | 12 | 0.0004 | 0.0082 | 7.10 | Down |
| 54595 | SNX13 | -2.827 | 0.567 | 12 | 0.0003 | 0.0073 | 7.10 | Down |
| 46849 | GPR87 | -2.824 | 0.562 | 12 | 0.0003 | 0.0072 | 7.08 | Down |
| 42733 | CEP192 | -2.823 | 0.606 | 12 | 0.0006 | 0.0092 | 7.08 | Down |
| 28903 | TFRC | -2.822 | 0.639 | 12 | 0.0008 | 0.0104 | 7.07 | Down |
| 20387 | TFRC | -2.822 | 0.625 | 12 | 0.0007 | 0.0098 | 7.07 | Down |
| 1492 | RAD54B | -2.822 | 0.472 | 12 | <.0001 | 0.0033 | 7.07 | Down |
| 29521 | CEP55 | -2.821 | 0.382 | 12 | <.0001 | 0.0010 | 7.07 | Down |
| 3224 | IFT74 | -2.821 | 0.653 | 12 | 0.0010 | 0.0111 | 7.07 | Down |
| 52171 | ZNF84 | -2.817 | 0.629 | 12 | 0.0008 | 0.0100 | 7.04 | Down |
| 30363 | POLR3B | -2.815 | 0.600 | 12 | 0.0005 | 0.0089 | 7.04 | Down |
| 61289 | ZBTB20 | -2.810 | 0.618 | 12 | 0.0007 | 0.0097 | 7.02 | Down |
| 55851 | AQR | -2.809 | 0.620 | 12 | 0.0007 | 0.0098 | 7.01 | Down |
| 42791 | MAP7D3 | -2.807 | 0.646 | 12 | 0.0010 | 0.0108 | 7.00 | Down |
| 41462 | KIAA1826 | -2.806 | 0.603 | 12 | 0.0006 | 0.0092 | 6.99 | Down |
| 16789 | WDFY3 | -2.804 | 0.609 | 12 | 0.0006 | 0.0094 | 6.98 | Down |
| 8391 | TFRC | -2.803 | 0.614 | 12 | 0.0006 | 0.0095 | 6.98 | Down |
| 35563 | PSMD10 | -2.800 | 0.545 | 12 | 0.0002 | 0.0066 | 6.96 | Down |
| 12442 | ZNF24 | -2.799 | 0.635 | 12 | 0.0009 | 0.0104 | 6.96 | Down |
| 32705 | VPS54 | -2.798 | 0.491 | 12 | <.0001 | 0.0043 | 6.96 | Down |
| 61911 | GTF3C3 | -2.797 | 0.480 | 12 | <.0001 | 0.0038 | 6.95 | Down |
| 1439 | SMC3 | -2.795 | 0.592 | 12 | 0.0005 | 0.0088 | 6.94 | Down |
| 17635 | PSME4 | -2.792 | 0.598 | 12 | 0.0005 | 0.0091 | 6.93 | Down |
| 6392 | TMCO7 | -2.792 | 0.574 | 12 | 0.0004 | 0.0081 | 6.92 | Down |
| 16055 | ARL6IP1 | -2.789 | 0.597 | 12 | 0.0005 | 0.0091 | 6.91 | Down |
| 48428 | IFIT1 | -2.779 | 0.520 | 12 | 0.0002 | 0.0057 | 6.86 | Down |
| 7135 | ARAP2 | -2.778 | 0.623 | 12 | 0.0008 | 0.0101 | 6.86 | Down |
| 25306 | SACS | -2.776 | 0.626 | 12 | 0.0008 | 0.0103 | 6.85 | Down |
| 22113 | SKIV2L2 | -2.776 | 0.506 | 12 | 0.0001 | 0.0051 | 6.85 | Down |
| 27194 | CDK19 | -2.774 | 0.329 | 12 | <.0001 | 0.0005 | 6.84 | Down |
| 10243 | ZNF267 | -2.773 | 0.609 | 12 | 0.0007 | 0.0097 | 6.83 | Down |
| 48523 | XRCC4 | -2.770 | 0.539 | 12 | 0.0002 | 0.0066 | 6.82 | Down |
| 54572 | CNOT8 | -2.766 | 0.560 | 12 | 0.0003 | 0.0076 | 6.80 | Down |
| 24788 | EXPH5 | -2.763 | 0.538 | 12 | 0.0002 | 0.0066 | 6.79 | Down |
| 8798 | AKAP12 | -2.763 | 0.554 | 12 | 0.0003 | 0.0073 | 6.79 | Down |
| 21682 | USP47 | -2.762 | 0.612 | 12 | 0.0007 | 0.0098 | 6.79 | Down |
| 41855 | USO1 | -2.760 | 0.633 | 12 | 0.0009 | 0.0107 | 6.77 | Down |
| 14780 | BAZ1A | -2.756 | 0.578 | 12 | 0.0005 | 0.0085 | 6.75 | Down |
| 52299 | UFL1 | -2.754 | 0.599 | 12 | 0.0006 | 0.0094 | 6.75 | Down |
| 58195 | NCKAP1 | -2.751 | 0.569 | 12 | 0.0004 | 0.0081 | 6.73 | Down |
| 29013 | SGTB | -2.749 | 0.526 | 12 | 0.0002 | 0.0062 | 6.72 | Down |
| 4295 | DCLRE1A | -2.749 | 0.627 | 12 | 0.0009 | 0.0106 | 6.72 | Down |
| 56750 | ATR | -2.746 | 0.628 | 12 | 0.0009 | 0.0107 | 6.71 | Down |
| 11970 | FANCM | -2.744 | 0.560 | 12 | 0.0004 | 0.0079 | 6.70 | Down |
| 34367 | ZBTB11 | -2.742 | 0.615 | 12 | 0.0008 | 0.0101 | 6.69 | Down |
| 295 | ZBTB11 | -2.726 | 0.589 | 12 | 0.0006 | 0.0093 | 6.62 | Down |
| 28472 | ZNF146 | -2.724 | 0.568 | 12 | 0.0004 | 0.0083 | 6.61 | Down |
| 11989 | TFRC | -2.722 | 0.626 | 12 | 0.0009 | 0.0108 | 6.60 | Down |
| 57087 | XRCC4 | -2.721 | 0.581 | 12 | 0.0005 | 0.0090 | 6.59 | Down |
| 12401 | PIK3CA | -2.719 | 0.440 | 12 | <.0001 | 0.0027 | 6.59 | Down |
| 19650 | MTIF2 | -2.718 | 0.469 | 12 | <.0001 | 0.0039 | 6.58 | Down |
| 33154 | EEF1A1 | -2.716 | 0.540 | 12 | 0.0003 | 0.0071 | 6.57 | Down |
| 55442 | ZZZ3 | -2.715 | 0.612 | 12 | 0.0008 | 0.0103 | 6.57 | Down |
| 61837 | GPR87 | -2.714 | 0.595 | 12 | 0.0007 | 0.0097 | 6.56 | Down |
| 62473 | PIK3CB | -2.714 | 0.548 | 12 | 0.0003 | 0.0075 | 6.56 | Down |
| 17579 | ZZZ3 | -2.709 | 0.602 | 12 | 0.0007 | 0.0099 | 6.54 | Down |
| 29275 | MBNL2 | -2.705 | 0.496 | 12 | 0.0001 | 0.0052 | 6.52 | Down |
| 57269 | CTR9 | -2.704 | 0.546 | 12 | 0.0003 | 0.0075 | 6.52 | Down |
| 13792 | KLHL28 | -2.699 | 0.510 | 12 | 0.0002 | 0.0059 | 6.49 | Down |
| 32788 | MUT | -2.699 | 0.452 | 12 | <.0001 | 0.0033 | 6.49 | Down |
| 61555 | CEP55 | -2.699 | 0.410 | 12 | <.0001 | 0.0019 | 6.49 | Down |
| 29464 | ENPP4 | -2.698 | 0.541 | 12 | 0.0003 | 0.0073 | 6.49 | Down |
| 38077 | PPIP5K2 | -2.697 | 0.590 | 12 | 0.0006 | 0.0095 | 6.49 | Down |
| 46542 | FAM208B | -2.694 | 0.567 | 12 | 0.0005 | 0.0086 | 6.47 | Down |
| 48769 | CENPF | -2.694 | 0.570 | 12 | 0.0005 | 0.0087 | 6.47 | Down |
| 62908 | PPIP5K2 | -2.693 | 0.622 | 12 | 0.0010 | 0.0110 | 6.47 | Down |
| 53087 | SKIV2L2 | -2.691 | 0.508 | 12 | 0.0002 | 0.0059 | 6.46 | Down |
| 7409 | RAD54B | -2.690 | 0.580 | 12 | 0.0006 | 0.0092 | 6.45 | Down |
| 37952 | BAZ1A | -2.688 | 0.536 | 12 | 0.0003 | 0.0072 | 6.44 | Down |
| 12110 | MFN1 | -2.686 | 0.456 | 12 | <.0001 | 0.0036 | 6.44 | Down |
| 19115 | RNF219 | -2.686 | 0.519 | 12 | 0.0002 | 0.0065 | 6.43 | Down |
| 43759 | MYO5A | -2.684 | 0.486 | 12 | 0.0001 | 0.0050 | 6.43 | Down |
| 5713 | CNOT8 | -2.684 | 0.620 | 12 | 0.0010 | 0.0110 | 6.43 | Down |
| 16790 | ZNF17 | -2.684 | 0.507 | 12 | 0.0002 | 0.0059 | 6.43 | Down |
| 6289 | CEP55 | -2.680 | 0.399 | 12 | <.0001 | 0.0017 | 6.41 | Down |
| 50942 | MYO9A | -2.677 | 0.533 | 12 | 0.0003 | 0.0071 | 6.40 | Down |
| 49897 | CEBPZ | -2.674 | 0.550 | 12 | 0.0004 | 0.0080 | 6.38 | Down |
| 8106 | WDFY1 | -2.674 | 0.574 | 12 | 0.0006 | 0.0092 | 6.38 | Down |
| 16835 | CEBPZ | -2.674 | 0.575 | 12 | 0.0006 | 0.0092 | 6.38 | Down |
| 61574 | CWC22 | -2.671 | 0.455 | 12 | <.0001 | 0.0036 | 6.37 | Down |
| 55397 | CEP55 | -2.667 | 0.370 | 12 | <.0001 | 0.0012 | 6.35 | Down |
| 12468 | CEP55 | -2.664 | 0.400 | 12 | <.0001 | 0.0018 | 6.34 | Down |
| 57315 | CEBPZ | -2.663 | 0.551 | 12 | 0.0004 | 0.0082 | 6.33 | Down |
| 56790 | ERCC8 | -2.657 | 0.542 | 12 | 0.0004 | 0.0078 | 6.31 | Down |
| 10426 | SKIV2L2 | -2.654 | 0.505 | 12 | 0.0002 | 0.0061 | 6.30 | Down |
| 50259 | ZNF146 | -2.653 | 0.560 | 12 | 0.0005 | 0.0087 | 6.29 | Down |
| 15245 | RAD54B | -2.653 | 0.586 | 12 | 0.0007 | 0.0098 | 6.29 | Down |
| 13096 | CEP55 | -2.652 | 0.380 | 12 | <.0001 | 0.0014 | 6.29 | Down |
| 5201 | CNOT8 | -2.652 | 0.583 | 12 | 0.0007 | 0.0097 | 6.29 | Down |
| 7169 | ZBTB11 | -2.652 | 0.590 | 12 | 0.0007 | 0.0100 | 6.29 | Down |
| 59980 | TFRC | -2.650 | 0.613 | 12 | 0.0010 | 0.0110 | 6.28 | Down |
| 14985 | LARP7 | -2.649 | 0.605 | 12 | 0.0009 | 0.0106 | 6.27 | Down |
| 35717 | SKIV2L2 | -2.649 | 0.466 | 12 | 0.0001 | 0.0043 | 6.27 | Down |
| 14721 | MPDZ | -2.648 | 0.564 | 12 | 0.0005 | 0.0089 | 6.27 | Down |
| 16316 | MCMBP | -2.647 | 0.467 | 12 | 0.0001 | 0.0044 | 6.26 | Down |
| 16592 | ZFHX4 | -2.644 | 0.459 | 12 | <.0001 | 0.0040 | 6.25 | Down |
| 39582 | EPRS | -2.643 | 0.550 | 12 | 0.0004 | 0.0083 | 6.25 | Down |
| 58354 | SEPT7 | -2.642 | 0.568 | 12 | 0.0006 | 0.0092 | 6.24 | Down |
| 43868 | CEP63 | -2.641 | 0.561 | 12 | 0.0005 | 0.0089 | 6.24 | Down |
| 14356 | BANK1 | -2.638 | 0.608 | 12 | 0.0010 | 0.0109 | 6.23 | Down |
| 30566 | ERP27 | -2.637 | 0.554 | 12 | 0.0005 | 0.0086 | 6.22 | Down |
| 50300 | KIAA1826 | -2.636 | 0.580 | 12 | 0.0007 | 0.0097 | 6.22 | Down |
| 42115 | CEP55 | -2.636 | 0.395 | 12 | <.0001 | 0.0018 | 6.21 | Down |
| 869 | PSMC6 | -2.633 | 0.591 | 12 | 0.0008 | 0.0101 | 6.20 | Down |
| 36652 | ZZZ3 | -2.632 | 0.602 | 12 | 0.0009 | 0.0107 | 6.20 | Down |
| 21565 | ZNF33A | -2.632 | 0.511 | 12 | 0.0002 | 0.0066 | 6.20 | Down |
| 23637 | GDAP2 | -2.630 | 0.368 | 12 | <.0001 | 0.0012 | 6.19 | Down |
| 58758 | CLIP4 | -2.628 | 0.587 | 12 | 0.0008 | 0.0100 | 6.18 | Down |
| 3239 | ZBTB11 | -2.628 | 0.596 | 12 | 0.0008 | 0.0104 | 6.18 | Down |
| 50818 | NOL11 | -2.625 | 0.594 | 12 | 0.0008 | 0.0104 | 6.17 | Down |
| 61155 | RAD54B | -2.623 | 0.566 | 12 | 0.0006 | 0.0092 | 6.16 | Down |
| 3512 | CEP55 | -2.623 | 0.391 | 12 | <.0001 | 0.0017 | 6.16 | Down |
| 16517 | SKIV2L2 | -2.617 | 0.513 | 12 | 0.0003 | 0.0068 | 6.13 | Down |
| 12063 | ZNF518A | -2.616 | 0.593 | 12 | 0.0008 | 0.0104 | 6.13 | Down |
| 40908 | SKIV2L2 | -2.615 | 0.549 | 12 | 0.0005 | 0.0085 | 6.13 | Down |
| 62796 | TRAK2 | -2.615 | 0.550 | 12 | 0.0005 | 0.0086 | 6.12 | Down |
| 39151 | DLD | -2.613 | 0.548 | 12 | 0.0005 | 0.0085 | 6.12 | Down |
| 10665 | HNRNPH2 | -2.613 | 0.503 | 12 | 0.0002 | 0.0064 | 6.12 | Down |
| 61698 | CEBPZ | -2.611 | 0.596 | 12 | 0.0009 | 0.0106 | 6.11 | Down |
| 47642 | RND3 | -2.610 | 0.475 | 12 | 0.0001 | 0.0051 | 6.10 | Down |
| 46638 | DDX58 | -2.609 | 0.567 | 12 | 0.0006 | 0.0094 | 6.10 | Down |
| 16014 | SMC6 | -2.607 | 0.601 | 12 | 0.0010 | 0.0109 | 6.09 | Down |
| 55625 | ANKRD5 | -2.605 | 0.537 | 12 | 0.0004 | 0.0081 | 6.09 | Down |
| 44598 | CEP55 | -2.601 | 0.404 | 12 | <.0001 | 0.0021 | 6.07 | Down |
| 12203 | BAZ1A | -2.600 | 0.516 | 12 | 0.0003 | 0.0071 | 6.06 | Down |
| 25010 | ZNF146 | -2.599 | 0.601 | 12 | 0.0010 | 0.0110 | 6.06 | Down |
| 59392 | HECTD1 | -2.599 | 0.458 | 12 | 0.0001 | 0.0044 | 6.06 | Down |
| 15970 | CEBPZ | -2.592 | 0.580 | 12 | 0.0008 | 0.0101 | 6.03 | Down |
| 2187 | SETDB2 | -2.591 | 0.437 | 12 | <.0001 | 0.0034 | 6.03 | Down |
| 1388 | KDM4C | -2.591 | 0.418 | 12 | <.0001 | 0.0027 | 6.03 | Down |
| 20498 | SPP1 | -2.590 | 0.489 | 12 | 0.0002 | 0.0059 | 6.02 | Down |
| 5368 | AKAP12 | -2.589 | 0.510 | 12 | 0.0003 | 0.0069 | 6.02 | Down |
| 13497 | CEP55 | -2.588 | 0.393 | 12 | <.0001 | 0.0019 | 6.01 | Down |
| 62851 | MYNN | -2.587 | 0.553 | 12 | 0.0005 | 0.0090 | 6.01 | Down |
| 18418 | ERCC5 | -2.586 | 0.528 | 12 | 0.0004 | 0.0078 | 6.00 | Down |
| 25460 | HMGCR | -2.584 | 0.479 | 12 | 0.0002 | 0.0054 | 6.00 | Down |
| 56739 | CHD1 | -2.584 | 0.578 | 12 | 0.0008 | 0.0101 | 6.00 | Down |
| 44546 | SKIV2L2 | -2.582 | 0.488 | 12 | 0.0002 | 0.0059 | 5.99 | Down |
| 51217 | ZNF146 | -2.582 | 0.592 | 12 | 0.0009 | 0.0107 | 5.99 | Down |
| 42552 | C12orf4 | -2.578 | 0.529 | 12 | 0.0004 | 0.0080 | 5.97 | Down |
| 57499 | CDK6 | -2.577 | 0.440 | 12 | <.0001 | 0.0037 | 5.97 | Down |
| 64 | SSFA2 | -2.575 | 0.433 | 12 | <.0001 | 0.0034 | 5.96 | Down |
| 10735 | SNX13 | -2.575 | 0.465 | 12 | 0.0001 | 0.0050 | 5.96 | Down |
| 62593 | FAS | -2.573 | 0.545 | 12 | 0.0005 | 0.0088 | 5.95 | Down |
| 1447 | ERCC5 | -2.573 | 0.464 | 12 | 0.0001 | 0.0049 | 5.95 | Down |
| 40354 | CEP55 | -2.573 | 0.398 | 12 | <.0001 | 0.0021 | 5.95 | Down |
| 32354 | IFT81 | -2.561 | 0.581 | 12 | 0.0009 | 0.0104 | 5.90 | Down |
| 25438 | WRN | -2.560 | 0.587 | 12 | 0.0009 | 0.0107 | 5.90 | Down |
| 55714 | SRD5A1 | -2.558 | 0.352 | 12 | <.0001 | 0.0011 | 5.89 | Down |
| 87 | HSP90AA1 | -2.558 | 0.543 | 12 | 0.0005 | 0.0089 | 5.89 | Down |
| 15672 | POLR2B | -2.555 | 0.554 | 12 | 0.0006 | 0.0093 | 5.88 | Down |
| 4598 | ZNF732 | -2.555 | 0.505 | 12 | 0.0003 | 0.0070 | 5.88 | Down |
| 55351 | RPAP3 | -2.553 | 0.578 | 12 | 0.0008 | 0.0104 | 5.87 | Down |
| 31657 | CENPQ | -2.551 | 0.460 | 12 | 0.0001 | 0.0049 | 5.86 | Down |
| 11248 | MECOM | -2.549 | 0.454 | 12 | 0.0001 | 0.0046 | 5.85 | Down |
| 47720 | GAPVD1 | -2.549 | 0.510 | 12 | 0.0003 | 0.0073 | 5.85 | Down |
| 16395 | CEBPZ | -2.548 | 0.566 | 12 | 0.0007 | 0.0099 | 5.85 | Down |
| 43556 | A_33_P326495 | -2.540 | 0.442 | 12 | <.0001 | 0.0041 | 5.82 | Down |
| 61241 | UTP20 | -2.540 | 0.534 | 12 | 0.0005 | 0.0085 | 5.82 | Down |
| 50571 | HNRNPH2 | -2.532 | 0.562 | 12 | 0.0007 | 0.0099 | 5.78 | Down |
| 22126 | RND3 | -2.530 | 0.480 | 12 | 0.0002 | 0.0060 | 5.78 | Down |
| 41504 | KDM4C | -2.529 | 0.522 | 12 | 0.0004 | 0.0081 | 5.77 | Down |
| 7847 | RAD54B | -2.525 | 0.570 | 12 | 0.0008 | 0.0103 | 5.76 | Down |
| 50101 | IPO7 | -2.524 | 0.565 | 12 | 0.0008 | 0.0100 | 5.75 | Down |
| 20333 | ITGAV | -2.524 | 0.560 | 12 | 0.0007 | 0.0098 | 5.75 | Down |
| 39755 | FAM208A | -2.523 | 0.537 | 12 | 0.0005 | 0.0089 | 5.75 | Down |
| 55267 | TUG1 | -2.523 | 0.583 | 12 | 0.0010 | 0.0110 | 5.75 | Down |
| 20850 | HNRNPH2 | -2.523 | 0.479 | 12 | 0.0002 | 0.0060 | 5.75 | Down |
| 33744 | HEATR1 | -2.517 | 0.433 | 12 | <.0001 | 0.0038 | 5.72 | Down |
| 53979 | ABCE1 | -2.517 | 0.494 | 12 | 0.0003 | 0.0068 | 5.72 | Down |
| 20040 | RAB3GAP2 | -2.516 | 0.519 | 12 | 0.0004 | 0.0081 | 5.72 | Down |
| 11547 | PIK3R4 | -2.514 | 0.557 | 12 | 0.0007 | 0.0098 | 5.71 | Down |
| 7488 | C11orf82 | -2.513 | 0.548 | 12 | 0.0006 | 0.0094 | 5.71 | Down |
| 37195 | C1orf9 | -2.511 | 0.535 | 12 | 0.0005 | 0.0089 | 5.70 | Down |
| 42696 | PNRC2 | -2.510 | 0.525 | 12 | 0.0004 | 0.0084 | 5.69 | Down |
| 40087 | ZNF561 | -2.502 | 0.553 | 12 | 0.0007 | 0.0098 | 5.67 | Down |
| 61605 | TMTC2 | -2.500 | 0.490 | 12 | 0.0003 | 0.0068 | 5.66 | Down |
| 1189 | HSP90AB2P | -2.498 | 0.540 | 12 | 0.0006 | 0.0093 | 5.65 | Down |
| 16994 | FXR1 | -2.493 | 0.422 | 12 | <.0001 | 0.0035 | 5.63 | Down |
| 16660 | HNRNPH2 | -2.491 | 0.517 | 12 | 0.0004 | 0.0082 | 5.62 | Down |
| 29066 | UPF3B | -2.490 | 0.562 | 12 | 0.0008 | 0.0103 | 5.62 | Down |
| 28590 | TNPO1 | -2.489 | 0.554 | 12 | 0.0007 | 0.0100 | 5.61 | Down |
| 57539 | WDR3 | -2.488 | 0.453 | 12 | 0.0001 | 0.0051 | 5.61 | Down |
| 16859 | RND3 | -2.488 | 0.471 | 12 | 0.0002 | 0.0060 | 5.61 | Down |
| 48198 | ITGAV | -2.487 | 0.554 | 12 | 0.0007 | 0.0100 | 5.61 | Down |
| 7028 | RAD54B | -2.486 | 0.557 | 12 | 0.0008 | 0.0101 | 5.60 | Down |
| 19758 | SSFA2 | -2.484 | 0.485 | 12 | 0.0003 | 0.0067 | 5.59 | Down |
| 56749 | HNRNPH2 | -2.483 | 0.528 | 12 | 0.0005 | 0.0089 | 5.59 | Down |
| 43997 | ANKRD17 | -2.482 | 0.529 | 12 | 0.0005 | 0.0089 | 5.59 | Down |
| 46023 | RND3 | -2.481 | 0.462 | 12 | 0.0002 | 0.0055 | 5.58 | Down |
| 12117 | RND3 | -2.481 | 0.454 | 12 | 0.0001 | 0.0052 | 5.58 | Down |
| 47392 | ITGAV | -2.480 | 0.558 | 12 | 0.0008 | 0.0102 | 5.58 | Down |
| 54564 | FXR1 | -2.478 | 0.462 | 12 | 0.0002 | 0.0056 | 5.57 | Down |
| 55710 | ITGAV | -2.474 | 0.563 | 12 | 0.0009 | 0.0105 | 5.55 | Down |
| 45979 | MARCH7 | -2.472 | 0.505 | 12 | 0.0004 | 0.0079 | 5.55 | Down |
| 18218 | SKIV2L2 | -2.472 | 0.494 | 12 | 0.0003 | 0.0073 | 5.55 | Down |
| 24642 | CNOT8 | -2.471 | 0.514 | 12 | 0.0004 | 0.0083 | 5.54 | Down |
| 10862 | KIAA1826 | -2.469 | 0.552 | 12 | 0.0008 | 0.0100 | 5.53 | Down |
| 52520 | RAPGEF2 | -2.465 | 0.506 | 12 | 0.0004 | 0.0080 | 5.52 | Down |
| 62082 | ZSCAN29 | -2.465 | 0.544 | 12 | 0.0007 | 0.0098 | 5.52 | Down |
| 7875 | MKI67 | -2.463 | 0.433 | 12 | 0.0001 | 0.0043 | 5.51 | Down |
| 21062 | RND3 | -2.463 | 0.493 | 12 | 0.0003 | 0.0073 | 5.51 | Down |
| 46180 | KIAA1429 | -2.463 | 0.478 | 12 | 0.0002 | 0.0066 | 5.51 | Down |
| 15438 | SSX2IP | -2.462 | 0.507 | 12 | 0.0004 | 0.0081 | 5.51 | Down |
| 53723 | ZNF268 | -2.459 | 0.531 | 12 | 0.0006 | 0.0093 | 5.50 | Down |
| 38776 | SMC2 | -2.458 | 0.517 | 12 | 0.0005 | 0.0086 | 5.49 | Down |
| 11054 | UNC13B | -2.457 | 0.335 | 12 | <.0001 | 0.0010 | 5.49 | Down |
| 24291 | MATR3 | -2.454 | 0.497 | 12 | 0.0003 | 0.0075 | 5.48 | Down |
| 53704 | RBBP8 | -2.451 | 0.503 | 12 | 0.0004 | 0.0080 | 5.47 | Down |
| 13082 | ITGAV | -2.448 | 0.551 | 12 | 0.0008 | 0.0102 | 5.46 | Down |
| 37541 | HNRNPH2 | -2.445 | 0.507 | 12 | 0.0004 | 0.0082 | 5.44 | Down |
| 2040 | KDM4C | -2.445 | 0.521 | 12 | 0.0005 | 0.0089 | 5.44 | Down |
| 7504 | DCLRE1C | -2.443 | 0.480 | 12 | 0.0003 | 0.0068 | 5.44 | Down |
| 52627 | RND3 | -2.441 | 0.464 | 12 | 0.0002 | 0.0060 | 5.43 | Down |
| 54600 | ERCC5 | -2.436 | 0.513 | 12 | 0.0005 | 0.0086 | 5.41 | Down |
| 11014 | PRKDC | -2.436 | 0.542 | 12 | 0.0007 | 0.0100 | 5.41 | Down |
| 4123 | CENPQ | -2.433 | 0.555 | 12 | 0.0009 | 0.0106 | 5.40 | Down |
| 20142 | ZNF845 | -2.430 | 0.548 | 12 | 0.0008 | 0.0103 | 5.39 | Down |
| 8910 | TOM1L1 | -2.427 | 0.547 | 12 | 0.0008 | 0.0103 | 5.38 | Down |
| 17124 | SMARCA5 | -2.426 | 0.531 | 12 | 0.0006 | 0.0096 | 5.37 | Down |
| 8688 | DHX57 | -2.425 | 0.520 | 12 | 0.0005 | 0.0091 | 5.37 | Down |
| 50005 | LRPPRC | -2.422 | 0.477 | 12 | 0.0003 | 0.0069 | 5.36 | Down |
| 32240 | C1orf9 | -2.422 | 0.497 | 12 | 0.0004 | 0.0080 | 5.36 | Down |
| 30564 | POLR3G | -2.422 | 0.423 | 12 | <.0001 | 0.0041 | 5.36 | Down |
| 45186 | EXO1 | -2.421 | 0.513 | 12 | 0.0005 | 0.0088 | 5.35 | Down |
| 49373 | DOCK4 | -2.417 | 0.548 | 12 | 0.0008 | 0.0104 | 5.34 | Down |
| 41419 | UTP14A | -2.417 | 0.488 | 12 | 0.0003 | 0.0075 | 5.34 | Down |
| 55500 | SLC38A1 | -2.416 | 0.499 | 12 | 0.0004 | 0.0081 | 5.34 | Down |
| 16243 | ERCC5 | -2.416 | 0.527 | 12 | 0.0006 | 0.0094 | 5.34 | Down |
| 18437 | WDHD1 | -2.416 | 0.487 | 12 | 0.0003 | 0.0075 | 5.34 | Down |
| 24532 | KIAA0368 | -2.414 | 0.527 | 12 | 0.0006 | 0.0094 | 5.33 | Down |
| 18648 | MIA3 | -2.411 | 0.545 | 12 | 0.0008 | 0.0103 | 5.32 | Down |
| 20764 | IARS | -2.410 | 0.483 | 12 | 0.0003 | 0.0073 | 5.31 | Down |
| 55631 | FAS | -2.406 | 0.526 | 12 | 0.0006 | 0.0095 | 5.30 | Down |
| 53322 | PCM1 | -2.404 | 0.512 | 12 | 0.0005 | 0.0089 | 5.29 | Down |
| 11452 | SSFA2 | -2.403 | 0.448 | 12 | 0.0002 | 0.0056 | 5.29 | Down |
| 39452 | KIAA1826 | -2.400 | 0.550 | 12 | 0.0009 | 0.0107 | 5.28 | Down |
| 21115 | PRKD3 | -2.395 | 0.502 | 12 | 0.0005 | 0.0085 | 5.26 | Down |
| 29478 | HSP90B1 | -2.395 | 0.493 | 12 | 0.0004 | 0.0081 | 5.26 | Down |
| 47506 | C1orf9 | -2.393 | 0.500 | 12 | 0.0004 | 0.0084 | 5.25 | Down |
| 57393 | RND3 | -2.392 | 0.467 | 12 | 0.0003 | 0.0067 | 5.25 | Down |
| 1735 | C1orf9 | -2.392 | 0.514 | 12 | 0.0006 | 0.0092 | 5.25 | Down |
| 10844 | CCDC99 | -2.391 | 0.494 | 12 | 0.0004 | 0.0081 | 5.25 | Down |
| 53249 | NMD3 | -2.390 | 0.541 | 12 | 0.0008 | 0.0104 | 5.24 | Down |
| 24629 | SLC38A1 | -2.388 | 0.513 | 12 | 0.0006 | 0.0092 | 5.23 | Down |
| 18390 | MKI67 | -2.386 | 0.330 | 12 | <.0001 | 0.0011 | 5.23 | Down |
| 8222 | VPS35 | -2.386 | 0.533 | 12 | 0.0008 | 0.0100 | 5.23 | Down |
| 50022 | USP16 | -2.383 | 0.513 | 12 | 0.0006 | 0.0092 | 5.22 | Down |
| 1551 | LRRC49 | -2.382 | 0.521 | 12 | 0.0006 | 0.0095 | 5.21 | Down |
| 21879 | CCDC99 | -2.382 | 0.494 | 12 | 0.0004 | 0.0082 | 5.21 | Down |
| 13232 | OTUD6B | -2.379 | 0.530 | 12 | 0.0007 | 0.0100 | 5.20 | Down |
| 20326 | HNRNPH2 | -2.373 | 0.505 | 12 | 0.0005 | 0.0089 | 5.18 | Down |
| 45546 | GDAP2 | -2.372 | 0.450 | 12 | 0.0002 | 0.0060 | 5.18 | Down |
| 18842 | COL12A1 | -2.370 | 0.510 | 12 | 0.0006 | 0.0092 | 5.17 | Down |
| 56822 | RND3 | -2.368 | 0.486 | 12 | 0.0004 | 0.0080 | 5.16 | Down |
| 303 | CCDC99 | -2.368 | 0.496 | 12 | 0.0005 | 0.0085 | 5.16 | Down |
| 34844 | XLOC_l2_0027 | -2.367 | 0.396 | 12 | <.0001 | 0.0033 | 5.16 | Down |
| 23154 | EXO1 | -2.365 | 0.526 | 12 | 0.0007 | 0.0099 | 5.15 | Down |
| 23798 | CLASP2 | -2.365 | 0.540 | 12 | 0.0009 | 0.0106 | 5.15 | Down |
| 58613 | HNRNPH2 | -2.363 | 0.536 | 12 | 0.0009 | 0.0104 | 5.15 | Down |
| 47074 | POC1B | -2.363 | 0.250 | 12 | <.0001 | 0.0002 | 5.14 | Down |
| 55661 | FBXO38 | -2.362 | 0.485 | 12 | 0.0004 | 0.0080 | 5.14 | Down |
| 12775 | ZNF30 | -2.361 | 0.368 | 12 | <.0001 | 0.0022 | 5.14 | Down |
| 4616 | SCFD1 | -2.356 | 0.518 | 12 | 0.0007 | 0.0097 | 5.12 | Down |
| 37916 | LEO1 | -2.355 | 0.477 | 12 | 0.0003 | 0.0076 | 5.11 | Down |
| 36466 | WDR7 | -2.354 | 0.458 | 12 | 0.0002 | 0.0066 | 5.11 | Down |
| 24930 | XLOC_l2_0027 | -2.353 | 0.394 | 12 | <.0001 | 0.0033 | 5.11 | Down |
| 55879 | EIF3J | -2.353 | 0.487 | 12 | 0.0004 | 0.0082 | 5.11 | Down |
| 13752 | STXBP3 | -2.351 | 0.531 | 12 | 0.0008 | 0.0103 | 5.10 | Down |
| 58342 | ERCC5 | -2.351 | 0.504 | 12 | 0.0005 | 0.0091 | 5.10 | Down |
| 1369 | KDM4C | -2.350 | 0.510 | 12 | 0.0006 | 0.0093 | 5.10 | Down |
| 46681 | ZNF271 | -2.350 | 0.539 | 12 | 0.0009 | 0.0107 | 5.10 | Down |
| 20599 | NBPF10 | -2.348 | 0.385 | 12 | <.0001 | 0.0030 | 5.09 | Down |
| 32493 | ORC3 | -2.347 | 0.511 | 12 | 0.0006 | 0.0094 | 5.09 | Down |
| 20611 | WDHD1 | -2.345 | 0.519 | 12 | 0.0007 | 0.0098 | 5.08 | Down |
| 36334 | PIK3C3 | -2.344 | 0.500 | 12 | 0.0005 | 0.0089 | 5.08 | Down |
| 62225 | ZNF613 | -2.343 | 0.499 | 12 | 0.0005 | 0.0089 | 5.07 | Down |
| 4647 | WDHD1 | -2.343 | 0.479 | 12 | 0.0004 | 0.0079 | 5.07 | Down |
| 22176 | C14orf129 | -2.342 | 0.471 | 12 | 0.0003 | 0.0074 | 5.07 | Down |
| 50254 | C1orf9 | -2.342 | 0.493 | 12 | 0.0005 | 0.0086 | 5.07 | Down |
| 9674 | KIF23 | -2.339 | 0.441 | 12 | 0.0002 | 0.0059 | 5.06 | Down |
| 48346 | WDHD1 | -2.339 | 0.476 | 12 | 0.0004 | 0.0078 | 5.06 | Down |
| 62105 | ZKSCAN1 | -2.339 | 0.518 | 12 | 0.0007 | 0.0098 | 5.06 | Down |
| 52590 | NEK2 | -2.336 | 0.420 | 12 | 0.0001 | 0.0049 | 5.05 | Down |
| 20468 | TOM1L1 | -2.336 | 0.512 | 12 | 0.0007 | 0.0097 | 5.05 | Down |
| 1719 | BCAP29 | -2.335 | 0.529 | 12 | 0.0008 | 0.0104 | 5.04 | Down |
| 62745 | ITGB1 | -2.332 | 0.410 | 12 | 0.0001 | 0.0043 | 5.03 | Down |
| 9354 | IFT80 | -2.332 | 0.421 | 12 | 0.0001 | 0.0050 | 5.03 | Down |
| 22099 | SSFA2 | -2.326 | 0.528 | 12 | 0.0009 | 0.0104 | 5.02 | Down |
| 46568 | WDHD1 | -2.325 | 0.461 | 12 | 0.0003 | 0.0071 | 5.01 | Down |
| 22154 | RALGAPB | -2.323 | 0.502 | 12 | 0.0006 | 0.0093 | 5.00 | Down |
| 46963 | RND3 | -2.323 | 0.481 | 12 | 0.0004 | 0.0082 | 5.00 | Down |
| 56340 | C1orf9 | -2.320 | 0.498 | 12 | 0.0005 | 0.0091 | 4.99 | Down |
| 50675 | LOC100652752 | -2.320 | 0.461 | 12 | 0.0003 | 0.0071 | 4.99 | Down |
| 34531 | SSFA2 | -2.320 | 0.480 | 12 | 0.0004 | 0.0082 | 4.99 | Down |
| 15857 | C1orf9 | -2.317 | 0.532 | 12 | 0.0009 | 0.0108 | 4.98 | Down |
| 50436 | EXO1 | -2.316 | 0.534 | 12 | 0.0010 | 0.0109 | 4.98 | Down |
| 47050 | CCDC47 | -2.316 | 0.512 | 12 | 0.0007 | 0.0098 | 4.98 | Down |
| 37768 | RPS6KC1 | -2.316 | 0.502 | 12 | 0.0006 | 0.0093 | 4.98 | Down |
| 25560 | EXO1 | -2.315 | 0.498 | 12 | 0.0006 | 0.0092 | 4.98 | Down |
| 40419 | TOM1L1 | -2.315 | 0.510 | 12 | 0.0007 | 0.0097 | 4.98 | Down |
| 41369 | UBE4A | -2.313 | 0.486 | 12 | 0.0005 | 0.0085 | 4.97 | Down |
| 19770 | WDHD1 | -2.313 | 0.493 | 12 | 0.0005 | 0.0089 | 4.97 | Down |
| 34750 | ZC3H15 | -2.312 | 0.477 | 12 | 0.0004 | 0.0081 | 4.96 | Down |
| 58795 | TOM1L1 | -2.311 | 0.508 | 12 | 0.0007 | 0.0097 | 4.96 | Down |
| 25148 | SSFA2 | -2.311 | 0.504 | 12 | 0.0006 | 0.0094 | 4.96 | Down |
| 14531 | USP46 | -2.311 | 0.481 | 12 | 0.0004 | 0.0083 | 4.96 | Down |
| 52350 | ERCC5 | -2.310 | 0.506 | 12 | 0.0006 | 0.0096 | 4.96 | Down |
| 15878 | RAD54B | -2.310 | 0.470 | 12 | 0.0004 | 0.0078 | 4.96 | Down |
| 58055 | KIAA1033 | -2.310 | 0.527 | 12 | 0.0009 | 0.0106 | 4.96 | Down |
| 42509 | CCDC99 | -2.308 | 0.507 | 12 | 0.0007 | 0.0097 | 4.95 | Down |
| 41198 | CCDC99 | -2.304 | 0.506 | 12 | 0.0007 | 0.0097 | 4.94 | Down |
| 2092 | PHKB | -2.303 | 0.501 | 12 | 0.0006 | 0.0094 | 4.94 | Down |
| 31274 | OFD1 | -2.301 | 0.489 | 12 | 0.0005 | 0.0089 | 4.93 | Down |
| 40330 | A_32_P53558 | -2.300 | 0.487 | 12 | 0.0005 | 0.0088 | 4.92 | Down |
| 46185 | ANK3 | -2.294 | 0.524 | 12 | 0.0009 | 0.0106 | 4.90 | Down |
| 58958 | ITGB1 | -2.294 | 0.438 | 12 | 0.0002 | 0.0061 | 4.90 | Down |
| 37724 | TMTC3 | -2.291 | 0.528 | 12 | 0.0010 | 0.0109 | 4.89 | Down |
| 46737 | KDM4C | -2.290 | 0.526 | 12 | 0.0009 | 0.0108 | 4.89 | Down |
| 11251 | TUG1 | -2.290 | 0.525 | 12 | 0.0009 | 0.0107 | 4.89 | Down |
| 55496 | ITGB1 | -2.285 | 0.421 | 12 | 0.0002 | 0.0053 | 4.87 | Down |
| 41409 | EDEM3 | -2.282 | 0.492 | 12 | 0.0006 | 0.0092 | 4.86 | Down |
| 28628 | LIMA1 | -2.282 | 0.470 | 12 | 0.0004 | 0.0081 | 4.86 | Down |
| 36100 | MPP5 | -2.280 | 0.443 | 12 | 0.0002 | 0.0066 | 4.86 | Down |
| 51699 | RPS6KC1 | -2.277 | 0.477 | 12 | 0.0005 | 0.0085 | 4.85 | Down |
| 30592 | EIF2S2 | -2.274 | 0.515 | 12 | 0.0008 | 0.0104 | 4.84 | Down |
| 11682 | C1orf9 | -2.272 | 0.519 | 12 | 0.0009 | 0.0106 | 4.83 | Down |
| 59507 | GOLGA5 | -2.272 | 0.448 | 12 | 0.0003 | 0.0069 | 4.83 | Down |
| 51488 | PSME4 | -2.272 | 0.500 | 12 | 0.0007 | 0.0097 | 4.83 | Down |
| 36336 | KIN | -2.268 | 0.496 | 12 | 0.0006 | 0.0095 | 4.82 | Down |
| 30469 | BBS10 | -2.268 | 0.499 | 12 | 0.0007 | 0.0097 | 4.82 | Down |
| 4914 | PLCE1 | -2.268 | 0.463 | 12 | 0.0004 | 0.0078 | 4.82 | Down |
| 11904 | KDM4C | -2.268 | 0.479 | 12 | 0.0005 | 0.0087 | 4.82 | Down |
| 11839 | CDK5RAP2 | -2.267 | 0.493 | 12 | 0.0006 | 0.0094 | 4.81 | Down |
| 57332 | SSFA2 | -2.267 | 0.502 | 12 | 0.0007 | 0.0098 | 4.81 | Down |
| 42240 | EPHA4 | -2.267 | 0.343 | 12 | <.0001 | 0.0019 | 4.81 | Down |
| 45244 | TOM1L1 | -2.267 | 0.518 | 12 | 0.0009 | 0.0107 | 4.81 | Down |
| 13881 | GTF2H1 | -2.266 | 0.409 | 12 | 0.0001 | 0.0050 | 4.81 | Down |
| 2512 | EPHA4 | -2.266 | 0.335 | 12 | <.0001 | 0.0017 | 4.81 | Down |
| 4658 | WDHD1 | -2.266 | 0.513 | 12 | 0.0008 | 0.0104 | 4.81 | Down |
| 50909 | STAT1 | -2.265 | 0.466 | 12 | 0.0004 | 0.0081 | 4.81 | Down |
| 480 | C1orf9 | -2.263 | 0.493 | 12 | 0.0006 | 0.0094 | 4.80 | Down |
| 52458 | ERC1 | -2.263 | 0.398 | 12 | 0.0001 | 0.0043 | 4.80 | Down |
| 52693 | SECISBP2 | -2.262 | 0.500 | 12 | 0.0007 | 0.0098 | 4.80 | Down |
| 45433 | NOC3L | -2.262 | 0.429 | 12 | 0.0002 | 0.0060 | 4.80 | Down |
| 56136 | EPHA4 | -2.262 | 0.351 | 12 | <.0001 | 0.0021 | 4.80 | Down |
| 56257 | KDM4C | -2.261 | 0.493 | 12 | 0.0006 | 0.0094 | 4.79 | Down |
| 24390 | SOS2 | -2.260 | 0.466 | 12 | 0.0004 | 0.0081 | 4.79 | Down |
| 46972 | RPS6KC1 | -2.259 | 0.496 | 12 | 0.0007 | 0.0097 | 4.79 | Down |
| 29362 | MAP4K4 | -2.257 | 0.451 | 12 | 0.0003 | 0.0073 | 4.78 | Down |
| 41655 | DNAJA1 | -2.256 | 0.505 | 12 | 0.0008 | 0.0101 | 4.78 | Down |
| 38116 | RPS6KA3 | -2.253 | 0.484 | 12 | 0.0006 | 0.0092 | 4.77 | Down |
| 34463 | PARP4 | -2.253 | 0.470 | 12 | 0.0004 | 0.0084 | 4.77 | Down |
| 31623 | EPHA4 | -2.252 | 0.394 | 12 | <.0001 | 0.0042 | 4.76 | Down |
| 1290 | NME7 | -2.252 | 0.516 | 12 | 0.0009 | 0.0107 | 4.76 | Down |
| 22850 | STAT1 | -2.252 | 0.430 | 12 | 0.0002 | 0.0062 | 4.76 | Down |
| 3157 | ITGB1 | -2.250 | 0.467 | 12 | 0.0004 | 0.0082 | 4.76 | Down |
| 51071 | ITGB1 | -2.250 | 0.476 | 12 | 0.0005 | 0.0087 | 4.76 | Down |
| 5134 | LAMA3 | -2.249 | 0.495 | 12 | 0.0007 | 0.0097 | 4.75 | Down |
| 32089 | NUP133 | -2.247 | 0.480 | 12 | 0.0005 | 0.0089 | 4.75 | Down |
| 26846 | PREPL | -2.246 | 0.457 | 12 | 0.0004 | 0.0077 | 4.74 | Down |
| 44696 | MTR | -2.246 | 0.408 | 12 | 0.0001 | 0.0051 | 4.74 | Down |
| 11626 | MAP3K5 | -2.244 | 0.486 | 12 | 0.0006 | 0.0093 | 4.74 | Down |
| 3040 | ZCCHC9 | -2.243 | 0.313 | 12 | <.0001 | 0.0012 | 4.73 | Down |
| 13522 | OSBPL11 | -2.242 | 0.456 | 12 | 0.0004 | 0.0077 | 4.73 | Down |
| 24824 | ARHGAP5 | -2.240 | 0.441 | 12 | 0.0003 | 0.0069 | 4.73 | Down |
| 28009 | MED8 | -2.240 | 0.453 | 12 | 0.0003 | 0.0075 | 4.72 | Down |
| 54536 | KDM4C | -2.240 | 0.458 | 12 | 0.0004 | 0.0079 | 4.72 | Down |
| 829 | TBK1 | -2.238 | 0.409 | 12 | 0.0001 | 0.0052 | 4.72 | Down |
| 2130 | DICER1 | -2.237 | 0.498 | 12 | 0.0007 | 0.0100 | 4.71 | Down |
| 11834 | IL1B | -2.237 | 0.215 | 12 | <.0001 | 0.0001 | 4.71 | Down |
| 62033 | RRM2B | -2.234 | 0.501 | 12 | 0.0008 | 0.0101 | 4.70 | Down |
| 32925 | AHNAK | -2.233 | 0.382 | 12 | <.0001 | 0.0037 | 4.70 | Down |
| 19803 | WDHD1 | -2.233 | 0.501 | 12 | 0.0008 | 0.0101 | 4.70 | Down |
| 24594 | CCDC99 | -2.229 | 0.509 | 12 | 0.0009 | 0.0107 | 4.69 | Down |
| 14370 | EFR3A | -2.227 | 0.492 | 12 | 0.0007 | 0.0098 | 4.68 | Down |
| 62736 | ZNF350 | -2.227 | 0.511 | 12 | 0.0009 | 0.0108 | 4.68 | Down |
| 62780 | ARFGEF1 | -2.227 | 0.481 | 12 | 0.0006 | 0.0093 | 4.68 | Down |
| 18704 | TOP2B | -2.226 | 0.472 | 12 | 0.0005 | 0.0088 | 4.68 | Down |
| 45144 | GDAP2 | -2.226 | 0.418 | 12 | 0.0002 | 0.0058 | 4.68 | Down |
| 55357 | KDM4C | -2.224 | 0.484 | 12 | 0.0006 | 0.0094 | 4.67 | Down |
| 46270 | TOP2B | -2.222 | 0.500 | 12 | 0.0008 | 0.0102 | 4.67 | Down |
| 61795 | CKAP5 | -2.222 | 0.496 | 12 | 0.0008 | 0.0100 | 4.67 | Down |
| 60929 | PRRC2C | -2.222 | 0.495 | 12 | 0.0007 | 0.0100 | 4.66 | Down |
| 38160 | UBR2 | -2.221 | 0.466 | 12 | 0.0005 | 0.0085 | 4.66 | Down |
| 1496 | TOP2B | -2.221 | 0.478 | 12 | 0.0006 | 0.0092 | 4.66 | Down |
| 1404 | USP48 | -2.220 | 0.481 | 12 | 0.0006 | 0.0093 | 4.66 | Down |
| 23200 | STAT1 | -2.214 | 0.408 | 12 | 0.0002 | 0.0053 | 4.64 | Down |
| 1062 | DNAJC13 | -2.213 | 0.462 | 12 | 0.0004 | 0.0084 | 4.64 | Down |
| 20827 | SF3B1 | -2.212 | 0.506 | 12 | 0.0009 | 0.0107 | 4.63 | Down |
| 57264 | SSFA2 | -2.210 | 0.491 | 12 | 0.0007 | 0.0099 | 4.63 | Down |
| 47469 | KDM4C | -2.208 | 0.450 | 12 | 0.0004 | 0.0078 | 4.62 | Down |
| 36775 | CCDC99 | -2.208 | 0.496 | 12 | 0.0008 | 0.0101 | 4.62 | Down |
| 18767 | NOC3L | -2.208 | 0.439 | 12 | 0.0003 | 0.0071 | 4.62 | Down |
| 33121 | SSFA2 | -2.208 | 0.427 | 12 | 0.0002 | 0.0065 | 4.62 | Down |
| 20670 | CTNNA1 | -2.208 | 0.496 | 12 | 0.0008 | 0.0102 | 4.62 | Down |
| 27939 | TOP2B | -2.206 | 0.469 | 12 | 0.0005 | 0.0089 | 4.61 | Down |
| 20154 | RIOK2 | -2.206 | 0.408 | 12 | 0.0002 | 0.0054 | 4.61 | Down |
| 10315 | TOP2B | -2.204 | 0.467 | 12 | 0.0005 | 0.0088 | 4.61 | Down |
| 11406 | EPHA4 | -2.202 | 0.350 | 12 | <.0001 | 0.0024 | 4.60 | Down |
| 1175 | TSC1 | -2.200 | 0.405 | 12 | 0.0002 | 0.0053 | 4.60 | Down |
| 11358 | FAT1 | -2.200 | 0.390 | 12 | 0.0001 | 0.0045 | 4.60 | Down |
| 33985 | BLM | -2.200 | 0.487 | 12 | 0.0007 | 0.0098 | 4.59 | Down |
| 30991 | DCDC2 | -2.198 | 0.497 | 12 | 0.0008 | 0.0103 | 4.59 | Down |
| 31530 | ITGB1 | -2.197 | 0.461 | 12 | 0.0005 | 0.0085 | 4.59 | Down |
| 45974 | WAPAL | -2.197 | 0.457 | 12 | 0.0004 | 0.0083 | 4.58 | Down |
| 28078 | EIF4G3 | -2.196 | 0.326 | 12 | <.0001 | 0.0017 | 4.58 | Down |
| 12499 | NOC3L | -2.195 | 0.433 | 12 | 0.0003 | 0.0069 | 4.58 | Down |
| 38359 | MFAP1 | -2.195 | 0.398 | 12 | 0.0001 | 0.0050 | 4.58 | Down |
| 56853 | CLIP1 | -2.193 | 0.471 | 12 | 0.0006 | 0.0091 | 4.57 | Down |
| 57055 | GDAP2 | -2.191 | 0.497 | 12 | 0.0008 | 0.0104 | 4.57 | Down |
| 43234 | TOP2B | -2.189 | 0.466 | 12 | 0.0005 | 0.0089 | 4.56 | Down |
| 10863 | LMO7 | -2.188 | 0.396 | 12 | 0.0001 | 0.0050 | 4.56 | Down |
| 42061 | NOC3L | -2.187 | 0.450 | 12 | 0.0004 | 0.0081 | 4.55 | Down |
| 51916 | GFPT1 | -2.186 | 0.500 | 12 | 0.0009 | 0.0107 | 4.55 | Down |
| 39355 | PGM2L1 | -2.185 | 0.429 | 12 | 0.0003 | 0.0068 | 4.55 | Down |
| 8915 | RIOK2 | -2.185 | 0.459 | 12 | 0.0005 | 0.0085 | 4.55 | Down |
| 16318 | KIF3B | -2.181 | 0.390 | 12 | 0.0001 | 0.0047 | 4.53 | Down |
| 55997 | ARHGEF3 | -2.180 | 0.456 | 12 | 0.0004 | 0.0084 | 4.53 | Down |
| 44875 | IMPA1 | -2.179 | 0.505 | 12 | 0.0010 | 0.0111 | 4.53 | Down |
| 55058 | BPTF | -2.178 | 0.424 | 12 | 0.0002 | 0.0066 | 4.52 | Down |
| 4065 | PLEKHA5 | -2.177 | 0.492 | 12 | 0.0008 | 0.0103 | 4.52 | Down |
| 14684 | ZNF26 | -2.177 | 0.483 | 12 | 0.0007 | 0.0099 | 4.52 | Down |
| 9537 | GBP3 | -2.176 | 0.442 | 12 | 0.0004 | 0.0077 | 4.52 | Down |
| 55635 | WWP1 | -2.176 | 0.414 | 12 | 0.0002 | 0.0061 | 4.52 | Down |
| 12987 | TOP2B | -2.174 | 0.482 | 12 | 0.0007 | 0.0098 | 4.51 | Down |
| 51723 | NOC3L | -2.170 | 0.423 | 12 | 0.0002 | 0.0066 | 4.50 | Down |
| 18378 | POC5 | -2.168 | 0.499 | 12 | 0.0010 | 0.0109 | 4.49 | Down |
| 40088 | OCLN | -2.167 | 0.467 | 12 | 0.0006 | 0.0092 | 4.49 | Down |
| 58233 | TRIP12 | -2.166 | 0.372 | 12 | <.0001 | 0.0038 | 4.49 | Down |
| 16980 | ENOX2 | -2.164 | 0.414 | 12 | 0.0002 | 0.0062 | 4.48 | Down |
| 45793 | UBR5 | -2.164 | 0.422 | 12 | 0.0002 | 0.0066 | 4.48 | Down |
| 16936 | NOC3L | -2.161 | 0.416 | 12 | 0.0002 | 0.0064 | 4.47 | Down |
| 47592 | DDX1 | -2.161 | 0.420 | 12 | 0.0002 | 0.0066 | 4.47 | Down |
| 15105 | CGRRF1 | -2.158 | 0.450 | 12 | 0.0004 | 0.0084 | 4.46 | Down |
| 58593 | MIOS | -2.157 | 0.488 | 12 | 0.0008 | 0.0104 | 4.46 | Down |
| 14059 | CCDC93 | -2.157 | 0.491 | 12 | 0.0009 | 0.0105 | 4.46 | Down |
| 61748 | GDAP2 | -2.155 | 0.456 | 12 | 0.0005 | 0.0088 | 4.45 | Down |
| 53363 | NOC3L | -2.152 | 0.436 | 12 | 0.0003 | 0.0076 | 4.45 | Down |
| 16682 | PNRC2 | -2.152 | 0.418 | 12 | 0.0002 | 0.0066 | 4.44 | Down |
| 44990 | ZW10 | -2.148 | 0.432 | 12 | 0.0003 | 0.0074 | 4.43 | Down |
| 46773 | MAK16 | -2.147 | 0.481 | 12 | 0.0008 | 0.0101 | 4.43 | Down |
| 25545 | KIAA0196 | -2.147 | 0.429 | 12 | 0.0003 | 0.0073 | 4.43 | Down |
| 21210 | ITGB1 | -2.146 | 0.472 | 12 | 0.0007 | 0.0097 | 4.42 | Down |
| 8035 | MTMR2 | -2.143 | 0.454 | 12 | 0.0005 | 0.0088 | 4.42 | Down |
| 52779 | NARS | -2.142 | 0.457 | 12 | 0.0005 | 0.0089 | 4.41 | Down |
| 5135 | RIOK2 | -2.142 | 0.395 | 12 | 0.0002 | 0.0053 | 4.41 | Down |
| 46604 | WDR75 | -2.142 | 0.456 | 12 | 0.0005 | 0.0089 | 4.41 | Down |
| 2566 | KIF1B | -2.142 | 0.328 | 12 | <.0001 | 0.0020 | 4.41 | Down |
| 51532 | EPHA4 | -2.141 | 0.336 | 12 | <.0001 | 0.0023 | 4.41 | Down |
| 15423 | SETD2 | -2.140 | 0.494 | 12 | 0.0010 | 0.0109 | 4.41 | Down |
| 48325 | RANBP17 | -2.140 | 0.478 | 12 | 0.0008 | 0.0100 | 4.41 | Down |
| 47562 | METTL14 | -2.135 | 0.438 | 12 | 0.0004 | 0.0080 | 4.39 | Down |
| 33459 | DDX10 | -2.134 | 0.439 | 12 | 0.0004 | 0.0081 | 4.39 | Down |
| 19272 | AK129671 | -2.134 | 0.296 | 12 | <.0001 | 0.0012 | 4.39 | Down |
| 55563 | MORC4 | -2.133 | 0.407 | 12 | 0.0002 | 0.0061 | 4.39 | Down |
| 30252 | NOC3L | -2.132 | 0.421 | 12 | 0.0003 | 0.0070 | 4.38 | Down |
| 46132 | DDX1 | -2.132 | 0.420 | 12 | 0.0003 | 0.0069 | 4.38 | Down |
| 30655 | EPHA4 | -2.131 | 0.377 | 12 | 0.0001 | 0.0044 | 4.38 | Down |
| 8350 | TOP2B | -2.130 | 0.459 | 12 | 0.0006 | 0.0092 | 4.38 | Down |
| 3430 | EPHA4 | -2.130 | 0.401 | 12 | 0.0002 | 0.0059 | 4.38 | Down |
| 21822 | GABPB1 | -2.126 | 0.424 | 12 | 0.0003 | 0.0072 | 4.37 | Down |
| 52758 | DNTTIP2 | -2.126 | 0.440 | 12 | 0.0004 | 0.0082 | 4.37 | Down |
| 50786 | RIOK2 | -2.126 | 0.434 | 12 | 0.0004 | 0.0079 | 4.37 | Down |
| 55444 | EPB41L2 | -2.123 | 0.480 | 12 | 0.0008 | 0.0103 | 4.36 | Down |
| 27689 | AFTPH | -2.120 | 0.459 | 12 | 0.0006 | 0.0093 | 4.35 | Down |
| 2105 | GPCPD1 | -2.120 | 0.472 | 12 | 0.0007 | 0.0100 | 4.35 | Down |
| 58134 | NOC3L | -2.119 | 0.457 | 12 | 0.0006 | 0.0092 | 4.34 | Down |
| 15425 | KIAA1432 | -2.118 | 0.384 | 12 | 0.0001 | 0.0050 | 4.34 | Down |
| 281 | KIAA0196 | -2.115 | 0.436 | 12 | 0.0004 | 0.0081 | 4.33 | Down |
| 9696 | ABCE1 | -2.112 | 0.485 | 12 | 0.0009 | 0.0108 | 4.32 | Down |
| 35060 | SSFA2 | -2.110 | 0.486 | 12 | 0.0010 | 0.0109 | 4.32 | Down |
| 23128 | DDX1 | -2.109 | 0.409 | 12 | 0.0002 | 0.0066 | 4.31 | Down |
| 53410 | ALG11 | -2.106 | 0.472 | 12 | 0.0008 | 0.0101 | 4.31 | Down |
| 45513 | LEO1 | -2.105 | 0.364 | 12 | <.0001 | 0.0039 | 4.30 | Down |
| 14227 | IPO7 | -2.104 | 0.417 | 12 | 0.0003 | 0.0071 | 4.30 | Down |
| 42188 | COPS4 | -2.103 | 0.460 | 12 | 0.0006 | 0.0095 | 4.29 | Down |
| 1406 | NOC3L | -2.100 | 0.476 | 12 | 0.0008 | 0.0104 | 4.29 | Down |
| 59642 | STAT1 | -2.100 | 0.455 | 12 | 0.0006 | 0.0093 | 4.29 | Down |
| 50716 | XRCC5 | -2.099 | 0.411 | 12 | 0.0003 | 0.0068 | 4.28 | Down |
| 31824 | KIAA0196 | -2.099 | 0.447 | 12 | 0.0005 | 0.0089 | 4.28 | Down |
| 56999 | ATAD2 | -2.099 | 0.455 | 12 | 0.0006 | 0.0093 | 4.28 | Down |
| 55882 | SNX2 | -2.098 | 0.457 | 12 | 0.0006 | 0.0094 | 4.28 | Down |
| 35971 | NARS | -2.098 | 0.464 | 12 | 0.0007 | 0.0098 | 4.28 | Down |
| 46452 | ITGB1 | -2.098 | 0.463 | 12 | 0.0007 | 0.0097 | 4.28 | Down |
| 20527 | PARP14 | -2.097 | 0.423 | 12 | 0.0003 | 0.0075 | 4.28 | Down |
| 55729 | THADA | -2.097 | 0.418 | 12 | 0.0003 | 0.0072 | 4.28 | Down |
| 39580 | RPS6KC1 | -2.097 | 0.475 | 12 | 0.0009 | 0.0104 | 4.28 | Down |
| 26369 | KIAA0196 | -2.096 | 0.474 | 12 | 0.0008 | 0.0103 | 4.28 | Down |
| 43711 | RIOK2 | -2.095 | 0.462 | 12 | 0.0007 | 0.0097 | 4.27 | Down |
| 7692 | DDX1 | -2.094 | 0.448 | 12 | 0.0005 | 0.0090 | 4.27 | Down |
| 3623 | DDX1 | -2.094 | 0.419 | 12 | 0.0003 | 0.0073 | 4.27 | Down |
| 55781 | TMEM22 | -2.092 | 0.477 | 12 | 0.0009 | 0.0106 | 4.26 | Down |
| 34415 | FRAS1 | -2.088 | 0.407 | 12 | 0.0002 | 0.0066 | 4.25 | Down |
| 11394 | ZNF37A | -2.087 | 0.459 | 12 | 0.0007 | 0.0097 | 4.25 | Down |
| 27087 | AKAP12 | -2.086 | 0.407 | 12 | 0.0003 | 0.0067 | 4.25 | Down |
| 32988 | RIOK2 | -2.085 | 0.450 | 12 | 0.0006 | 0.0093 | 4.24 | Down |
| 42184 | CDK5RAP2 | -2.085 | 0.398 | 12 | 0.0002 | 0.0062 | 4.24 | Down |
| 41853 | DNAJC2 | -2.084 | 0.439 | 12 | 0.0005 | 0.0086 | 4.24 | Down |
| 14130 | TMEM131 | -2.083 | 0.226 | 12 | <.0001 | 0.0003 | 4.24 | Down |
| 30956 | NBAS | -2.082 | 0.480 | 12 | 0.0010 | 0.0109 | 4.23 | Down |
| 32285 | TUG1 | -2.081 | 0.448 | 12 | 0.0006 | 0.0092 | 4.23 | Down |
| 7013 | EXOC6 | -2.081 | 0.464 | 12 | 0.0007 | 0.0100 | 4.23 | Down |
| 15510 | DDX1 | -2.080 | 0.405 | 12 | 0.0002 | 0.0066 | 4.23 | Down |
| 50337 | SRBD1 | -2.079 | 0.471 | 12 | 0.0009 | 0.0104 | 4.22 | Down |
| 45380 | TMEM2 | -2.077 | 0.375 | 12 | 0.0001 | 0.0049 | 4.22 | Down |
| 22573 | EPS15 | -2.077 | 0.436 | 12 | 0.0005 | 0.0085 | 4.22 | Down |
| 38078 | DDX1 | -2.076 | 0.414 | 12 | 0.0003 | 0.0072 | 4.22 | Down |
| 19142 | EPHA4 | -2.074 | 0.345 | 12 | <.0001 | 0.0032 | 4.21 | Down |
| 42059 | LTV1 | -2.073 | 0.456 | 12 | 0.0007 | 0.0097 | 4.21 | Down |
| 43676 | PHF20L1 | -2.072 | 0.425 | 12 | 0.0004 | 0.0080 | 4.20 | Down |
| 1244 | HSP90B3P | -2.071 | 0.461 | 12 | 0.0007 | 0.0100 | 4.20 | Down |
| 40751 | KIAA0196 | -2.070 | 0.465 | 12 | 0.0008 | 0.0102 | 4.20 | Down |
| 23469 | GFM2 | -2.068 | 0.447 | 12 | 0.0006 | 0.0093 | 4.19 | Down |
| 61875 | AFTPH | -2.067 | 0.467 | 12 | 0.0008 | 0.0103 | 4.19 | Down |
| 39161 | ABL2 | -2.067 | 0.438 | 12 | 0.0005 | 0.0088 | 4.19 | Down |
| 53468 | CRYBG3 | -2.066 | 0.414 | 12 | 0.0003 | 0.0073 | 4.19 | Down |
| 34449 | FAM169A | -2.064 | 0.418 | 12 | 0.0003 | 0.0076 | 4.18 | Down |
| 7763 | NARS | -2.061 | 0.460 | 12 | 0.0007 | 0.0100 | 4.17 | Down |
| 46847 | BMPR1A | -2.060 | 0.460 | 12 | 0.0007 | 0.0100 | 4.17 | Down |
| 45519 | MPHOSPH10 | -2.059 | 0.431 | 12 | 0.0005 | 0.0085 | 4.17 | Down |
| 22840 | BTN3A2 | -2.057 | 0.357 | 12 | <.0001 | 0.0040 | 4.16 | Down |
| 61341 | NTN4 | -2.057 | 0.398 | 12 | 0.0002 | 0.0065 | 4.16 | Down |
| 20853 | NARS | -2.056 | 0.433 | 12 | 0.0005 | 0.0086 | 4.16 | Down |
| 29059 | RECQL | -2.056 | 0.391 | 12 | 0.0002 | 0.0060 | 4.16 | Down |
| 37354 | SPP1 | -2.055 | 0.402 | 12 | 0.0003 | 0.0067 | 4.16 | Down |
| 48308 | ELMOD2 | -2.055 | 0.448 | 12 | 0.0006 | 0.0094 | 4.15 | Down |
| 58587 | DDX1 | -2.055 | 0.418 | 12 | 0.0004 | 0.0077 | 4.15 | Down |
| 11353 | VEPH1 | -2.054 | 0.464 | 12 | 0.0008 | 0.0103 | 4.15 | Down |
| 39195 | NARS | -2.054 | 0.467 | 12 | 0.0009 | 0.0105 | 4.15 | Down |
| 2156 | AFTPH | -2.052 | 0.408 | 12 | 0.0003 | 0.0071 | 4.15 | Down |
| 20375 | MKI67 | -2.050 | 0.323 | 12 | <.0001 | 0.0023 | 4.14 | Down |
| 7465 | RGPD5 | -2.050 | 0.431 | 12 | 0.0005 | 0.0086 | 4.14 | Down |
| 5163 | SLMAP | -2.048 | 0.448 | 12 | 0.0006 | 0.0095 | 4.14 | Down |
| 49367 | NEK2 | -2.048 | 0.260 | 12 | <.0001 | 0.0007 | 4.14 | Down |
| 38721 | NARS | -2.047 | 0.423 | 12 | 0.0004 | 0.0082 | 4.13 | Down |
| 18861 | IDE | -2.046 | 0.416 | 12 | 0.0004 | 0.0077 | 4.13 | Down |
| 36385 | DTL | -2.045 | 0.365 | 12 | 0.0001 | 0.0047 | 4.13 | Down |
| 28052 | OGT | -2.043 | 0.463 | 12 | 0.0008 | 0.0104 | 4.12 | Down |
| 29341 | TP53BP1 | -2.043 | 0.412 | 12 | 0.0003 | 0.0075 | 4.12 | Down |
| 58154 | DDX1 | -2.041 | 0.400 | 12 | 0.0003 | 0.0068 | 4.12 | Down |
| 22151 | EML4 | -2.041 | 0.412 | 12 | 0.0003 | 0.0075 | 4.11 | Down |
| 24976 | TCF12 | -2.041 | 0.369 | 12 | 0.0001 | 0.0050 | 4.11 | Down |
| 27979 | TIGD2 | -2.040 | 0.456 | 12 | 0.0008 | 0.0100 | 4.11 | Down |
| 24942 | IGBP1 | -2.039 | 0.456 | 12 | 0.0008 | 0.0101 | 4.11 | Down |
| 34498 | LTV1 | -2.039 | 0.456 | 12 | 0.0008 | 0.0100 | 4.11 | Down |
| 17623 | THADA | -2.039 | 0.393 | 12 | 0.0002 | 0.0064 | 4.11 | Down |
| 289 | THADA | -2.038 | 0.352 | 12 | <.0001 | 0.0039 | 4.11 | Down |
| 16374 | LARP7 | -2.038 | 0.450 | 12 | 0.0007 | 0.0098 | 4.11 | Down |
| 33557 | RIOK2 | -2.036 | 0.432 | 12 | 0.0005 | 0.0089 | 4.10 | Down |
| 10311 | PDZD2 | -2.036 | 0.440 | 12 | 0.0006 | 0.0093 | 4.10 | Down |
| 46203 | DIP2B | -2.036 | 0.451 | 12 | 0.0007 | 0.0098 | 4.10 | Down |
| 42875 | PLK1S1 | -2.035 | 0.438 | 12 | 0.0006 | 0.0092 | 4.10 | Down |
| 41940 | DDX1 | -2.032 | 0.414 | 12 | 0.0004 | 0.0077 | 4.09 | Down |
| 58493 | ITGB1 | -2.030 | 0.429 | 12 | 0.0005 | 0.0087 | 4.08 | Down |
| 43915 | CARD6 | -2.028 | 0.447 | 12 | 0.0007 | 0.0097 | 4.08 | Down |
| 45081 | DDX18 | -2.027 | 0.429 | 12 | 0.0005 | 0.0088 | 4.07 | Down |
| 52217 | VRK2 | -2.027 | 0.453 | 12 | 0.0008 | 0.0100 | 4.07 | Down |
| 23078 | IPO5 | -2.026 | 0.358 | 12 | 0.0001 | 0.0044 | 4.07 | Down |
| 7610 | AFTPH | -2.025 | 0.422 | 12 | 0.0004 | 0.0083 | 4.07 | Down |
| 5969 | ANXA1 | -2.024 | 0.347 | 12 | <.0001 | 0.0038 | 4.07 | Down |
| 37981 | BCLAF1 | -2.021 | 0.377 | 12 | 0.0002 | 0.0056 | 4.06 | Down |
| 19989 | TANC1 | -2.021 | 0.380 | 12 | 0.0002 | 0.0058 | 4.06 | Down |
| 12653 | GPBP1 | -2.020 | 0.444 | 12 | 0.0007 | 0.0097 | 4.06 | Down |
| 14112 | LTV1 | -2.020 | 0.424 | 12 | 0.0005 | 0.0085 | 4.06 | Down |
| 24639 | LTV1 | -2.016 | 0.425 | 12 | 0.0005 | 0.0086 | 4.04 | Down |
| 28964 | KIAA0196 | -2.016 | 0.413 | 12 | 0.0004 | 0.0080 | 4.04 | Down |
| 9128 | NCBP2 | -2.013 | 0.311 | 12 | <.0001 | 0.0021 | 4.04 | Down |
| 29422 | DSP | -2.011 | 0.315 | 12 | <.0001 | 0.0023 | 4.03 | Down |
| 20751 | TAX1BP1 | -2.011 | 0.374 | 12 | 0.0002 | 0.0055 | 4.03 | Down |
| 28524 | ZC3H11A | -2.011 | 0.461 | 12 | 0.0009 | 0.0107 | 4.03 | Down |
| 15278 | NUP107 | -2.010 | 0.418 | 12 | 0.0004 | 0.0083 | 4.03 | Down |
| 47355 | C2orf3 | -2.010 | 0.429 | 12 | 0.0005 | 0.0089 | 4.03 | Down |
| 50559 | MSH3 | -2.009 | 0.321 | 12 | <.0001 | 0.0025 | 4.03 | Down |
| 44698 | PRKAB2 | -2.007 | 0.453 | 12 | 0.0008 | 0.0103 | 4.02 | Down |
| 47905 | GDAP2 | -2.007 | 0.422 | 12 | 0.0005 | 0.0085 | 4.02 | Down |
| 559 | TRIM59 | -2.006 | 0.347 | 12 | <.0001 | 0.0039 | 4.02 | Down |
| 53508 | MIA3 | -2.004 | 0.397 | 12 | 0.0003 | 0.0070 | 4.01 | Down |
| 37968 | ALKBH8 | -2.004 | 0.308 | 12 | <.0001 | 0.0021 | 4.01 | Down |
| 57557 | NARS | -2.002 | 0.445 | 12 | 0.0007 | 0.0099 | 4.00 | Down |
| 32204 | LTV1 | -2.001 | 0.452 | 12 | 0.0008 | 0.0103 | 4.00 | Down |
| 10588 | SUV420H1 | -1.998 | 0.408 | 12 | 0.0004 | 0.0078 | 3.99 | Down |
| 46806 | SEPT7 | -1.997 | 0.454 | 12 | 0.0009 | 0.0105 | 3.99 | Down |
| 43729 | RLIM | -1.996 | 0.434 | 12 | 0.0006 | 0.0094 | 3.99 | Down |
| 25543 | TBC1D15 | -1.996 | 0.379 | 12 | 0.0002 | 0.0060 | 3.99 | Down |
| 46189 | ZNF322 | -1.994 | 0.433 | 12 | 0.0006 | 0.0094 | 3.98 | Down |
| 11709 | PTPN11 | -1.994 | 0.384 | 12 | 0.0002 | 0.0064 | 3.98 | Down |
| 25394 | NUCB2 | -1.993 | 0.404 | 12 | 0.0003 | 0.0076 | 3.98 | Down |
| 41636 | SETBP1 | -1.989 | 0.428 | 12 | 0.0006 | 0.0092 | 3.97 | Down |
| 54189 | DTL | -1.988 | 0.377 | 12 | 0.0002 | 0.0060 | 3.97 | Down |
| 54654 | AFTPH | -1.986 | 0.449 | 12 | 0.0008 | 0.0104 | 3.96 | Down |
| 22261 | CSE1L | -1.986 | 0.408 | 12 | 0.0004 | 0.0080 | 3.96 | Down |
| 17265 | THADA | -1.985 | 0.447 | 12 | 0.0008 | 0.0103 | 3.96 | Down |
| 11971 | TMCC1 | -1.985 | 0.456 | 12 | 0.0009 | 0.0108 | 3.96 | Down |
| 38979 | TOP2B | -1.982 | 0.451 | 12 | 0.0009 | 0.0105 | 3.95 | Down |
| 60834 | MLL3 | -1.981 | 0.374 | 12 | 0.0002 | 0.0059 | 3.95 | Down |
| 11176 | COPB2 | -1.979 | 0.386 | 12 | 0.0002 | 0.0066 | 3.94 | Down |
| 44626 | COPB1 | -1.978 | 0.455 | 12 | 0.0009 | 0.0108 | 3.94 | Down |
| 24698 | ITGB1 | -1.978 | 0.433 | 12 | 0.0006 | 0.0095 | 3.94 | Down |
| 29778 | DTL | -1.977 | 0.347 | 12 | <.0001 | 0.0043 | 3.94 | Down |
| 27659 | ZNFX1 | -1.976 | 0.371 | 12 | 0.0002 | 0.0058 | 3.94 | Down |
| 31771 | CAMSAP2 | -1.976 | 0.443 | 12 | 0.0008 | 0.0101 | 3.93 | Down |
| 20890 | EEF1A1 | -1.973 | 0.384 | 12 | 0.0002 | 0.0066 | 3.93 | Down |
| 3692 | ANKRD36BP2 | -1.972 | 0.445 | 12 | 0.0008 | 0.0103 | 3.92 | Down |
| 37205 | AKAP12 | -1.966 | 0.353 | 12 | 0.0001 | 0.0048 | 3.91 | Down |
| 11677 | ZFYVE26 | -1.965 | 0.379 | 12 | 0.0002 | 0.0065 | 3.90 | Down |
| 51252 | XLOC_l2_0082 | -1.964 | 0.363 | 12 | 0.0002 | 0.0053 | 3.90 | Down |
| 29156 | LTV1 | -1.963 | 0.441 | 12 | 0.0008 | 0.0101 | 3.90 | Down |
| 25776 | NARS | -1.962 | 0.423 | 12 | 0.0006 | 0.0092 | 3.90 | Down |
| 1486 | ATG16L1 | -1.960 | 0.225 | 12 | <.0001 | 0.0004 | 3.89 | Down |
| 40505 | GGNBP2 | -1.958 | 0.437 | 12 | 0.0008 | 0.0100 | 3.88 | Down |
| 198 | ZNF195 | -1.957 | 0.284 | 12 | <.0001 | 0.0015 | 3.88 | Down |
| 49203 | AKR1B15 | -1.955 | 0.423 | 12 | 0.0006 | 0.0093 | 3.88 | Down |
| 13103 | KIF1B | -1.955 | 0.431 | 12 | 0.0007 | 0.0097 | 3.88 | Down |
| 49797 | EPHA4 | -1.953 | 0.356 | 12 | 0.0001 | 0.0051 | 3.87 | Down |
| 24367 | NARS | -1.952 | 0.422 | 12 | 0.0006 | 0.0093 | 3.87 | Down |
| 18526 | HACL1 | -1.952 | 0.308 | 12 | <.0001 | 0.0024 | 3.87 | Down |
| 119 | MSH3 | -1.951 | 0.370 | 12 | 0.0002 | 0.0060 | 3.87 | Down |
| 5345 | APC | -1.944 | 0.434 | 12 | 0.0008 | 0.0100 | 3.85 | Down |
| 4349 | NEDD1 | -1.944 | 0.446 | 12 | 0.0009 | 0.0107 | 3.85 | Down |
| 13715 | RAB3GAP1 | -1.940 | 0.418 | 12 | 0.0006 | 0.0092 | 3.84 | Down |
| 16808 | AVL9 | -1.940 | 0.426 | 12 | 0.0007 | 0.0097 | 3.84 | Down |
| 27048 | BUB1 | -1.930 | 0.417 | 12 | 0.0006 | 0.0092 | 3.81 | Down |
| 35652 | ANKHD1-EIF4E | -1.930 | 0.360 | 12 | 0.0002 | 0.0056 | 3.81 | Down |
| 43289 | RAB3GAP1 | -1.929 | 0.426 | 12 | 0.0007 | 0.0098 | 3.81 | Down |
| 52024 | THADA | -1.929 | 0.363 | 12 | 0.0002 | 0.0058 | 3.81 | Down |
| 14393 | ZFP106 | -1.928 | 0.357 | 12 | 0.0002 | 0.0054 | 3.80 | Down |
| 18037 | MSH3 | -1.925 | 0.397 | 12 | 0.0004 | 0.0081 | 3.80 | Down |
| 52320 | ANKRD36 | -1.923 | 0.387 | 12 | 0.0003 | 0.0074 | 3.79 | Down |
| 15454 | THADA | -1.922 | 0.362 | 12 | 0.0002 | 0.0059 | 3.79 | Down |
| 11653 | DTL | -1.921 | 0.363 | 12 | 0.0002 | 0.0059 | 3.79 | Down |
| 26048 | CCNB1 | -1.919 | 0.298 | 12 | <.0001 | 0.0021 | 3.78 | Down |
| 9740 | PTPN11 | -1.918 | 0.425 | 12 | 0.0007 | 0.0098 | 3.78 | Down |
| 37849 | HACL1 | -1.916 | 0.360 | 12 | 0.0002 | 0.0058 | 3.78 | Down |
| 50188 | MSH6 | -1.914 | 0.376 | 12 | 0.0003 | 0.0068 | 3.77 | Down |
| 21638 | ETFDH | -1.913 | 0.358 | 12 | 0.0002 | 0.0057 | 3.77 | Down |
| 33792 | CASP5 | -1.911 | 0.311 | 12 | <.0001 | 0.0028 | 3.76 | Down |
| 53868 | A_33_P332533 | -1.906 | 0.368 | 12 | 0.0002 | 0.0065 | 3.75 | Down |
| 16781 | TDRD7 | -1.905 | 0.438 | 12 | 0.0010 | 0.0108 | 3.75 | Down |
| 23407 | RARS | -1.904 | 0.353 | 12 | 0.0002 | 0.0054 | 3.74 | Down |
| 43774 | N4BP2 | -1.904 | 0.396 | 12 | 0.0004 | 0.0083 | 3.74 | Down |
| 10714 | EEF1A1 | -1.899 | 0.375 | 12 | 0.0003 | 0.0069 | 3.73 | Down |
| 59849 | TTC12 | -1.896 | 0.322 | 12 | <.0001 | 0.0036 | 3.72 | Down |
| 17307 | MAGI3 | -1.894 | 0.430 | 12 | 0.0009 | 0.0104 | 3.72 | Down |
| 1291 | LAMB1 | -1.893 | 0.339 | 12 | 0.0001 | 0.0048 | 3.71 | Down |
| 14205 | FAM115A | -1.892 | 0.433 | 12 | 0.0009 | 0.0107 | 3.71 | Down |
| 6303 | HACL1 | -1.890 | 0.344 | 12 | 0.0001 | 0.0051 | 3.71 | Down |
| 45079 | THADA | -1.888 | 0.417 | 12 | 0.0007 | 0.0098 | 3.70 | Down |
| 14442 | RBM39 | -1.886 | 0.415 | 12 | 0.0007 | 0.0097 | 3.70 | Down |
| 4155 | MPP6 | -1.884 | 0.344 | 12 | 0.0001 | 0.0052 | 3.69 | Down |
| 50456 | LIMCH1 | -1.884 | 0.397 | 12 | 0.0005 | 0.0086 | 3.69 | Down |
| 40338 | RBM39 | -1.882 | 0.413 | 12 | 0.0007 | 0.0097 | 3.69 | Down |
| 41351 | DTL | -1.880 | 0.386 | 12 | 0.0004 | 0.0080 | 3.68 | Down |
| 46979 | CCNB1 | -1.880 | 0.319 | 12 | <.0001 | 0.0035 | 3.68 | Down |
| 62613 | BAZ1B | -1.875 | 0.333 | 12 | 0.0001 | 0.0046 | 3.67 | Down |
| 32771 | DTL | -1.875 | 0.364 | 12 | 0.0002 | 0.0066 | 3.67 | Down |
| 43349 | KIAA0196 | -1.875 | 0.406 | 12 | 0.0006 | 0.0093 | 3.67 | Down |
| 180 | RABGAP1 | -1.874 | 0.422 | 12 | 0.0008 | 0.0102 | 3.67 | Down |
| 2327 | NUP205 | -1.874 | 0.379 | 12 | 0.0003 | 0.0075 | 3.66 | Down |
| 15286 | EPS8 | -1.871 | 0.405 | 12 | 0.0006 | 0.0093 | 3.66 | Down |
| 48878 | RMI1 | -1.870 | 0.224 | 12 | <.0001 | 0.0005 | 3.66 | Down |
| 8662 | RAI14 | -1.869 | 0.376 | 12 | 0.0003 | 0.0074 | 3.65 | Down |
| 20536 | ZNF608 | -1.869 | 0.394 | 12 | 0.0005 | 0.0087 | 3.65 | Down |
| 10371 | SEC24D | -1.868 | 0.422 | 12 | 0.0008 | 0.0103 | 3.65 | Down |
| 54092 | CCNB1 | -1.866 | 0.293 | 12 | <.0001 | 0.0023 | 3.65 | Down |
| 44354 | RRM1 | -1.865 | 0.372 | 12 | 0.0003 | 0.0072 | 3.64 | Down |
| 54927 | CCNB1 | -1.863 | 0.313 | 12 | <.0001 | 0.0034 | 3.64 | Down |
| 3274 | CASP4 | -1.863 | 0.270 | 12 | <.0001 | 0.0015 | 3.64 | Down |
| 14774 | HACL1 | -1.862 | 0.329 | 12 | 0.0001 | 0.0044 | 3.64 | Down |
| 19653 | RPAP2 | -1.862 | 0.422 | 12 | 0.0008 | 0.0104 | 3.64 | Down |
| 59017 | CCT2 | -1.862 | 0.363 | 12 | 0.0002 | 0.0066 | 3.64 | Down |
| 10719 | IFIH1 | -1.862 | 0.419 | 12 | 0.0008 | 0.0102 | 3.63 | Down |
| 47363 | EIF3A | -1.861 | 0.384 | 12 | 0.0004 | 0.0081 | 3.63 | Down |
| 28848 | HACL1 | -1.859 | 0.345 | 12 | 0.0002 | 0.0055 | 3.63 | Down |
| 31707 | ASXL2 | -1.859 | 0.343 | 12 | 0.0002 | 0.0053 | 3.63 | Down |
| 54224 | DYNC1H1 | -1.858 | 0.228 | 12 | <.0001 | 0.0006 | 3.63 | Down |
| 51475 | CCT2 | -1.858 | 0.375 | 12 | 0.0003 | 0.0075 | 3.63 | Down |
| 44298 | KIAA1797 | -1.858 | 0.373 | 12 | 0.0003 | 0.0074 | 3.62 | Down |
| 11791 | ZMYM4 | -1.857 | 0.404 | 12 | 0.0006 | 0.0094 | 3.62 | Down |
| 56768 | THADA | -1.856 | 0.370 | 12 | 0.0003 | 0.0072 | 3.62 | Down |
| 2140 | BMP4 | -1.855 | 0.320 | 12 | <.0001 | 0.0039 | 3.62 | Down |
| 11741 | DTL | -1.854 | 0.325 | 12 | <.0001 | 0.0043 | 3.62 | Down |
| 61282 | MAP3K1 | -1.853 | 0.419 | 12 | 0.0008 | 0.0103 | 3.61 | Down |
| 7387 | FANCI | -1.852 | 0.394 | 12 | 0.0005 | 0.0089 | 3.61 | Down |
| 18446 | SYNE2 | -1.849 | 0.382 | 12 | 0.0004 | 0.0081 | 3.60 | Down |
| 40700 | ZNF195 | -1.846 | 0.424 | 12 | 0.0009 | 0.0108 | 3.59 | Down |
| 1716 | TDRD7 | -1.846 | 0.422 | 12 | 0.0009 | 0.0106 | 3.59 | Down |
| 55232 | MYOF | -1.846 | 0.313 | 12 | <.0001 | 0.0036 | 3.59 | Down |
| 25234 | MTMR10 | -1.845 | 0.296 | 12 | <.0001 | 0.0026 | 3.59 | Down |
| 12198 | PTPN11 | -1.844 | 0.411 | 12 | 0.0007 | 0.0100 | 3.59 | Down |
| 19893 | MSH3 | -1.839 | 0.411 | 12 | 0.0008 | 0.0100 | 3.58 | Down |
| 62119 | MSH3 | -1.839 | 0.395 | 12 | 0.0006 | 0.0092 | 3.58 | Down |
| 62880 | CCNE2 | -1.839 | 0.419 | 12 | 0.0009 | 0.0106 | 3.58 | Down |
| 26047 | HMGCS1 | -1.838 | 0.408 | 12 | 0.0007 | 0.0099 | 3.58 | Down |
| 3851 | CCDC75 | -1.838 | 0.369 | 12 | 0.0003 | 0.0074 | 3.58 | Down |
| 17364 | MGA | -1.835 | 0.423 | 12 | 0.0010 | 0.0109 | 3.57 | Down |
| 17026 | C2orf3 | -1.835 | 0.413 | 12 | 0.0008 | 0.0103 | 3.57 | Down |
| 9749 | RBM39 | -1.835 | 0.421 | 12 | 0.0009 | 0.0108 | 3.57 | Down |
| 8580 | CCNB1 | -1.832 | 0.295 | 12 | <.0001 | 0.0027 | 3.56 | Down |
| 54867 | DTL | -1.829 | 0.328 | 12 | 0.0001 | 0.0048 | 3.55 | Down |
| 44106 | TOR1AIP2 | -1.828 | 0.403 | 12 | 0.0007 | 0.0097 | 3.55 | Down |
| 35413 | CCNB1 | -1.827 | 0.302 | 12 | <.0001 | 0.0031 | 3.55 | Down |
| 6389 | HACL1 | -1.825 | 0.356 | 12 | 0.0003 | 0.0067 | 3.54 | Down |
| 49392 | MSH3 | -1.824 | 0.398 | 12 | 0.0006 | 0.0094 | 3.54 | Down |
| 52688 | HACL1 | -1.824 | 0.309 | 12 | <.0001 | 0.0036 | 3.54 | Down |
| 13465 | PLAGL1 | -1.823 | 0.324 | 12 | 0.0001 | 0.0046 | 3.54 | Down |
| 3987 | PRPF18 | -1.823 | 0.396 | 12 | 0.0006 | 0.0094 | 3.54 | Down |
| 23433 | THADA | -1.822 | 0.408 | 12 | 0.0008 | 0.0101 | 3.53 | Down |
| 54811 | CCT2 | -1.820 | 0.371 | 12 | 0.0004 | 0.0078 | 3.53 | Down |
| 9059 | PHACTR2 | -1.816 | 0.301 | 12 | <.0001 | 0.0031 | 3.52 | Down |
| 32363 | DSP | -1.816 | 0.224 | 12 | <.0001 | 0.0006 | 3.52 | Down |
| 47273 | TIPARP | -1.815 | 0.379 | 12 | 0.0004 | 0.0084 | 3.52 | Down |
| 28683 | CCNB1 | -1.813 | 0.288 | 12 | <.0001 | 0.0024 | 3.51 | Down |
| 3880 | HACL1 | -1.810 | 0.326 | 12 | 0.0001 | 0.0049 | 3.51 | Down |
| 10133 | LIMS3-LOC440 | -1.808 | 0.221 | 12 | <.0001 | 0.0005 | 3.50 | Down |
| 1605 | ZNHIT6 | -1.808 | 0.352 | 12 | 0.0002 | 0.0066 | 3.50 | Down |
| 1386 | MKL2 | -1.805 | 0.387 | 12 | 0.0005 | 0.0091 | 3.49 | Down |
| 54368 | MYO6 | -1.801 | 0.344 | 12 | 0.0002 | 0.0062 | 3.49 | Down |
| 58159 | TNIK | -1.801 | 0.389 | 12 | 0.0006 | 0.0093 | 3.49 | Down |
| 50009 | DTL | -1.801 | 0.385 | 12 | 0.0005 | 0.0090 | 3.48 | Down |
| 34585 | CCNB1 | -1.800 | 0.309 | 12 | <.0001 | 0.0038 | 3.48 | Down |
| 23066 | CCT2 | -1.798 | 0.370 | 12 | 0.0004 | 0.0081 | 3.48 | Down |
| 44929 | CCNB1 | -1.797 | 0.291 | 12 | <.0001 | 0.0027 | 3.48 | Down |
| 15251 | EIF2A | -1.797 | 0.399 | 12 | 0.0007 | 0.0099 | 3.48 | Down |
| 41979 | CCNB1 | -1.797 | 0.313 | 12 | <.0001 | 0.0041 | 3.47 | Down |
| 51350 | HACL1 | -1.794 | 0.345 | 12 | 0.0002 | 0.0064 | 3.47 | Down |
| 18994 | SLC26A2 | -1.794 | 0.414 | 12 | 0.0010 | 0.0109 | 3.47 | Down |
| 13923 | VPS35 | -1.788 | 0.328 | 12 | 0.0001 | 0.0052 | 3.45 | Down |
| 45048 | LGALS8 | -1.788 | 0.391 | 12 | 0.0006 | 0.0095 | 3.45 | Down |
| 17632 | ZNF195 | -1.787 | 0.408 | 12 | 0.0009 | 0.0106 | 3.45 | Down |
| 1800 | THC2537477 | -1.785 | 0.407 | 12 | 0.0009 | 0.0106 | 3.45 | Down |
| 45636 | CCT2 | -1.783 | 0.369 | 12 | 0.0004 | 0.0082 | 3.44 | Down |
| 62604 | SRP54 | -1.783 | 0.413 | 12 | 0.0010 | 0.0111 | 3.44 | Down |
| 36332 | IL1B | -1.781 | 0.181 | 12 | <.0001 | 0.0002 | 3.44 | Down |
| 39521 | ACADM | -1.781 | 0.403 | 12 | 0.0008 | 0.0104 | 3.44 | Down |
| 368 | CCT2 | -1.780 | 0.370 | 12 | 0.0004 | 0.0083 | 3.44 | Down |
| 30720 | USP48 | -1.780 | 0.389 | 12 | 0.0006 | 0.0095 | 3.44 | Down |
| 54775 | KATNA1 | -1.780 | 0.384 | 12 | 0.0006 | 0.0092 | 3.43 | Down |
| 48275 | DPY19L4 | -1.773 | 0.403 | 12 | 0.0009 | 0.0105 | 3.42 | Down |
| 50083 | XLOC_010720 | -1.773 | 0.390 | 12 | 0.0007 | 0.0097 | 3.42 | Down |
| 30585 | MALAT1 | -1.772 | 0.384 | 12 | 0.0006 | 0.0093 | 3.42 | Down |
| 40172 | AHNAK | -1.768 | 0.277 | 12 | <.0001 | 0.0023 | 3.41 | Down |
| 17377 | CDC40 | -1.766 | 0.401 | 12 | 0.0009 | 0.0104 | 3.40 | Down |
| 3778 | LCLAT1 | -1.765 | 0.239 | 12 | <.0001 | 0.0010 | 3.40 | Down |
| 40531 | C22orf43 | -1.764 | 0.370 | 12 | 0.0005 | 0.0085 | 3.40 | Down |
| 57106 | COPG2 | -1.762 | 0.394 | 12 | 0.0008 | 0.0100 | 3.39 | Down |
| 51276 | SLU7 | -1.759 | 0.407 | 12 | 0.0010 | 0.0110 | 3.38 | Down |
| 14685 | ZADH2 | -1.758 | 0.335 | 12 | 0.0002 | 0.0061 | 3.38 | Down |
| 62006 | MSH6 | -1.753 | 0.385 | 12 | 0.0007 | 0.0097 | 3.37 | Down |
| 13151 | ZNF562 | -1.752 | 0.374 | 12 | 0.0005 | 0.0089 | 3.37 | Down |
| 21037 | MRE11A | -1.752 | 0.362 | 12 | 0.0004 | 0.0081 | 3.37 | Down |
| 5319 | SPTAN1 | -1.751 | 0.290 | 12 | <.0001 | 0.0031 | 3.36 | Down |
| 56215 | SEPT7P2 | -1.750 | 0.388 | 12 | 0.0007 | 0.0099 | 3.36 | Down |
| 45227 | DTL | -1.750 | 0.384 | 12 | 0.0007 | 0.0097 | 3.36 | Down |
| 33141 | SEPT7 | -1.739 | 0.376 | 12 | 0.0006 | 0.0093 | 3.34 | Down |
| 941 | ABL2 | -1.734 | 0.329 | 12 | 0.0002 | 0.0060 | 3.33 | Down |
| 57762 | FAM149B1 | -1.734 | 0.385 | 12 | 0.0007 | 0.0099 | 3.33 | Down |
| 22698 | PHACTR2 | -1.734 | 0.341 | 12 | 0.0003 | 0.0069 | 3.33 | Down |
| 32000 | ANAPC4 | -1.730 | 0.396 | 12 | 0.0009 | 0.0107 | 3.32 | Down |
| 10770 | GNL2 | -1.728 | 0.388 | 12 | 0.0008 | 0.0101 | 3.31 | Down |
| 43280 | PRDM10 | -1.727 | 0.378 | 12 | 0.0006 | 0.0096 | 3.31 | Down |
| 8169 | AFTPH | -1.725 | 0.391 | 12 | 0.0009 | 0.0104 | 3.31 | Down |
| 28938 | ELL2 | -1.724 | 0.308 | 12 | 0.0001 | 0.0047 | 3.30 | Down |
| 10953 | PLRG1 | -1.721 | 0.346 | 12 | 0.0003 | 0.0074 | 3.30 | Down |
| 11378 | CCT2 | -1.715 | 0.353 | 12 | 0.0004 | 0.0081 | 3.28 | Down |
| 42618 | SMC1A | -1.709 | 0.298 | 12 | <.0001 | 0.0041 | 3.27 | Down |
| 21484 | RBM26 | -1.708 | 0.394 | 12 | 0.0010 | 0.0109 | 3.27 | Down |
| 49932 | MKLN1 | -1.707 | 0.376 | 12 | 0.0007 | 0.0097 | 3.27 | Down |
| 40298 | HACL1 | -1.707 | 0.341 | 12 | 0.0003 | 0.0073 | 3.26 | Down |
| 12220 | HSPH1 | -1.706 | 0.385 | 12 | 0.0008 | 0.0103 | 3.26 | Down |
| 23199 | MSH6 | -1.706 | 0.385 | 12 | 0.0008 | 0.0103 | 3.26 | Down |
| 37891 | MSH6 | -1.706 | 0.357 | 12 | 0.0005 | 0.0085 | 3.26 | Down |
| 44099 | GIGYF2 | -1.705 | 0.336 | 12 | 0.0003 | 0.0069 | 3.26 | Down |
| 45906 | PHF6 | -1.702 | 0.380 | 12 | 0.0008 | 0.0100 | 3.25 | Down |
| 38409 | PHACTR2 | -1.701 | 0.356 | 12 | 0.0005 | 0.0085 | 3.25 | Down |
| 8878 | RBM27 | -1.701 | 0.387 | 12 | 0.0009 | 0.0105 | 3.25 | Down |
| 11680 | TARS | -1.701 | 0.368 | 12 | 0.0006 | 0.0093 | 3.25 | Down |
| 48277 | CCT2 | -1.698 | 0.366 | 12 | 0.0006 | 0.0092 | 3.24 | Down |
| 19376 | MSH6 | -1.696 | 0.383 | 12 | 0.0008 | 0.0103 | 3.24 | Down |
| 49509 | KIAA0586 | -1.695 | 0.342 | 12 | 0.0003 | 0.0075 | 3.24 | Down |
| 37976 | MSH6 | -1.693 | 0.392 | 12 | 0.0010 | 0.0111 | 3.23 | Down |
| 34410 | FBXW7 | -1.693 | 0.318 | 12 | 0.0002 | 0.0058 | 3.23 | Down |
| 33427 | YME1L1 | -1.691 | 0.378 | 12 | 0.0008 | 0.0100 | 3.23 | Down |
| 51506 | ACACA | -1.690 | 0.311 | 12 | 0.0002 | 0.0053 | 3.23 | Down |
| 59646 | ZNF252 | -1.689 | 0.388 | 12 | 0.0009 | 0.0108 | 3.22 | Down |
| 60602 | AIMP1 | -1.689 | 0.384 | 12 | 0.0009 | 0.0105 | 3.22 | Down |
| 26320 | ANAPC1 | -1.687 | 0.378 | 12 | 0.0008 | 0.0101 | 3.22 | Down |
| 25793 | KIAA0586 | -1.686 | 0.360 | 12 | 0.0005 | 0.0089 | 3.22 | Down |
| 62884 | EHBP1 | -1.686 | 0.365 | 12 | 0.0006 | 0.0093 | 3.22 | Down |
| 15367 | CCT2 | -1.685 | 0.373 | 12 | 0.0007 | 0.0098 | 3.22 | Down |
| 8871 | TUG1 | -1.684 | 0.358 | 12 | 0.0005 | 0.0089 | 3.21 | Down |
| 4704 | DNAJA1P5 | -1.684 | 0.371 | 12 | 0.0007 | 0.0097 | 3.21 | Down |
| 18321 | MAN1A2 | -1.684 | 0.389 | 12 | 0.0010 | 0.0110 | 3.21 | Down |
| 7189 | FAM46A | -1.681 | 0.372 | 12 | 0.0007 | 0.0098 | 3.21 | Down |
| 33810 | PSMD1 | -1.677 | 0.351 | 12 | 0.0004 | 0.0084 | 3.20 | Down |
| 61646 | PLEKHA8 | -1.675 | 0.380 | 12 | 0.0009 | 0.0104 | 3.19 | Down |
| 35574 | MSH6 | -1.674 | 0.371 | 12 | 0.0007 | 0.0098 | 3.19 | Down |
| 27954 | ZMYND11 | -1.674 | 0.194 | 12 | <.0001 | 0.0004 | 3.19 | Down |
| 16137 | PYCR1 | -1.673 | 0.246 | 12 | <.0001 | 0.0016 | 3.19 | Down |
| 30718 | KIAA0586 | -1.670 | 0.321 | 12 | 0.0002 | 0.0064 | 3.18 | Down |
| 60036 | AKAP13 | -1.668 | 0.331 | 12 | 0.0003 | 0.0071 | 3.18 | Down |
| 21853 | MACF1 | -1.666 | 0.322 | 12 | 0.0002 | 0.0065 | 3.17 | Down |
| 55901 | USP41 | -1.666 | 0.326 | 12 | 0.0003 | 0.0068 | 3.17 | Down |
| 61860 | AIMP1 | -1.665 | 0.384 | 12 | 0.0010 | 0.0109 | 3.17 | Down |
| 3330 | MSH6 | -1.665 | 0.371 | 12 | 0.0007 | 0.0100 | 3.17 | Down |
| 276 | KATNA1 | -1.662 | 0.369 | 12 | 0.0007 | 0.0099 | 3.17 | Down |
| 61416 | BNIP3L | -1.661 | 0.380 | 12 | 0.0009 | 0.0107 | 3.16 | Down |
| 12671 | PPP2R3C | -1.657 | 0.374 | 12 | 0.0008 | 0.0103 | 3.15 | Down |
| 57350 | KIAA0586 | -1.655 | 0.360 | 12 | 0.0006 | 0.0094 | 3.15 | Down |
| 12453 | CDC27 | -1.655 | 0.375 | 12 | 0.0008 | 0.0104 | 3.15 | Down |
| 36007 | PHACTR2 | -1.652 | 0.382 | 12 | 0.0010 | 0.0110 | 3.14 | Down |
| 39029 | RGL1 | -1.651 | 0.346 | 12 | 0.0005 | 0.0085 | 3.14 | Down |
| 20836 | PLEKHA1 | -1.651 | 0.345 | 12 | 0.0004 | 0.0085 | 3.14 | Down |
| 40010 | PLOD2 | -1.645 | 0.341 | 12 | 0.0004 | 0.0082 | 3.13 | Down |
| 46578 | VPS36 | -1.644 | 0.371 | 12 | 0.0008 | 0.0103 | 3.13 | Down |
| 56556 | PAK1IP1 | -1.643 | 0.363 | 12 | 0.0007 | 0.0098 | 3.12 | Down |
| 10444 | MSH6 | -1.643 | 0.358 | 12 | 0.0006 | 0.0094 | 3.12 | Down |
| 29338 | IL1B | -1.640 | 0.321 | 12 | 0.0003 | 0.0068 | 3.12 | Down |
| 37361 | ATP6V1D | -1.638 | 0.367 | 12 | 0.0008 | 0.0101 | 3.11 | Down |
| 378 | PSMD12 | -1.636 | 0.370 | 12 | 0.0008 | 0.0103 | 3.11 | Down |
| 18230 | PTPN11 | -1.635 | 0.346 | 12 | 0.0005 | 0.0088 | 3.11 | Down |
| 5439 | SPTAN1 | -1.628 | 0.329 | 12 | 0.0003 | 0.0075 | 3.09 | Down |
| 50844 | PTPN3 | -1.627 | 0.152 | 12 | <.0001 | <.0001 | 3.09 | Down |
| 39398 | CHD7 | -1.626 | 0.335 | 12 | 0.0004 | 0.0081 | 3.09 | Down |
| 23667 | KIAA0586 | -1.626 | 0.355 | 12 | 0.0006 | 0.0094 | 3.09 | Down |
| 48385 | KIAA0922 | -1.626 | 0.291 | 12 | 0.0001 | 0.0047 | 3.09 | Down |
| 26732 | SDAD1 | -1.617 | 0.324 | 12 | 0.0003 | 0.0073 | 3.07 | Down |
| 43606 | GPATCH4 | -1.615 | 0.261 | 12 | <.0001 | 0.0027 | 3.06 | Down |
| 44636 | RASEF | -1.615 | 0.344 | 12 | 0.0005 | 0.0089 | 3.06 | Down |
| 1240 | NHLRC3 | -1.615 | 0.366 | 12 | 0.0008 | 0.0104 | 3.06 | Down |
| 12343 | KIAA0586 | -1.614 | 0.354 | 12 | 0.0007 | 0.0096 | 3.06 | Down |
| 51652 | PHACTR2 | -1.614 | 0.351 | 12 | 0.0006 | 0.0094 | 3.06 | Down |
| 9401 | PHACTR2 | -1.613 | 0.348 | 12 | 0.0006 | 0.0092 | 3.06 | Down |
| 32644 | KIAA0586 | -1.613 | 0.316 | 12 | 0.0003 | 0.0068 | 3.06 | Down |
| 47492 | HIF1A | -1.607 | 0.367 | 12 | 0.0009 | 0.0106 | 3.05 | Down |
| 3231 | RASAL2 | -1.606 | 0.316 | 12 | 0.0003 | 0.0068 | 3.04 | Down |
| 48201 | ADK | -1.606 | 0.341 | 12 | 0.0005 | 0.0088 | 3.04 | Down |
| 51280 | PTPRK | -1.604 | 0.345 | 12 | 0.0006 | 0.0092 | 3.04 | Down |
| 16332 | HBS1L | -1.604 | 0.341 | 12 | 0.0005 | 0.0089 | 3.04 | Down |
| 60708 | PHACTR2 | -1.602 | 0.266 | 12 | <.0001 | 0.0032 | 3.04 | Down |
| 55028 | AIMP1 | -1.598 | 0.360 | 12 | 0.0008 | 0.0103 | 3.03 | Down |
| 46646 | A_24_P246963 | -1.597 | 0.321 | 12 | 0.0003 | 0.0074 | 3.03 | Down |
| 50635 | PEAK1 | -1.593 | 0.338 | 12 | 0.0005 | 0.0089 | 3.02 | Down |
| 29076 | CCDC80 | -1.593 | 0.304 | 12 | 0.0002 | 0.0061 | 3.02 | Down |
| 20190 | NOL8 | -1.592 | 0.304 | 12 | 0.0002 | 0.0061 | 3.02 | Down |
| 41440 | MSH6 | -1.591 | 0.338 | 12 | 0.0005 | 0.0089 | 3.01 | Down |
| 34505 | C15orf23 | -1.590 | 0.309 | 12 | 0.0002 | 0.0066 | 3.01 | Down |
| 59943 | SRD5A1 | -1.581 | 0.246 | 12 | <.0001 | 0.0022 | 2.99 | Down |
| 3279 | TGS1 | -1.580 | 0.350 | 12 | 0.0007 | 0.0098 | 2.99 | Down |
| 14799 | A_33_P333398 | -1.577 | 0.348 | 12 | 0.0007 | 0.0098 | 2.98 | Down |
| 58210 | KIAA0586 | -1.575 | 0.348 | 12 | 0.0007 | 0.0098 | 2.98 | Down |
| 15414 | CTAGE4 | -1.573 | 0.350 | 12 | 0.0007 | 0.0100 | 2.97 | Down |
| 1692 | GTPBP4 | -1.572 | 0.345 | 12 | 0.0007 | 0.0097 | 2.97 | Down |
| 33839 | XLOC_005081 | -1.571 | 0.286 | 12 | 0.0001 | 0.0051 | 2.97 | Down |
| 4645 | PHACTR2 | -1.569 | 0.349 | 12 | 0.0007 | 0.0100 | 2.97 | Down |
| 8851 | CLIP4 | -1.567 | 0.279 | 12 | 0.0001 | 0.0046 | 2.96 | Down |
| 556 | AMD1 | -1.563 | 0.361 | 12 | 0.0010 | 0.0109 | 2.96 | Down |
| 24676 | TUG1 | -1.562 | 0.351 | 12 | 0.0008 | 0.0101 | 2.95 | Down |
| 29080 | CBWD5 | -1.560 | 0.351 | 12 | 0.0008 | 0.0103 | 2.95 | Down |
| 41874 | ZNF420 | -1.559 | 0.312 | 12 | 0.0003 | 0.0073 | 2.95 | Down |
| 51607 | PPIP5K1 | -1.555 | 0.348 | 12 | 0.0008 | 0.0100 | 2.94 | Down |
| 56781 | YME1L1 | -1.554 | 0.351 | 12 | 0.0008 | 0.0103 | 2.94 | Down |
| 5607 | SORBS2 | -1.539 | 0.270 | 12 | <.0001 | 0.0043 | 2.91 | Down |
| 32910 | A_24_P126691 | -1.539 | 0.354 | 12 | 0.0010 | 0.0108 | 2.91 | Down |
| 1348 | EXOC5 | -1.536 | 0.259 | 12 | <.0001 | 0.0035 | 2.90 | Down |
| 43723 | HSP90AA1 | -1.531 | 0.350 | 12 | 0.0009 | 0.0107 | 2.89 | Down |
| 12588 | SHQ1 | -1.529 | 0.347 | 12 | 0.0009 | 0.0104 | 2.89 | Down |
| 14282 | PAK1IP1 | -1.528 | 0.348 | 12 | 0.0009 | 0.0106 | 2.88 | Down |
| 13060 | PAK1IP1 | -1.527 | 0.354 | 12 | 0.0010 | 0.0111 | 2.88 | Down |
| 5430 | KIAA0430 | -1.521 | 0.278 | 12 | 0.0001 | 0.0052 | 2.87 | Down |
| 36161 | GALNT1 | -1.519 | 0.351 | 12 | 0.0010 | 0.0110 | 2.87 | Down |
| 19748 | TAF1 | -1.512 | 0.280 | 12 | 0.0002 | 0.0054 | 2.85 | Down |
| 56871 | SEPT7 | -1.504 | 0.340 | 12 | 0.0008 | 0.0104 | 2.84 | Down |
| 33213 | BBS4 | -1.503 | 0.325 | 12 | 0.0006 | 0.0092 | 2.84 | Down |
| 24168 | TDRKH | -1.502 | 0.316 | 12 | 0.0005 | 0.0086 | 2.83 | Down |
| 31236 | IGF2BP3 | -1.501 | 0.334 | 12 | 0.0007 | 0.0099 | 2.83 | Down |
| 34183 | DNAJC3 | -1.498 | 0.309 | 12 | 0.0004 | 0.0081 | 2.82 | Down |
| 34645 | ZNF165 | -1.494 | 0.281 | 12 | 0.0002 | 0.0058 | 2.82 | Down |
| 51410 | RRP15 | -1.493 | 0.307 | 12 | 0.0004 | 0.0081 | 2.81 | Down |
| 47836 | C1orf96 | -1.492 | 0.317 | 12 | 0.0005 | 0.0089 | 2.81 | Down |
| 47082 | PPFIBP1 | -1.487 | 0.327 | 12 | 0.0007 | 0.0097 | 2.80 | Down |
| 23244 | DDAH1 | -1.479 | 0.190 | 12 | <.0001 | 0.0007 | 2.79 | Down |
| 1259 | CHD8 | -1.478 | 0.207 | 12 | <.0001 | 0.0012 | 2.79 | Down |
| 14087 | CTCF | -1.478 | 0.340 | 12 | 0.0009 | 0.0108 | 2.78 | Down |
| 11963 | ECI2 | -1.477 | 0.299 | 12 | 0.0003 | 0.0076 | 2.78 | Down |
| 51993 | NFX1 | -1.468 | 0.312 | 12 | 0.0005 | 0.0089 | 2.77 | Down |
| 25236 | PAK1IP1 | -1.468 | 0.335 | 12 | 0.0009 | 0.0106 | 2.77 | Down |
| 13749 | UCHL5 | -1.468 | 0.293 | 12 | 0.0003 | 0.0072 | 2.77 | Down |
| 46142 | GPATCH4 | -1.467 | 0.254 | 12 | <.0001 | 0.0039 | 2.76 | Down |
| 43652 | CSDE1 | -1.467 | 0.266 | 12 | 0.0001 | 0.0050 | 2.76 | Down |
| 42655 | UBR4 | -1.463 | 0.188 | 12 | <.0001 | 0.0007 | 2.76 | Down |
| 62595 | XLOC_l2_0011 | -1.463 | 0.339 | 12 | 0.0010 | 0.0111 | 2.76 | Down |
| 33964 | UGGT2 | -1.461 | 0.247 | 12 | <.0001 | 0.0035 | 2.75 | Down |
| 55612 | WDR3 | -1.460 | 0.312 | 12 | 0.0005 | 0.0090 | 2.75 | Down |
| 59504 | BC014023 | -1.447 | 0.298 | 12 | 0.0004 | 0.0081 | 2.73 | Down |
| 43616 | EIF2B3 | -1.447 | 0.254 | 12 | 0.0001 | 0.0043 | 2.73 | Down |
| 1616 | CEP250 | -1.447 | 0.284 | 12 | 0.0003 | 0.0068 | 2.73 | Down |
| 13974 | RAD21 | -1.439 | 0.333 | 12 | 0.0010 | 0.0111 | 2.71 | Down |
| 28388 | TNRC6B | -1.438 | 0.309 | 12 | 0.0006 | 0.0092 | 2.71 | Down |
| 3349 | FAM96A | -1.423 | 0.096 | 12 | <.0001 | <.0001 | 2.68 | Down |
| 46779 | RSRC2 | -1.418 | 0.325 | 12 | 0.0009 | 0.0107 | 2.67 | Down |
| 8568 | XLOC_l2_0101 | -1.413 | 0.303 | 12 | 0.0006 | 0.0092 | 2.66 | Down |
| 40995 | HUWE1 | -1.411 | 0.237 | 12 | <.0001 | 0.0034 | 2.66 | Down |
| 138 | ERLEC1 | -1.403 | 0.292 | 12 | 0.0004 | 0.0083 | 2.64 | Down |
| 51572 | HDLBP | -1.402 | 0.275 | 12 | 0.0003 | 0.0068 | 2.64 | Down |
| 34752 | ATG10 | -1.400 | 0.313 | 12 | 0.0008 | 0.0100 | 2.64 | Down |
| 33113 | EIF2B3 | -1.398 | 0.221 | 12 | <.0001 | 0.0024 | 2.64 | Down |
| 11767 | A_24_P152983 | -1.391 | 0.273 | 12 | 0.0003 | 0.0068 | 2.62 | Down |
| 39065 | RALGAPB | -1.386 | 0.319 | 12 | 0.0009 | 0.0108 | 2.61 | Down |
| 52665 | SORL1 | -1.382 | 0.252 | 12 | 0.0001 | 0.0052 | 2.61 | Down |
| 62785 | TPX2 | -1.382 | 0.238 | 12 | <.0001 | 0.0039 | 2.61 | Down |
| 58794 | HSP90AB1 | -1.381 | 0.272 | 12 | 0.0003 | 0.0069 | 2.60 | Down |
| 31827 | CTNNA1 | -1.380 | 0.254 | 12 | 0.0002 | 0.0053 | 2.60 | Down |
| 32241 | TTLL1 | -1.378 | 0.201 | 12 | <.0001 | 0.0015 | 2.60 | Down |
| 33783 | MTIF3 | -1.370 | 0.314 | 12 | 0.0009 | 0.0107 | 2.58 | Down |
| 19916 | ZNF462 | -1.364 | 0.176 | 12 | <.0001 | 0.0007 | 2.57 | Down |
| 30873 | FHOD3 | -1.361 | 0.313 | 12 | 0.0010 | 0.0109 | 2.57 | Down |
| 45353 | MRPS31 | -1.357 | 0.310 | 12 | 0.0009 | 0.0106 | 2.56 | Down |
| 48273 | TTF2 | -1.350 | 0.301 | 12 | 0.0007 | 0.0100 | 2.55 | Down |
| 18150 | PSMC1 | -1.348 | 0.291 | 12 | 0.0006 | 0.0093 | 2.55 | Down |
| 54562 | MGEA5 | -1.344 | 0.291 | 12 | 0.0006 | 0.0093 | 2.54 | Down |
| 25656 | GEMIN5 | -1.341 | 0.309 | 12 | 0.0010 | 0.0109 | 2.53 | Down |
| 33575 | CARD6 | -1.339 | 0.299 | 12 | 0.0008 | 0.0100 | 2.53 | Down |
| 17115 | KIAA0020 | -1.336 | 0.292 | 12 | 0.0006 | 0.0095 | 2.52 | Down |
| 53765 | PARD3 | -1.319 | 0.256 | 12 | 0.0002 | 0.0066 | 2.50 | Down |
| 56541 | CCNB1 | -1.315 | 0.216 | 12 | <.0001 | 0.0030 | 2.49 | Down |
| 54544 | COIL | -1.313 | 0.293 | 12 | 0.0008 | 0.0100 | 2.48 | Down |
| 16690 | RSC1A1 | -1.296 | 0.264 | 12 | 0.0004 | 0.0077 | 2.46 | Down |
| 2062 | KDM5B | -1.296 | 0.239 | 12 | 0.0002 | 0.0053 | 2.45 | Down |
| 46035 | PRDM4 | -1.294 | 0.290 | 12 | 0.0008 | 0.0101 | 2.45 | Down |
| 56689 | UCHL5 | -1.292 | 0.294 | 12 | 0.0009 | 0.0105 | 2.45 | Down |
| 24359 | UCHL5 | -1.289 | 0.284 | 12 | 0.0007 | 0.0097 | 2.44 | Down |
| 9713 | SON | -1.287 | 0.291 | 12 | 0.0008 | 0.0103 | 2.44 | Down |
| 29273 | UCHL5 | -1.285 | 0.279 | 12 | 0.0006 | 0.0094 | 2.44 | Down |
| 29245 | KIAA0753 | -1.284 | 0.266 | 12 | 0.0004 | 0.0082 | 2.44 | Down |
| 3856 | PRPF40A | -1.281 | 0.274 | 12 | 0.0005 | 0.0090 | 2.43 | Down |
| 62669 | MTOR | -1.279 | 0.272 | 12 | 0.0005 | 0.0089 | 2.43 | Down |
| 4048 | ZMYND8 | -1.278 | 0.278 | 12 | 0.0006 | 0.0094 | 2.43 | Down |
| 48436 | PITPNB | -1.275 | 0.236 | 12 | 0.0002 | 0.0054 | 2.42 | Down |
| 15584 | ACRC | -1.273 | 0.270 | 12 | 0.0005 | 0.0089 | 2.42 | Down |
| 37932 | IRAK2 | -1.272 | 0.226 | 12 | 0.0001 | 0.0045 | 2.42 | Down |
| 54330 | SF3A3 | -1.264 | 0.256 | 12 | 0.0003 | 0.0075 | 2.40 | Down |
| 46587 | CIT | -1.257 | 0.151 | 12 | <.0001 | 0.0005 | 2.39 | Down |
| 11659 | EIF3L | -1.252 | 0.239 | 12 | 0.0002 | 0.0062 | 2.38 | Down |
| 46302 | UGGT1 | -1.238 | 0.197 | 12 | <.0001 | 0.0025 | 2.36 | Down |
| 2834 | TMEM185B | -1.237 | 0.262 | 12 | 0.0005 | 0.0088 | 2.36 | Down |
| 20901 | ECD | -1.228 | 0.278 | 12 | 0.0008 | 0.0104 | 2.34 | Down |
| 15517 | MRRF | -1.227 | 0.243 | 12 | 0.0003 | 0.0070 | 2.34 | Down |
| 21349 | NBPF15 | -1.224 | 0.271 | 12 | 0.0007 | 0.0098 | 2.34 | Down |
| 19261 | C22orf13 | -1.223 | 0.267 | 12 | 0.0006 | 0.0095 | 2.33 | Down |
| 59593 | DNM3 | -1.223 | 0.276 | 12 | 0.0008 | 0.0103 | 2.33 | Down |
| 34345 | BRD8 | -1.223 | 0.238 | 12 | 0.0002 | 0.0066 | 2.33 | Down |
| 24926 | TMEM185B | -1.222 | 0.268 | 12 | 0.0006 | 0.0095 | 2.33 | Down |
| 46839 | DNAJB6 | -1.211 | 0.157 | 12 | <.0001 | 0.0007 | 2.31 | Down |
| 16274 | GON4L | -1.210 | 0.221 | 12 | 0.0001 | 0.0052 | 2.31 | Down |
| 9704 | IL1R1 | -1.210 | 0.267 | 12 | 0.0007 | 0.0098 | 2.31 | Down |
| 10180 | SPECC1L | -1.205 | 0.250 | 12 | 0.0004 | 0.0083 | 2.30 | Down |
| 46811 | TMEM185B | -1.198 | 0.229 | 12 | 0.0002 | 0.0062 | 2.29 | Down |
| 31780 | ZC3H11A | -1.195 | 0.273 | 12 | 0.0009 | 0.0106 | 2.29 | Down |
| 16607 | TUG1 | -1.195 | 0.193 | 12 | <.0001 | 0.0027 | 2.29 | Down |
| 8051 | CHD4 | -1.191 | 0.250 | 12 | 0.0005 | 0.0085 | 2.28 | Down |
| 16368 | ZNF398 | -1.190 | 0.217 | 12 | 0.0001 | 0.0051 | 2.28 | Down |
| 1523 | FAM114A1 | -1.188 | 0.248 | 12 | 0.0004 | 0.0084 | 2.28 | Down |
| 51639 | TMEM185B | -1.170 | 0.269 | 12 | 0.0009 | 0.0108 | 2.25 | Down |
| 9725 | RNF170 | -1.169 | 0.269 | 12 | 0.0009 | 0.0108 | 2.25 | Down |
| 20403 | TMEM185B | -1.169 | 0.259 | 12 | 0.0007 | 0.0098 | 2.25 | Down |
| 4368 | SRGAP1 | -1.167 | 0.232 | 12 | 0.0003 | 0.0072 | 2.24 | Down |
| 50726 | PKN2 | -1.165 | 0.237 | 12 | 0.0004 | 0.0077 | 2.24 | Down |
| 27013 | DHX8 | -1.163 | 0.227 | 12 | 0.0003 | 0.0067 | 2.24 | Down |
| 41102 | DIAPH3 | -1.144 | 0.214 | 12 | 0.0002 | 0.0057 | 2.21 | Down |
| 50667 | ZZEF1 | -1.134 | 0.205 | 12 | 0.0001 | 0.0050 | 2.20 | Down |
| 20813 | XLOC_l2_0082 | -1.125 | 0.237 | 12 | 0.0005 | 0.0086 | 2.18 | Down |
| 46497 | WDR70 | -1.123 | 0.219 | 12 | 0.0003 | 0.0067 | 2.18 | Down |
| 43865 | NIPAL3 | -1.120 | 0.247 | 12 | 0.0007 | 0.0097 | 2.17 | Down |
| 52118 | PDCD11 | -1.119 | 0.239 | 12 | 0.0005 | 0.0089 | 2.17 | Down |
| 310 | HSP90AB1 | -1.116 | 0.202 | 12 | 0.0001 | 0.0050 | 2.17 | Down |
| 45050 | UTP11L | -1.105 | 0.236 | 12 | 0.0005 | 0.0090 | 2.15 | Down |
| 35494 | TMEM185B | -1.095 | 0.207 | 12 | 0.0002 | 0.0059 | 2.14 | Down |
| 60878 | UTP11L | -1.090 | 0.240 | 12 | 0.0007 | 0.0097 | 2.13 | Down |
| 29265 | UBL4A | -1.087 | 0.207 | 12 | 0.0002 | 0.0061 | 2.12 | Down |
| 45069 | TUG1 | -1.080 | 0.235 | 12 | 0.0006 | 0.0094 | 2.11 | Down |
| 11926 | MLL | -1.079 | 0.210 | 12 | 0.0002 | 0.0066 | 2.11 | Down |
| 32400 | KCTD14 | -1.079 | 0.245 | 12 | 0.0009 | 0.0105 | 2.11 | Down |
| 45277 | TULP4 | -1.074 | 0.219 | 12 | 0.0004 | 0.0078 | 2.11 | Down |
| 29869 | HUWE1 | -1.068 | 0.207 | 12 | 0.0002 | 0.0066 | 2.10 | Down |
| 15402 | SNRNP200 | -1.067 | 0.168 | 12 | <.0001 | 0.0023 | 2.10 | Down |
| 25282 | NFX1 | -1.055 | 0.227 | 12 | 0.0006 | 0.0092 | 2.08 | Down |
| 25638 | XLOC_000822 | -1.055 | 0.240 | 12 | 0.0009 | 0.0105 | 2.08 | Down |
| 45722 | TBCE | -1.048 | 0.196 | 12 | 0.0002 | 0.0056 | 2.07 | Down |
| 32533 | NBPF10 | -1.041 | 0.204 | 12 | 0.0003 | 0.0068 | 2.06 | Down |
| 35790 | HERC2 | -1.035 | 0.156 | 12 | <.0001 | 0.0018 | 2.05 | Down |
| 58485 | GLG1 | -1.034 | 0.168 | 12 | <.0001 | 0.0027 | 2.05 | Down |
| 61583 | HSP90AB5P | -1.034 | 0.165 | 12 | <.0001 | 0.0025 | 2.05 | Down |
| 28269 | LRRFIP1 | -1.030 | 0.198 | 12 | 0.0002 | 0.0064 | 2.04 | Down |
| 11935 | SPEN | -1.019 | 0.229 | 12 | 0.0008 | 0.0102 | 2.03 | Down |
| 37148 | GPATCH3 | -1.013 | 0.132 | 12 | <.0001 | 0.0008 | 2.02 | Down |
| 13427 | A_19_P008089 | -1.011 | 0.214 | 12 | 0.0005 | 0.0087 | 2.02 | Down |

| **FeatureNum** | **GeneName** | **Estimate** | **Standard Error** | **DF** | **Pr > \|t\|** | **False Discovery Rate p-value** | **Fold Change** | **Direction of Change** |
| --- | --- | --- | --- | --- | --- | --- | --- | --- |
| 46231 | SNORA19 | 3.587 | 0.822 | 12 | 0.0009 | 0.0107 | 12.02 | UP |
| 5986 | SNORD15A | 3.190 | 0.724 | 12 | 0.0009 | 0.0104 | 9.13 | UP |
| 61454 | EMP2 | 2.943 | 0.203 | 12 | <.0001 | <.0001 | 7.69 | UP |
| 55350 | CHPF | 2.903 | 0.415 | 12 | <.0001 | 0.0014 | 7.48 | UP |
| 11416 | SNORA11D | 2.871 | 0.665 | 12 | 0.0010 | 0.0111 | 7.31 | UP |
| 37289 | TOP1MT | 2.863 | 0.289 | 12 | <.0001 | 0.0001 | 7.27 | UP |
| 60802 | EMP2 | 2.848 | 0.177 | 12 | <.0001 | <.0001 | 7.20 | UP |
| 24591 | EMP2 | 2.837 | 0.161 | 12 | <.0001 | <.0001 | 7.14 | UP |
| 10787 | EMP2 | 2.825 | 0.169 | 12 | <.0001 | <.0001 | 7.09 | UP |
| 1161 | EMP2 | 2.789 | 0.162 | 12 | <.0001 | <.0001 | 6.91 | UP |
| 5256 | EMP2 | 2.781 | 0.155 | 12 | <.0001 | <.0001 | 6.87 | UP |
| 52005 | EMP2 | 2.780 | 0.169 | 12 | <.0001 | <.0001 | 6.87 | UP |
| 12227 | EMP2 | 2.773 | 0.162 | 12 | <.0001 | <.0001 | 6.84 | UP |
| 36988 | EMP2 | 2.721 | 0.173 | 12 | <.0001 | <.0001 | 6.60 | UP |
| 28677 | A_33_P321056 | 2.709 | 0.125 | 12 | <.0001 | <.0001 | 6.54 | UP |
| 34987 | EMP2 | 2.704 | 0.170 | 12 | <.0001 | <.0001 | 6.52 | UP |
| 38049 | SLC41A1 | 2.578 | 0.185 | 12 | <.0001 | <.0001 | 5.97 | UP |
| 9897 | SNORA34 | 2.508 | 0.541 | 12 | 0.0006 | 0.0092 | 5.69 | UP |
| 19200 | SNORA54 | 2.453 | 0.522 | 12 | 0.0005 | 0.0089 | 5.48 | UP |
| 49352 | NID1 | 2.419 | 0.272 | 12 | <.0001 | 0.0003 | 5.35 | UP |
| 57835 | PPP1R14B | 2.370 | 0.531 | 12 | 0.0008 | 0.0101 | 5.17 | UP |
| 9 | SNAR-G2 | 2.347 | 0.536 | 12 | 0.0009 | 0.0106 | 5.09 | UP |
| 41187 | ILF3 | 2.310 | 0.153 | 12 | <.0001 | <.0001 | 4.96 | UP |
| 56619 | PHF1 | 2.310 | 0.333 | 12 | <.0001 | 0.0014 | 4.96 | UP |
| 21237 | PPP1R14B | 2.304 | 0.468 | 12 | 0.0004 | 0.0077 | 4.94 | UP |
| 39302 | RMRP | 2.282 | 0.478 | 12 | 0.0005 | 0.0085 | 4.86 | UP |
| 11845 | EMP2 | 2.251 | 0.154 | 12 | <.0001 | <.0001 | 4.76 | UP |
| 14478 | MRPS6 | 2.246 | 0.331 | 12 | <.0001 | 0.0017 | 4.74 | UP |
| 26634 | MRPS6 | 2.243 | 0.338 | 12 | <.0001 | 0.0018 | 4.73 | UP |
| 48033 | SNORA11C | 2.229 | 0.505 | 12 | 0.0008 | 0.0104 | 4.69 | UP |
| 45561 | MRPS6 | 2.226 | 0.333 | 12 | <.0001 | 0.0018 | 4.68 | UP |
| 10019 | ZFP36L2 | 2.223 | 0.487 | 12 | 0.0006 | 0.0095 | 4.67 | UP |
| 59889 | ZFP36L2 | 2.219 | 0.461 | 12 | 0.0004 | 0.0083 | 4.66 | UP |
| 27586 | MRPS6 | 2.211 | 0.331 | 12 | <.0001 | 0.0018 | 4.63 | UP |
| 29805 | BASP1 | 2.210 | 0.316 | 12 | <.0001 | 0.0014 | 4.63 | UP |
| 17435 | MRPS6 | 2.208 | 0.302 | 12 | <.0001 | 0.0010 | 4.62 | UP |
| 26111 | MRPS6 | 2.204 | 0.330 | 12 | <.0001 | 0.0018 | 4.61 | UP |
| 34151 | MRPS6 | 2.198 | 0.331 | 12 | <.0001 | 0.0018 | 4.59 | UP |
| 25589 | ZFP36L2 | 2.185 | 0.457 | 12 | 0.0004 | 0.0084 | 4.55 | UP |
| 31024 | MRPS6 | 2.184 | 0.338 | 12 | <.0001 | 0.0021 | 4.55 | UP |
| 176 | ZFP36L2 | 2.180 | 0.493 | 12 | 0.0008 | 0.0103 | 4.53 | UP |
| 8385 | ZFP36L2 | 2.178 | 0.470 | 12 | 0.0006 | 0.0092 | 4.52 | UP |
| 54480 | MRPS6 | 2.175 | 0.330 | 12 | <.0001 | 0.0019 | 4.52 | UP |
| 6060 | ZFP36L2 | 2.163 | 0.456 | 12 | 0.0005 | 0.0086 | 4.48 | UP |
| 46662 | MRPS6 | 2.155 | 0.323 | 12 | <.0001 | 0.0018 | 4.45 | UP |
| 60502 | HMHA1 | 2.155 | 0.415 | 12 | 0.0002 | 0.0064 | 4.45 | UP |
| 29614 | ZFP36L2 | 2.150 | 0.447 | 12 | 0.0004 | 0.0083 | 4.44 | UP |
| 39223 | LBH | 2.139 | 0.340 | 12 | <.0001 | 0.0024 | 4.40 | UP |
| 37263 | LBH | 2.135 | 0.328 | 12 | <.0001 | 0.0021 | 4.39 | UP |
| 41063 | LBH | 2.124 | 0.358 | 12 | <.0001 | 0.0034 | 4.36 | UP |
| 48676 | YWHAH | 2.113 | 0.151 | 12 | <.0001 | <.0001 | 4.33 | UP |
| 5666 | LBH | 2.108 | 0.306 | 12 | <.0001 | 0.0015 | 4.31 | UP |
| 26222 | VWA5B2 | 2.106 | 0.351 | 12 | <.0001 | 0.0032 | 4.30 | UP |
| 28261 | ZFP36L2 | 2.105 | 0.474 | 12 | 0.0008 | 0.0102 | 4.30 | UP |
| 8538 | YWHAH | 2.103 | 0.156 | 12 | <.0001 | <.0001 | 4.30 | UP |
| 20360 | LBH | 2.098 | 0.310 | 12 | <.0001 | 0.0017 | 4.28 | UP |
| 55457 | LBH | 2.096 | 0.331 | 12 | <.0001 | 0.0024 | 4.27 | UP |
| 6477 | YWHAH | 2.095 | 0.158 | 12 | <.0001 | <.0001 | 4.27 | UP |
| 26359 | ZFP36L2 | 2.084 | 0.451 | 12 | 0.0006 | 0.0093 | 4.24 | UP |
| 18305 | YWHAH | 2.082 | 0.160 | 12 | <.0001 | <.0001 | 4.23 | UP |
| 46066 | GNB1 | 2.077 | 0.122 | 12 | <.0001 | <.0001 | 4.22 | UP |
| 29913 | YWHAH | 2.070 | 0.186 | 12 | <.0001 | <.0001 | 4.20 | UP |
| 54970 | NT5DC2 | 2.068 | 0.263 | 12 | <.0001 | 0.0007 | 4.19 | UP |
| 62892 | ZFP36L2 | 2.066 | 0.447 | 12 | 0.0006 | 0.0093 | 4.19 | UP |
| 50266 | YWHAH | 2.064 | 0.180 | 12 | <.0001 | <.0001 | 4.18 | UP |
| 23763 | LBH | 2.063 | 0.305 | 12 | <.0001 | 0.0017 | 4.18 | UP |
| 13657 | PRRC2B | 2.060 | 0.401 | 12 | 0.0002 | 0.0066 | 4.17 | UP |
| 40874 | YWHAH | 2.055 | 0.180 | 12 | <.0001 | <.0001 | 4.16 | UP |
| 18723 | LBH | 2.053 | 0.350 | 12 | <.0001 | 0.0037 | 4.15 | UP |
| 44453 | YWHAH | 2.049 | 0.167 | 12 | <.0001 | <.0001 | 4.14 | UP |
| 42970 | YWHAH | 2.047 | 0.154 | 12 | <.0001 | <.0001 | 4.13 | UP |
| 55927 | LBH | 2.040 | 0.219 | 12 | <.0001 | 0.0002 | 4.11 | UP |
| 55977 | FOXD1 | 2.039 | 0.407 | 12 | 0.0003 | 0.0072 | 4.11 | UP |
| 42327 | MOGS | 2.031 | 0.335 | 12 | <.0001 | 0.0031 | 4.09 | UP |
| 27588 | LBH | 2.028 | 0.396 | 12 | 0.0003 | 0.0067 | 4.08 | UP |
| 21700 | SNORD16 | 2.018 | 0.435 | 12 | 0.0006 | 0.0092 | 4.05 | UP |
| 917 | YWHAH | 2.018 | 0.129 | 12 | <.0001 | <.0001 | 4.05 | UP |
| 45738 | CNOT3 | 2.010 | 0.341 | 12 | <.0001 | 0.0035 | 4.03 | UP |
| 45710 | ANO10 | 2.002 | 0.077 | 12 | <.0001 | <.0001 | 4.01 | UP |
| 10423 | EFNB1 | 1.993 | 0.414 | 12 | 0.0004 | 0.0083 | 3.98 | UP |
| 24808 | TMEM191A | 1.949 | 0.426 | 12 | 0.0006 | 0.0095 | 3.86 | UP |
| 34233 | THG1L | 1.933 | 0.211 | 12 | <.0001 | 0.0003 | 3.82 | UP |
| 12299 | SH3PXD2A | 1.932 | 0.223 | 12 | <.0001 | 0.0004 | 3.82 | UP |
| 11801 | TMEM184B | 1.898 | 0.349 | 12 | 0.0002 | 0.0053 | 3.73 | UP |
| 11062 | ZBTB47 | 1.889 | 0.223 | 12 | <.0001 | 0.0005 | 3.71 | UP |
| 14268 | THG1L | 1.869 | 0.218 | 12 | <.0001 | 0.0004 | 3.65 | UP |
| 57057 | LBH | 1.861 | 0.413 | 12 | 0.0007 | 0.0099 | 3.63 | UP |
| 4018 | PITX1 | 1.838 | 0.340 | 12 | 0.0002 | 0.0053 | 3.58 | UP |
| 18854 | THG1L | 1.828 | 0.254 | 12 | <.0001 | 0.0012 | 3.55 | UP |
| 40137 | CABLES1 | 1.823 | 0.269 | 12 | <.0001 | 0.0017 | 3.54 | UP |
| 10526 | LOC100507002 | 1.816 | 0.275 | 12 | <.0001 | 0.0019 | 3.52 | UP |
| 55288 | THG1L | 1.811 | 0.226 | 12 | <.0001 | 0.0006 | 3.51 | UP |
| 23916 | THG1L | 1.807 | 0.218 | 12 | <.0001 | 0.0005 | 3.50 | UP |
| 41246 | FAM158A | 1.804 | 0.337 | 12 | 0.0002 | 0.0056 | 3.49 | UP |
| 58751 | HS6ST1 | 1.795 | 0.306 | 12 | <.0001 | 0.0037 | 3.47 | UP |
| 57 | TMEM191B | 1.765 | 0.385 | 12 | 0.0006 | 0.0094 | 3.40 | UP |
| 30115 | THG1L | 1.760 | 0.207 | 12 | <.0001 | 0.0004 | 3.39 | UP |
| 30626 | THG1L | 1.757 | 0.211 | 12 | <.0001 | 0.0005 | 3.38 | UP |
| 52756 | THG1L | 1.755 | 0.214 | 12 | <.0001 | 0.0005 | 3.38 | UP |
| 60486 | SEMA4F | 1.728 | 0.286 | 12 | <.0001 | 0.0031 | 3.31 | UP |
| 16347 | STEAP3 | 1.721 | 0.164 | 12 | <.0001 | <.0001 | 3.30 | UP |
| 16931 | NICN1 | 1.717 | 0.325 | 12 | 0.0002 | 0.0059 | 3.29 | UP |
| 62212 | POLR3E | 1.705 | 0.134 | 12 | <.0001 | <.0001 | 3.26 | UP |
| 5886 | UCK1 | 1.698 | 0.167 | 12 | <.0001 | 0.0001 | 3.24 | UP |
| 38199 | RNF144A | 1.690 | 0.220 | 12 | <.0001 | 0.0008 | 3.23 | UP |
| 59066 | FOSL2 | 1.690 | 0.376 | 12 | 0.0007 | 0.0100 | 3.23 | UP |
| 19944 | THG1L | 1.684 | 0.188 | 12 | <.0001 | 0.0003 | 3.21 | UP |
| 23397 | MTFP1 | 1.673 | 0.255 | 12 | <.0001 | 0.0019 | 3.19 | UP |
| 47802 | LMNB2 | 1.661 | 0.372 | 12 | 0.0008 | 0.0101 | 3.16 | UP |
| 15082 | HERPUD2 | 1.654 | 0.189 | 12 | <.0001 | 0.0004 | 3.15 | UP |
| 43048 | RFX2 | 1.645 | 0.245 | 12 | <.0001 | 0.0017 | 3.13 | UP |
| 8179 | RAB15 | 1.616 | 0.264 | 12 | <.0001 | 0.0029 | 3.06 | UP |
| 12333 | CPNE2 | 1.610 | 0.236 | 12 | <.0001 | 0.0016 | 3.05 | UP |
| 61455 | CELF1 | 1.603 | 0.317 | 12 | 0.0003 | 0.0070 | 3.04 | UP |
| 34629 | ANO10 | 1.594 | 0.190 | 12 | <.0001 | 0.0005 | 3.02 | UP |
| 18801 | TMEM191B | 1.586 | 0.320 | 12 | 0.0003 | 0.0075 | 3.00 | UP |
| 47597 | IQSEC1 | 1.581 | 0.168 | 12 | <.0001 | 0.0002 | 2.99 | UP |
| 25419 | SLIRP | 1.570 | 0.337 | 12 | 0.0006 | 0.0091 | 2.97 | UP |
| 4932 | GDF11 | 1.567 | 0.335 | 12 | 0.0005 | 0.0090 | 2.96 | UP |
| 35190 | AIF1L | 1.562 | 0.358 | 12 | 0.0009 | 0.0107 | 2.95 | UP |
| 27558 | RELL1 | 1.560 | 0.120 | 12 | <.0001 | <.0001 | 2.95 | UP |
| 49039 | THG1L | 1.540 | 0.183 | 12 | <.0001 | 0.0005 | 2.91 | UP |
| 16726 | PLEKHM1 | 1.530 | 0.218 | 12 | <.0001 | 0.0014 | 2.89 | UP |
| 35799 | DNMT1 | 1.523 | 0.168 | 12 | <.0001 | 0.0003 | 2.87 | UP |
| 39905 | CNIH4 | 1.521 | 0.269 | 12 | 0.0001 | 0.0044 | 2.87 | UP |
| 35862 | EIF4EBP2 | 1.516 | 0.317 | 12 | 0.0004 | 0.0084 | 2.86 | UP |
| 30859 | RHPN2 | 1.500 | 0.143 | 12 | <.0001 | <.0001 | 2.83 | UP |
| 10340 | SURF1 | 1.499 | 0.343 | 12 | 0.0009 | 0.0107 | 2.83 | UP |
| 10106 | SNX27 | 1.491 | 0.198 | 12 | <.0001 | 0.0009 | 2.81 | UP |
| 24763 | COBRA1 | 1.485 | 0.334 | 12 | 0.0008 | 0.0102 | 2.80 | UP |
| 2656 | C20orf96 | 1.482 | 0.285 | 12 | 0.0002 | 0.0064 | 2.79 | UP |
| 43483 | CCNE1 | 1.473 | 0.326 | 12 | 0.0007 | 0.0098 | 2.78 | UP |
| 28824 | KANK4 | 1.472 | 0.322 | 12 | 0.0006 | 0.0095 | 2.77 | UP |
| 48665 | XLOC_012452 | 1.468 | 0.290 | 12 | 0.0003 | 0.0069 | 2.77 | UP |
| 57163 | ELFN2 | 1.464 | 0.271 | 12 | 0.0002 | 0.0054 | 2.76 | UP |
| 43145 | AK130638 | 1.459 | 0.197 | 12 | <.0001 | 0.0010 | 2.75 | UP |
| 27261 | IL32 | 1.455 | 0.289 | 12 | 0.0003 | 0.0071 | 2.74 | UP |
| 29356 | ULK1 | 1.447 | 0.323 | 12 | 0.0007 | 0.0100 | 2.73 | UP |
| 47735 | UBE2V1 | 1.444 | 0.320 | 12 | 0.0007 | 0.0098 | 2.72 | UP |
| 52727 | C12orf75 | 1.441 | 0.317 | 12 | 0.0007 | 0.0097 | 2.71 | UP |
| 10743 | SNORD8 | 1.437 | 0.305 | 12 | 0.0005 | 0.0089 | 2.71 | UP |
| 27408 | NDUFAF2 | 1.434 | 0.217 | 12 | <.0001 | 0.0018 | 2.70 | UP |
| 48869 | LRRC20 | 1.425 | 0.322 | 12 | 0.0008 | 0.0103 | 2.68 | UP |
| 58881 | PGRMC2 | 1.420 | 0.079 | 12 | <.0001 | <.0001 | 2.68 | UP |
| 16838 | USP39 | 1.413 | 0.254 | 12 | 0.0001 | 0.0048 | 2.66 | UP |
| 24268 | ABTB1 | 1.406 | 0.315 | 12 | 0.0008 | 0.0101 | 2.65 | UP |
| 5882 | ACTN1 | 1.406 | 0.300 | 12 | 0.0005 | 0.0089 | 2.65 | UP |
| 54057 | EIF4EBP2 | 1.397 | 0.302 | 12 | 0.0006 | 0.0093 | 2.63 | UP |
| 27212 | EIF4EBP2 | 1.394 | 0.303 | 12 | 0.0006 | 0.0094 | 2.63 | UP |
| 44058 | POLR3E | 1.385 | 0.154 | 12 | <.0001 | 0.0003 | 2.61 | UP |
| 39764 | ARPC5 | 1.381 | 0.254 | 12 | 0.0002 | 0.0053 | 2.61 | UP |
| 21710 | EIF4EBP2 | 1.376 | 0.285 | 12 | 0.0004 | 0.0082 | 2.59 | UP |
| 10998 | TMEM19 | 1.374 | 0.206 | 12 | <.0001 | 0.0018 | 2.59 | UP |
| 56149 | EIF4EBP2 | 1.372 | 0.274 | 12 | 0.0003 | 0.0072 | 2.59 | UP |
| 34240 | FANCC | 1.371 | 0.229 | 12 | <.0001 | 0.0032 | 2.59 | UP |
| 37487 | EIF4EBP2 | 1.366 | 0.271 | 12 | 0.0003 | 0.0071 | 2.58 | UP |
| 60476 | XLOC_009191 | 1.366 | 0.278 | 12 | 0.0004 | 0.0077 | 2.58 | UP |
| 15696 | RHOBTB2 | 1.363 | 0.273 | 12 | 0.0003 | 0.0073 | 2.57 | UP |
| 40905 | ERH | 1.362 | 0.251 | 12 | 0.0002 | 0.0053 | 2.57 | UP |
| 51865 | LYPD6 | 1.361 | 0.229 | 12 | <.0001 | 0.0034 | 2.57 | UP |
| 39290 | EIF4EBP2 | 1.361 | 0.279 | 12 | 0.0004 | 0.0080 | 2.57 | UP |
| 41991 | BEND3 | 1.361 | 0.191 | 12 | <.0001 | 0.0012 | 2.57 | UP |
| 40259 | EIF4EBP2 | 1.359 | 0.280 | 12 | 0.0004 | 0.0081 | 2.56 | UP |
| 34232 | ATP1B1 | 1.358 | 0.116 | 12 | <.0001 | <.0001 | 2.56 | UP |
| 625 | EIF4EBP2 | 1.357 | 0.305 | 12 | 0.0008 | 0.0102 | 2.56 | UP |
| 19824 | C1D | 1.356 | 0.222 | 12 | <.0001 | 0.0030 | 2.56 | UP |
| 54937 | EIF4EBP2 | 1.342 | 0.281 | 12 | 0.0005 | 0.0085 | 2.53 | UP |
| 28356 | DNMT3A | 1.340 | 0.248 | 12 | 0.0002 | 0.0054 | 2.53 | UP |
| 60793 | RALB | 1.338 | 0.154 | 12 | <.0001 | 0.0004 | 2.53 | UP |
| 19520 | DAZAP2 | 1.333 | 0.237 | 12 | 0.0001 | 0.0046 | 2.52 | UP |
| 37901 | PITX1 | 1.333 | 0.289 | 12 | 0.0006 | 0.0093 | 2.52 | UP |
| 35898 | FAM122B | 1.329 | 0.247 | 12 | 0.0002 | 0.0055 | 2.51 | UP |
| 22035 | TMEM201 | 1.329 | 0.298 | 12 | 0.0008 | 0.0101 | 2.51 | UP |
| 45336 | ENST00000412 | 1.318 | 0.207 | 12 | <.0001 | 0.0023 | 2.49 | UP |
| 18978 | SNRNP27 | 1.316 | 0.169 | 12 | <.0001 | 0.0007 | 2.49 | UP |
| 59908 | EEF2K | 1.315 | 0.266 | 12 | 0.0003 | 0.0075 | 2.49 | UP |
| 54718 | PLAU | 1.308 | 0.295 | 12 | 0.0008 | 0.0103 | 2.48 | UP |
| 43741 | CHST15 | 1.307 | 0.186 | 12 | <.0001 | 0.0013 | 2.47 | UP |
| 49712 | MXI1 | 1.304 | 0.219 | 12 | <.0001 | 0.0033 | 2.47 | UP |
| 45127 | EIF4E2 | 1.299 | 0.239 | 12 | 0.0001 | 0.0052 | 2.46 | UP |
| 37716 | CCDC149 | 1.294 | 0.296 | 12 | 0.0009 | 0.0107 | 2.45 | UP |
| 37859 | SEC61G | 1.287 | 0.271 | 12 | 0.0005 | 0.0086 | 2.44 | UP |
| 9330 | CKLF | 1.285 | 0.284 | 12 | 0.0007 | 0.0098 | 2.44 | UP |
| 28319 | C19orf77 | 1.281 | 0.291 | 12 | 0.0009 | 0.0104 | 2.43 | UP |
| 5743 | CKS1B | 1.281 | 0.269 | 12 | 0.0005 | 0.0085 | 2.43 | UP |
| 54461 | HOXA5 | 1.270 | 0.194 | 12 | <.0001 | 0.0019 | 2.41 | UP |
| 38450 | PRRC2B | 1.269 | 0.199 | 12 | <.0001 | 0.0023 | 2.41 | UP |
| 38180 | FTSJ1 | 1.267 | 0.132 | 12 | <.0001 | 0.0002 | 2.41 | UP |
| 30043 | A_33_P329221 | 1.266 | 0.272 | 12 | 0.0006 | 0.0092 | 2.40 | UP |
| 40789 | AK4 | 1.266 | 0.196 | 12 | <.0001 | 0.0021 | 2.40 | UP |
| 34287 | ID2 | 1.265 | 0.231 | 12 | 0.0001 | 0.0052 | 2.40 | UP |
| 45725 | TOMM34 | 1.257 | 0.261 | 12 | 0.0004 | 0.0083 | 2.39 | UP |
| 53301 | PSMD9 | 1.253 | 0.288 | 12 | 0.0009 | 0.0108 | 2.38 | UP |
| 20964 | YBX1 | 1.251 | 0.289 | 12 | 0.0010 | 0.0110 | 2.38 | UP |
| 40824 | SERPINB6 | 1.251 | 0.241 | 12 | 0.0002 | 0.0065 | 2.38 | UP |
| 41844 | IAH1 | 1.250 | 0.113 | 12 | <.0001 | <.0001 | 2.38 | UP |
| 49083 | HNRNPUL1 | 1.244 | 0.226 | 12 | 0.0001 | 0.0050 | 2.37 | UP |
| 850 | PLAU | 1.242 | 0.287 | 12 | 0.0010 | 0.0110 | 2.36 | UP |
| 25842 | ARID1B | 1.241 | 0.140 | 12 | <.0001 | 0.0003 | 2.36 | UP |
| 47649 | CCDC23 | 1.238 | 0.270 | 12 | 0.0006 | 0.0094 | 2.36 | UP |
| 40274 | DEXI | 1.228 | 0.280 | 12 | 0.0009 | 0.0106 | 2.34 | UP |
| 56126 | A_33_P336130 | 1.228 | 0.223 | 12 | 0.0001 | 0.0051 | 2.34 | UP |
| 44332 | TBC1D7 | 1.227 | 0.209 | 12 | <.0001 | 0.0037 | 2.34 | UP |
| 507 | SUMO2 | 1.225 | 0.277 | 12 | 0.0008 | 0.0103 | 2.34 | UP |
| 17644 | GNG10 | 1.224 | 0.180 | 12 | <.0001 | 0.0017 | 2.34 | UP |
| 55768 | TBC1D7 | 1.223 | 0.135 | 12 | <.0001 | 0.0003 | 2.33 | UP |
| 59387 | LOC646278 | 1.222 | 0.273 | 12 | 0.0008 | 0.0101 | 2.33 | UP |
| 7755 | HNRNPD | 1.221 | 0.224 | 12 | 0.0001 | 0.0052 | 2.33 | UP |
| 22383 | TMEM191B | 1.220 | 0.243 | 12 | 0.0003 | 0.0072 | 2.33 | UP |
| 45728 | ADCY9 | 1.213 | 0.179 | 12 | <.0001 | 0.0017 | 2.32 | UP |
| 50437 | FOXQ1 | 1.213 | 0.245 | 12 | 0.0003 | 0.0075 | 2.32 | UP |
| 54344 | INHBB | 1.210 | 0.277 | 12 | 0.0009 | 0.0107 | 2.31 | UP |
| 20164 | AIG1 | 1.209 | 0.264 | 12 | 0.0006 | 0.0094 | 2.31 | UP |
| 41892 | ILF3 | 1.203 | 0.151 | 12 | <.0001 | 0.0006 | 2.30 | UP |
| 33441 | NMU | 1.203 | 0.256 | 12 | 0.0005 | 0.0089 | 2.30 | UP |
| 8492 | COQ4 | 1.202 | 0.278 | 12 | 0.0010 | 0.0110 | 2.30 | UP |
| 43870 | CNNM4 | 1.202 | 0.278 | 12 | 0.0010 | 0.0110 | 2.30 | UP |
| 35449 | RASGRP2 | 1.201 | 0.267 | 12 | 0.0007 | 0.0100 | 2.30 | UP |
| 28507 | CS | 1.201 | 0.254 | 12 | 0.0005 | 0.0088 | 2.30 | UP |
| 43369 | CASC3 | 1.198 | 0.193 | 12 | <.0001 | 0.0027 | 2.29 | UP |
| 41608 | PLAU | 1.192 | 0.265 | 12 | 0.0007 | 0.0100 | 2.28 | UP |
| 34944 | LOC100287509 | 1.190 | 0.219 | 12 | 0.0002 | 0.0053 | 2.28 | UP |
| 26796 | TBC1D7 | 1.188 | 0.146 | 12 | <.0001 | 0.0006 | 2.28 | UP |
| 59996 | WWC3 | 1.187 | 0.264 | 12 | 0.0007 | 0.0100 | 2.28 | UP |
| 19523 | OIP5 | 1.186 | 0.094 | 12 | <.0001 | <.0001 | 2.28 | UP |
| 34685 | RAB12 | 1.176 | 0.269 | 12 | 0.0009 | 0.0106 | 2.26 | UP |
| 40334 | PPIC | 1.170 | 0.170 | 12 | <.0001 | 0.0015 | 2.25 | UP |
| 42324 | SET | 1.170 | 0.195 | 12 | <.0001 | 0.0032 | 2.25 | UP |
| 30895 | EIF3B | 1.167 | 0.227 | 12 | 0.0002 | 0.0066 | 2.25 | UP |
| 11530 | RBBP4 | 1.165 | 0.145 | 12 | <.0001 | 0.0006 | 2.24 | UP |
| 34425 | PLAU | 1.162 | 0.263 | 12 | 0.0008 | 0.0103 | 2.24 | UP |
| 38485 | GNL3L | 1.158 | 0.184 | 12 | <.0001 | 0.0025 | 2.23 | UP |
| 56985 | RAB3IL1 | 1.151 | 0.206 | 12 | 0.0001 | 0.0047 | 2.22 | UP |
| 56611 | NDUFA6 | 1.149 | 0.242 | 12 | 0.0005 | 0.0086 | 2.22 | UP |
| 17080 | ATP5J | 1.147 | 0.240 | 12 | 0.0004 | 0.0084 | 2.21 | UP |
| 1449 | CHAF1A | 1.146 | 0.183 | 12 | <.0001 | 0.0025 | 2.21 | UP |
| 58132 | ARPC3 | 1.145 | 0.234 | 12 | 0.0004 | 0.0079 | 2.21 | UP |
| 36036 | RHOQ | 1.144 | 0.169 | 12 | <.0001 | 0.0017 | 2.21 | UP |
| 24801 | TBC1D7 | 1.142 | 0.141 | 12 | <.0001 | 0.0006 | 2.21 | UP |
| 37914 | MAP3K3 | 1.141 | 0.261 | 12 | 0.0009 | 0.0107 | 2.21 | UP |
| 42534 | QKI | 1.140 | 0.150 | 12 | <.0001 | 0.0008 | 2.20 | UP |
| 53896 | AKAP1 | 1.139 | 0.147 | 12 | <.0001 | 0.0007 | 2.20 | UP |
| 38747 | SNRPE | 1.137 | 0.231 | 12 | 0.0004 | 0.0077 | 2.20 | UP |
| 17728 | PHF13 | 1.137 | 0.259 | 12 | 0.0009 | 0.0105 | 2.20 | UP |
| 28901 | TOB1 | 1.136 | 0.180 | 12 | <.0001 | 0.0024 | 2.20 | UP |
| 17301 | VGLL4 | 1.135 | 0.195 | 12 | <.0001 | 0.0038 | 2.20 | UP |
| 5431 | TBC1D7 | 1.134 | 0.135 | 12 | <.0001 | 0.0005 | 2.19 | UP |
| 33144 | CCDC149 | 1.133 | 0.201 | 12 | 0.0001 | 0.0046 | 2.19 | UP |
| 51977 | MAGOH | 1.130 | 0.185 | 12 | <.0001 | 0.0030 | 2.19 | UP |
| 30172 | EIF3B | 1.127 | 0.233 | 12 | 0.0004 | 0.0081 | 2.18 | UP |
| 13504 | PITPNA | 1.125 | 0.146 | 12 | <.0001 | 0.0007 | 2.18 | UP |
| 23980 | LMO2 | 1.123 | 0.221 | 12 | 0.0003 | 0.0069 | 2.18 | UP |
| 6496 | MRPS36 | 1.118 | 0.205 | 12 | 0.0001 | 0.0052 | 2.17 | UP |
| 28637 | LOC730020 | 1.118 | 0.148 | 12 | <.0001 | 0.0009 | 2.17 | UP |
| 28304 | RNPS1 | 1.117 | 0.252 | 12 | 0.0008 | 0.0103 | 2.17 | UP |
| 12739 | COX7B | 1.116 | 0.226 | 12 | 0.0003 | 0.0075 | 2.17 | UP |
| 38767 | PMEPA1 | 1.115 | 0.163 | 12 | <.0001 | 0.0015 | 2.17 | UP |
| 25400 | PXK | 1.114 | 0.218 | 12 | 0.0003 | 0.0068 | 2.16 | UP |
| 35509 | NR2F1 | 1.114 | 0.238 | 12 | 0.0005 | 0.0090 | 2.16 | UP |
| 38969 | IFNAR2 | 1.114 | 0.208 | 12 | 0.0002 | 0.0056 | 2.16 | UP |
| 36122 | USP39 | 1.112 | 0.165 | 12 | <.0001 | 0.0017 | 2.16 | UP |
| 14740 | FAM25A | 1.110 | 0.257 | 12 | 0.0010 | 0.0110 | 2.16 | UP |
| 17920 | EPN2 | 1.104 | 0.254 | 12 | 0.0010 | 0.0109 | 2.15 | UP |
| 6772 | ASAP3 | 1.103 | 0.247 | 12 | 0.0008 | 0.0101 | 2.15 | UP |
| 52606 | TBC1D7 | 1.098 | 0.165 | 12 | <.0001 | 0.0018 | 2.14 | UP |
| 34561 | ARPC2 | 1.098 | 0.227 | 12 | 0.0004 | 0.0081 | 2.14 | UP |
| 36432 | ZNF618 | 1.094 | 0.164 | 12 | <.0001 | 0.0018 | 2.13 | UP |
| 11065 | AP3S1 | 1.092 | 0.214 | 12 | 0.0003 | 0.0068 | 2.13 | UP |
| 17790 | LMO2 | 1.090 | 0.207 | 12 | 0.0002 | 0.0060 | 2.13 | UP |
| 43193 | TACC2 | 1.090 | 0.135 | 12 | <.0001 | 0.0006 | 2.13 | UP |
| 37408 | EGLN1 | 1.087 | 0.140 | 12 | <.0001 | 0.0007 | 2.12 | UP |
| 30938 | MRPS21 | 1.085 | 0.205 | 12 | 0.0002 | 0.0059 | 2.12 | UP |
| 60108 | METTL16 | 1.082 | 0.161 | 12 | <.0001 | 0.0017 | 2.12 | UP |
| 20103 | LMO2 | 1.080 | 0.193 | 12 | 0.0001 | 0.0046 | 2.11 | UP |
| 9939 | XLOC_011837 | 1.075 | 0.171 | 12 | <.0001 | 0.0024 | 2.11 | UP |
| 42496 | SNRPE | 1.074 | 0.190 | 12 | 0.0001 | 0.0044 | 2.10 | UP |
| 33310 | ALG8 | 1.073 | 0.135 | 12 | <.0001 | 0.0006 | 2.10 | UP |
| 60124 | TBC1D7 | 1.073 | 0.146 | 12 | <.0001 | 0.0010 | 2.10 | UP |
| 16659 | TMEM52 | 1.071 | 0.226 | 12 | 0.0005 | 0.0086 | 2.10 | UP |
| 36795 | ZNF516 | 1.067 | 0.195 | 12 | 0.0001 | 0.0052 | 2.09 | UP |
| 50519 | TBC1D7 | 1.066 | 0.149 | 12 | <.0001 | 0.0012 | 2.09 | UP |
| 3729 | MIS18A | 1.066 | 0.102 | 12 | <.0001 | 0.0001 | 2.09 | UP |
| 28287 | SUMO2 | 1.065 | 0.201 | 12 | 0.0002 | 0.0059 | 2.09 | UP |
| 14535 | MAP3K13 | 1.064 | 0.126 | 12 | <.0001 | 0.0005 | 2.09 | UP |
| 61316 | LOC100133124 | 1.064 | 0.196 | 12 | 0.0002 | 0.0053 | 2.09 | UP |
| 24743 | RAB7A | 1.060 | 0.237 | 12 | 0.0008 | 0.0100 | 2.09 | UP |
| 53323 | LMO2 | 1.060 | 0.229 | 12 | 0.0006 | 0.0093 | 2.08 | UP |
| 19333 | INTS9 | 1.059 | 0.238 | 12 | 0.0008 | 0.0101 | 2.08 | UP |
| 28634 | C17orf39 | 1.058 | 0.163 | 12 | <.0001 | 0.0021 | 2.08 | UP |
| 17177 | SLC6A6 | 1.057 | 0.160 | 12 | <.0001 | 0.0019 | 2.08 | UP |
| 62136 | KIAA0040 | 1.057 | 0.187 | 12 | 0.0001 | 0.0044 | 2.08 | UP |
| 4484 | LMO2 | 1.050 | 0.192 | 12 | 0.0001 | 0.0052 | 2.07 | UP |
| 52735 | PXDN | 1.049 | 0.179 | 12 | <.0001 | 0.0036 | 2.07 | UP |
| 45441 | ENST00000399 | 1.049 | 0.182 | 12 | <.0001 | 0.0040 | 2.07 | UP |
| 55274 | ATP9A | 1.047 | 0.236 | 12 | 0.0008 | 0.0103 | 2.07 | UP |
| 5761 | SPDEF | 1.047 | 0.167 | 12 | <.0001 | 0.0025 | 2.07 | UP |
| 49111 | TBC1D7 | 1.046 | 0.118 | 12 | <.0001 | 0.0003 | 2.06 | UP |
| 3892 | CCDC23 | 1.046 | 0.236 | 12 | 0.0008 | 0.0103 | 2.06 | UP |
| 35733 | SUFU | 1.045 | 0.217 | 12 | 0.0004 | 0.0082 | 2.06 | UP |
| 50797 | TBC1D7 | 1.043 | 0.148 | 12 | <.0001 | 0.0013 | 2.06 | UP |
| 8362 | ZCCHC3 | 1.042 | 0.199 | 12 | 0.0002 | 0.0062 | 2.06 | UP |
| 20760 | UBE2G2 | 1.042 | 0.208 | 12 | 0.0003 | 0.0073 | 2.06 | UP |
| 3733 | WIPF2 | 1.042 | 0.184 | 12 | 0.0001 | 0.0044 | 2.06 | UP |
| 35788 | SNRPE | 1.036 | 0.194 | 12 | 0.0002 | 0.0056 | 2.05 | UP |
| 49412 | FAM123B | 1.031 | 0.203 | 12 | 0.0003 | 0.0069 | 2.04 | UP |
| 43244 | TPM3 | 1.028 | 0.238 | 12 | 0.0010 | 0.0110 | 2.04 | UP |
| 58730 | EPB41L1 | 1.027 | 0.162 | 12 | <.0001 | 0.0023 | 2.04 | UP |
| 1961 | UBE2NL | 1.022 | 0.209 | 12 | 0.0004 | 0.0078 | 2.03 | UP |
| 60829 | LIMD1 | 1.022 | 0.193 | 12 | 0.0002 | 0.0059 | 2.03 | UP |
| 37728 | BTC | 1.020 | 0.171 | 12 | <.0001 | 0.0033 | 2.03 | UP |
| 50411 | SS18L2 | 1.020 | 0.106 | 12 | <.0001 | 0.0002 | 2.03 | UP |
| 50062 | SFT2D3 | 1.020 | 0.225 | 12 | 0.0007 | 0.0097 | 2.03 | UP |
| 36175 | RGS10 | 1.016 | 0.224 | 12 | 0.0007 | 0.0097 | 2.02 | UP |
| 3138 | WHAMM | 1.014 | 0.183 | 12 | 0.0001 | 0.0049 | 2.02 | UP |
| 21778 | CAMK2N1 | 1.013 | 0.166 | 12 | <.0001 | 0.0030 | 2.02 | UP |
| 11354 | TULP3 | 1.009 | 0.097 | 12 | <.0001 | 0.0001 | 2.01 | UP |
| 16526 | TYMS | 1.008 | 0.212 | 12 | 0.0005 | 0.0085 | 2.01 | UP |
| 39578 | ARPC2 | 1.006 | 0.222 | 12 | 0.0007 | 0.0098 | 2.01 | UP |
| 10446 | PXDN | 1.001 | 0.175 | 12 | <.0001 | 0.0042 | 2.00 | UP |
