# Supplementary material for: FOXM1 expression is significantly associated with chemotherapy resistance and adverse prognosis in non-serous epithelial ovarian cancer patients
Source: J Exp Clin Cancer Res. 2017 May 8;36:63. doi: 10.1186/s13046-017-0536-y (PMC5422964; doi:10.1186/s13046-017-0536-y)
Supplement: Supplementary file 11 — Functional analysis of the genome-wide transcriptional response in FOXM1-silenced EOC cells. Putative network reconstruction on EOC-CC1 and OSPC2 cells. (PDF 178 kb) [file 13046_2017_536_MOESM11_ESM.pdf]

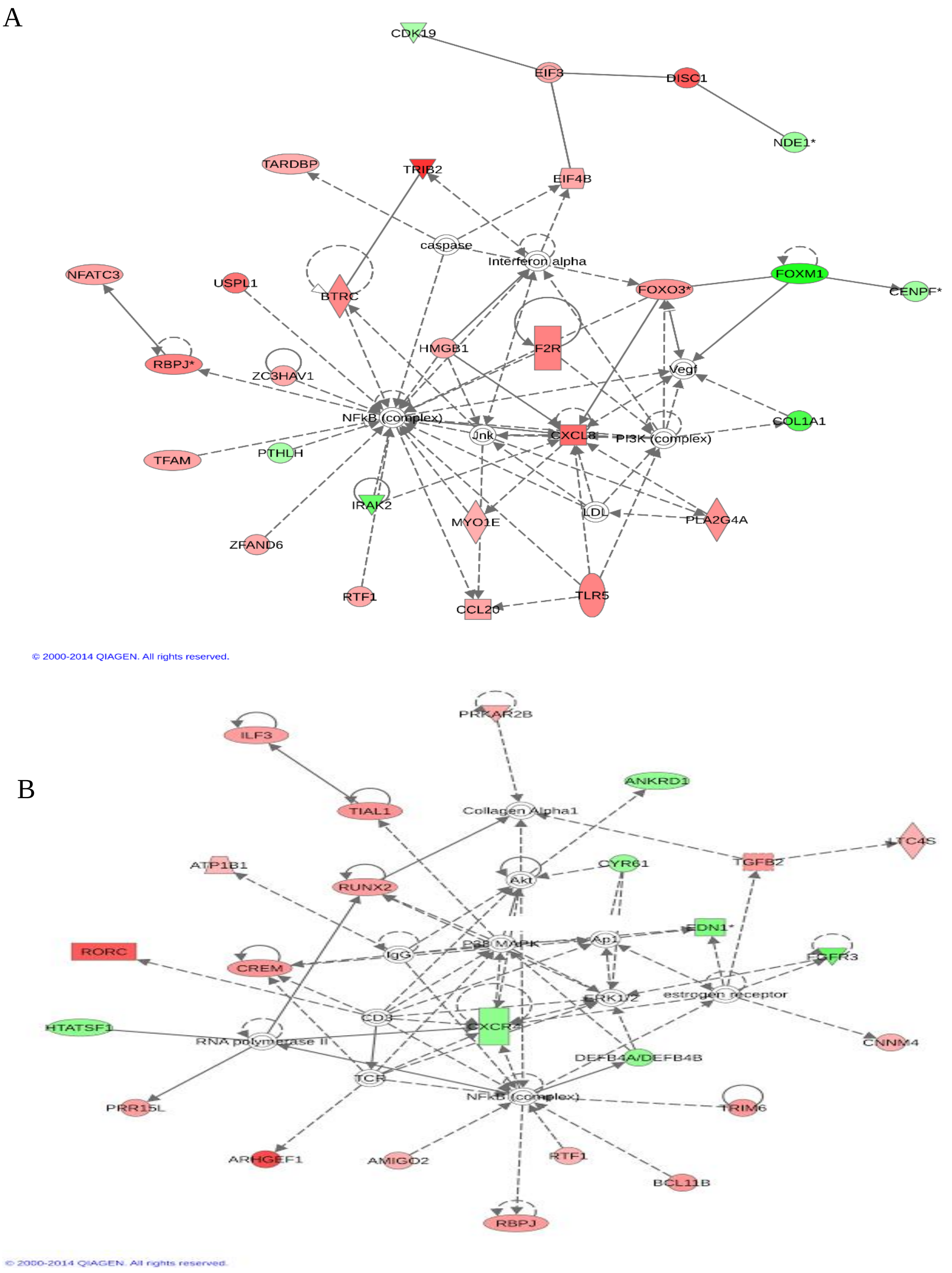

Figure S7. Functional analysis of the genome-wide transcriptional response in FOXM1-silenced EOC cells. Putative network reconstruction on EOC-CC1 cells (**A**) and on OSPC2 cells (**B**). Red, up-regulated genes (siFOXM1 versus scramble); green, down-regulated genes. The intensity of the node color indicated the degree of disregulation. Genes in uncolered notes were not identified as differentially expressed in our experiments and were integrated into the computationally generated networks on the basis of evidence stored in IPA database indicating a relevance to the network. Solid lines indicate direct relationships, while dashed lines indicate indirect relationships (Ingenuity® Pathway Analysis, Qiagen).
